# Nanoparticle conjugates of a highly potent toxin enhance safety and circumvent platinum resistance in ovarian cancer

**DOI:** 10.1038/s41467-017-02390-7

**Published:** 2017-12-18

**Authors:** Ruogu Qi, Yongheng Wang, Peter M. Bruno, Haihua Xiao, Yingjie Yu, Ting Li, Sam Lauffer, Wei Wei, Qixian Chen, Xiang Kang, Haiqin Song, Xi Yang, Xing Huang, Alexandre Detappe, Ursula Matulonis, David Pepin, Michael T. Hemann, Michael J. Birrer, P. Peter Ghoroghchian

**Affiliations:** 1 Koch Institute for Integrative Cancer Research at MIT, 500 Main Street, Cambridge, MA 02139 USA; 2 Massachusetts General Hospital, 55 Fruit Street, Boston, MA 02114 USA; 3 Dana Farber Cancer Institute, 450 Brookline Avenue, Boston, MA 02215 USA; 4 Harvard Medical School, 25 Shattuck Street, Boston, MA 02115 USA

**Keywords:** Nanoparticles, Nanoparticles, Chemotherapy, Chemotherapy, Ovarian cancer

## Abstract

Advanced-stage epithelial ovarian cancers are amongst the most difficult to treat tumors and have proven to be refractory to most cytotoxic, molecularly targeted, or immunotherapeutic approaches. Here, we report that nanoparticle-drug conjugates (NDCs) of monomethyl auristatin E (MMAE) significantly increase loading on a per-vehicle basis as compared to antibody-drug conjugates (ADCs). Their intraperitoneal administration enabled triggered release of the active MMAE toxin to inhibit tumor growth and to extend animal survival to >90 days in a cell-line xenograft model of disseminated ovarian cancer. In a patient-derived xenograft model of advanced-stage and platinum-resistant ovarian cancer, an MMAE-based NDC doubled the duration of tumor growth inhibition as compared to cisplatin. NDCs of highly potent toxins thus introduce a translatable platform that may be exploited to maximize the safety and efficacy of cytotoxic chemotherapies, combining the best features of ADCs with those of nanoparticle-based therapeutics.

## Abstract (web summary)

Improving the safety and efficacy of chemotherapeutics will help to enhance their effects. Here, the authors show that intraperitoneal delivery of nanoparticle conjugates of a potent toxin prolongs tumor inhibition and survival as compared to cisplatin in advanced-stage and platinum-resistant ovarian cancer mouse models.

## Introduction

Epithelial ovarian cancers are the seventh most common types of cancer in women worldwide, comprising an estimated 240,000 new cases per year and resulting in a 5-year overall survival rate that ranges from 30 to 50%^1^. While platinum-based anticancer agents are initially effective against high-grade serous ovarian cancers (HGSOC)^2^, which are the most common histological subtype^3^, recurrent “platinum-resistant” tumors have, thus far, proven refractory to most molecularly oriented and immunotherapeutic approaches. The development of novel agents with increased antitumor efficacy and limited toxicity is, thus, a critical unmet need^4^. Several strategies have recently emerged to improve outcomes by delivering chemotherapies in a targeted fashion^5^. These include the attachment of highly potent toxins to antibodies, forming antibody-drug conjugates (ADCs)^6^, and the encapsulation of existing small-molecule anticancer agents within nanoparticles (NPs)^7^. Several ADCs are currently in late stage clinical development for “platinum-resistant” HGSOCs (e.g., mirvetuximab soravtansine (IMGN853); ImmunoGen Inc., Waltham, MA)^8^. These agents typically carry 1–4 toxin molecules per antibody, are critically reliant on the properties of their drug linker, and can suffer from suboptimal tradeoffs that may limit their therapeutic indices^9^. For example, the dissociation of the toxin payload is necessary for antitumor activity but the prolonged circulation times of ADCs may lead to premature drug release, which results in sometimes significant off-target side effects^9^.

Similarly, the first generation of clinically-tested NPs have generally failed to significantly improve the therapeutic efficacy of their associated agents^10^. They have typically incorporated drugs with tolerable toxicity profiles such as doxorubicin (e.g., DOXIL® (doxorubicin HCl liposome injection); Johnson & Johnson) or paclitaxel (e.g., Abraxane® (paclitaxel protein-bound); Celgene), displaying modest activity against multiple cancer cell types (i.e., IC_50_s in the tens to hundreds of nM range)^11,12^. Additionally, they have generally relied on drug encapsulation as opposed to chemical conjugation; as a result, these NPs have displayed continuous drug release during their intravascular circulation^10^, which has led to persistent off-target side effects with only mild increases in efficacy^13^. While there are numerous examples of NP-drug conjugates in the literature, to date these formulations have also utilized either conventional or experimental small molecules with similar antitumor activities (i.e., 10–500 nM IC_50_s)^14,15^. As only 1–2 wt% of the injected NP dose is typically delivered to tumors after intravenous (IV) administration^16^, large amounts of carrier material are required for therapeutic efficacy, which has, hitherto, stymied clinical translation and/or induced material toxicities.

Here, we sought to conjugate highly potent toxins that display unprecedented activity against “platinum-resistant” HGSOCs (i.e., ones with single- or sub-nM IC_50_s) to NPs; note that a number of strategies have already been developed with ADCs to enable conjugation of highly potent toxins as prodrugs, which are bound through cleavable linkers^17^. We proposed that thousands of prodrug molecules could be similarly bound to a single NP, which would vastly increase potency as compared to ADCs on a per vehicle basis. Moreover, we have recently shown that intraperitoneal (IP) as opposed to IV injection of untargeted NPs resulted in near perfect intratumoral delivery in a murine model of disseminated ovarian cancer^18^. Both modes of administration are utilized clinically for the delivery of free drug formulations, and they yield identical circulatory half-lives (~12 h) for 100-nm-diameter NPs whose surfaces are comprised of 100% polyethylene glycol (PEG; 5 KDa)^18^. IP injection, however, results in the more rapid uptake of NPs into peritoneal tumor implants (~3 h)^18^. Given these findings, NP conjugation of toxins followed by their IP administration was pursued in order to maximize the in vivo stability of the carrier-bound prodrug, to prevent premature release, and to augment tumor uptake, which would help to avoid the systemic side effects seen with ADCs or with the first generation of clinically-tested NPs. IP delivery of NP conjugates enabled rapid uptake of toxins into the tumor environment, enabling effective utilization of tunable linker chemistries to optimize release properties, which aided in effective tumor growth inhibition and which markedly prolonged survival both in a disseminated tumor cell-line xenograft and in a patient-derived xenograft model of advanced-stage and resistant HGSOC.

## Results

### Design of a reductive-sensitive and self-immolative linker that incorporates MMAE as a prodrug

**Fig. 1:**
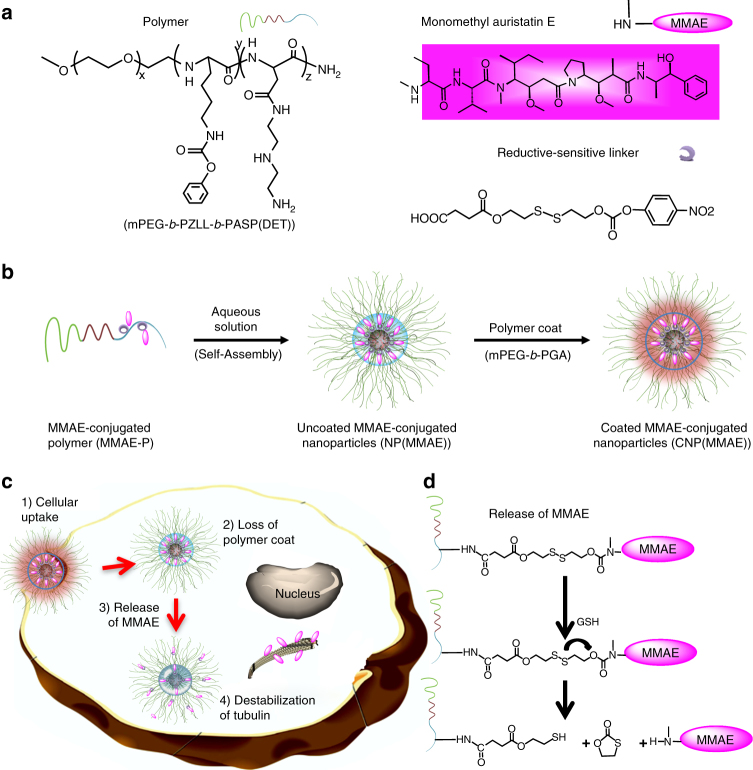
MMAE-conjugated nanoparticles enable intracellular release within cancer cells. **a** Structures of the biodegradable and cationically-charged polymer of methoxy-poly(ethylene glycol)-*block*-poly(carbobenzyloxy-L-lysine)-*block*-poly{N-[N-(2-aminoethyl)-2-aminoethyl]aspartamide} (mPEG-*b*-PZLL-*b-*PASP(DET)), the highly potent microtubule inhibitor monomethyl auristatin E (MMAE), and a reductive-sensitive linker. **b** Aqueous dissolution of MMAE-conjugated polymer (MMAE-P) leads to the spontaneous self-assembly of MMAE-conjugated nanoparticles (NP(MMAE)); further complexation of the water-soluble and polyanionic diblock copolymer of methoxy-poly(ethylene glycol)-*block*-poly(glutamic acid) (mPEG-*b*-PGA) aids to stabilize these coated nanoparticles (CNP(MMAE)). **c** Intracellular uptake and release of MMAE from CNP(MMAE). **d** Mechanism of the release of free MMAE from MMAE-P, which is driven by high intracellular concentrations of reducing agents such as glutathione (GSH; 5 mM intracellular vs. 25–50 µM in the extracellular milieu)

To generate our NP conjugates, we synthesized a novel triblock copolymer of methoxypoly(ethylene glycol)-*block*-poly(carbobenzyloxy-L-lysine)-*block*-poly{*N*-[*N*-(2-aminoethyl)-2-aminoethyl]aspartamide} (mPEG-*b*-PZLL-*b*-PASP(DET)) that self-assembles into a biodegradable NP with a hydrophilic mPEG surface, a hydrophobic PZLL core, and a cationic polypeptide corona comprised of PASP(DET); this latter block was utilized for covalent conjugation of a multitude of highly potent toxins—each bound through a central reductive-sensitive and self-immolative linker (Fig. 1a). As an example toxin species, we chose the antimitotic agent monomethyl auristatin E (MMAE), which is known to inhibit cellular division by blocking the depolymerization of tubulin^19^. MMAE has been shown to be 10–100× more potent than doxorubicin (in vitro IC_50_ = 0.2–0.6 nM) and is widely used for the development of ADCs and small-molecule drug conjugates (SMDCs)^20^. Brentuximab vedotin (Adcetris®, Seattle Genetics) was the first FDA-approved ADC and consists of an anti-CD30 antibody that is coupled to MMAE through a cathepsin (i.e., enzyme-sensitive) cleavable linker. Despite its improved activity against CD30-positive lymphomas, a significant number of patients still suffer from peripheral neuropathy, myelosuppression, fatigue, and gastrointestinal disturbances that result from premature loss of free MMAE from the ADC ^21^.

For our studies, the cationic surface charge of the MMAE-conjugated NP (NP(MMAE)) formed from mPEG-*b*-PZLL-*b*-PASP(DET)-coupled MMAE (MMAE-P) was neutralized through layer-by-layer deposition of anionically-charged diblock copolymers of methoxyl-poly(ethylene glycol)-*block*-poly(glutamic acid) (mPEG-*b*-PGA), generating a coated and MMAE-conjugated NP (CNP(MMAE)) that was expected to prevent premature drug release and to enable tumor-specific uptake (Fig. 1b). The mPEG-*b*-PGA coat was designed to dissociate from the core NP(MMAE) in low pH environments such as those found in the tumor microenvironment or intracellularly within tumor cells. It is important to note that the anionic charge of PGA is known to be neutralized at a pH below its pKa^22^; and cellular uptake and endosomal escape of cationically-charged NP(MMAE) ensues. Release of free MMAE occurs in the cytoplasm and proceeds in a triggered fashion (Fig. 1c). With respect to the mechanism(s) of MMAE release, the linker was designed to contain a disulfide bond whose reduction is triggered by the high intracellular concentrations of glutathione (GSH) found within tumor cells^23^. This then results in spontaneous nucleophilic attack by the free thiol on the carbamate bond that couples the MMAE prodrug, releasing the active toxin in its unmodified form (Fig. 1d). This specific design strategy was selected to decrease the intravascular release of the free drug so as to limit its systemic side effects. We sought to further validate that IP administration of CNP(MMAE) could promote antitumor efficacy and improve upon the safety of MMAE-based ADCs.

While disulfide-based linkers are able to exploit high reducing conditions within tumor cells to promote toxin delivery (e.g., mM concentrations of intracellular GSH as opposed to μM concentrations in the extracellular milieu^24^), this class of reductive-sensitive linkers has often proven to be too labile for safe administration; prolonged ADC circulation may lead to premature loss of toxin when coupled through a linker containing a disulfide group^25^. We hypothesized that such linkers, however, could be successfully utilized for conjugation of toxins to NPs given the relatively shorter circulatory lifetimes and more rapid tumor accumulation that are imparted by the larger sizes of these synthetic delivery vehicles (when compared to antibodies). To test this hypothesis, we synthesized a reductive-sensitive and self-immolative linker with a central disulfide group that was bound to MMAE through a carbamate bond (Fig. 1d and Supplementary Figs. 1–3). The structure and purity of the intermediates (Compounds 1 and 2) and of the final MMAE-conjugated linker (Compound 3; i.e., MMAE-prodrug) were verified by nuclear magnetic resonance spectroscopy (^1^H and ^13^C NMR; Supplementary Figs. 4–9) and by high-pressure liquid chromatography (HPLC; Supplementary Fig. 10).

**Fig. 2:**
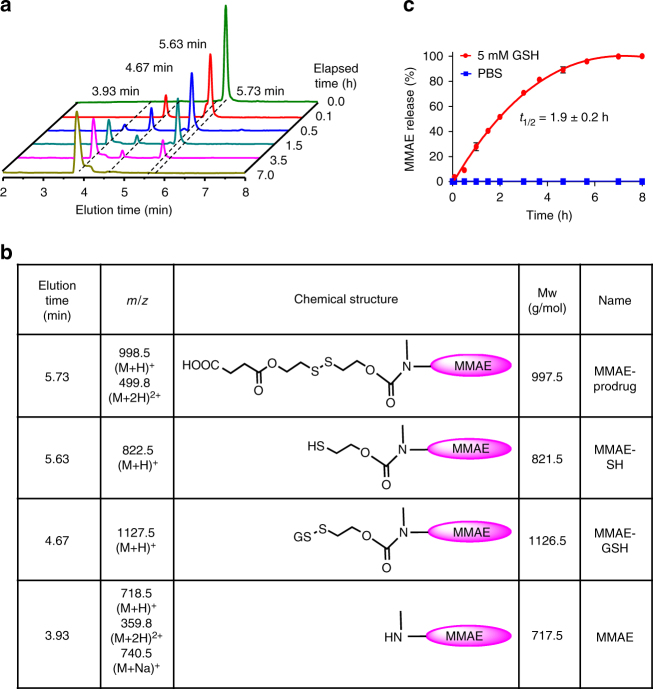
In situ kinetics for release for MMAE from the reductive-sensitive linker. **a** UV–Vis detection of different elution bands corresponding to the products formed from the reaction of the MMAE-bound and reductive-sensitive linker with 5 mM glutathione (GSH) as determined by LC–MS. **b** Retention times, mass-to-charge ratios (*m*/*z*), chemical structures, and the calculated masses of the isolated products from each of the different elution bands corresponding to the traces in **a**. **c** Rates of release of MMAE from the reductive-sensitive linker in phosphate saline buffer (PBS; pH 7.4, 37 °C; blue) and after the addition of GSH (5 mM; red)

To demonstrate the ability of the self-immolative linker to enable triggered release of the free drug in its original form, we dissolved the MMAE-prodrug in phosphate buffered saline (PBS, pH 7.4), added exogenous GSH (5 mM), and incubated the mixture at 37 °C. These in situ conditions were selected to mimic the in vivo environment within tumor cells that would result in the cleavage of the disulfide-containing linker with concomitant release of the toxin. At various time points, liquid chromatography–mass spectrometry (LC–MS) of solution aliquots were taken to monitor for the presence of different reaction intermediates, which were identified from their positive mode mass spectra. The results demonstrated the rapid disappearance of the MMAE-prodrug (elution peak at *t* = 5.73 min) after 30 min of incubation with GSH (5 mM) and exhibited the emergence of three new compounds with peak elution times at 5.63, 4.67, and 3.93 min, respectively (Fig. 2a). These peaks corresponded to the MMAE-prodrug bound to GSH (Compound 4; MMAE-GSH, *t* = 4.67 min), a sulfhydryl-modified MMAE intermediate (Compound 5; MMAE-SH, *t* = 5.73 min), and free MMAE (MMAE, *t* = 3.93 min), whose structures were assigned from the measured mass-to-charge ratios and from the calculated molecular weights of the compounds (Fig. 2b and Supplementary Fig. 11). By plotting the changes in the values for the absorption peak corresponding to the MMAE-prodrug (*t* = 5.73 min) vs. the elapsed time after GSH addition, the rate of free MMAE release from the linker was obtained (Fig. 2c, red). The results demonstrate that the MMAE-prodrug is completely converted to free MMAE under high reducing conditions, exhibiting a transformation half-life of 1.9 ± 0.2 h. In the absence of exogenous GSH, the chromatogram of the MMAE-prodrug was unchanged after 7 h of dissolution in PBS alone (pH 7.4), supporting the stability of the linker under physiological conditions (Fig. 2c, blue).

### Development of MMAE-conjugated NPs and characterization of their material properties

In order to generate NP(MMAE), we focused on synthesizing an NP formulation that would allow for ready coupling to our MMAE-prodrug. The triblock copolymer of mPEG_114_-*b*-PZLL_6_-*b*-PBLA_30_ was synthetized via ring-opening polymerization, using mPEG(5 KDa)-NH_2_ as the initiator, by combining Z-Lys-NCA and Bzl-Asp-NCA in dimethylformamide (DMF), and by modification of a previously reported method (Supplementary Fig. 12)^26^. Upon addition of diethylenetriamine, aminolysis of mPEG_114_-*b*-PZLL_6_-*b*-PBLA_30_ yielded mPEG_114_-*b*-PZLL_6_-*b*-PASP(DET)_30_ (Supplementary Fig. 13). This latter polymer was further coupled to the MMAE-prodrug, using EDC/NHS chemistry, to yield an MMAE-conjugated polymer that contained two MMAE molecules per polymeric chain (mPEG_114_-*b*-PZLL_6_-*b*-PASP(DET)_30—_MMAE_2_, i.e., MMAE-P (Supplementary Fig. 14). The polymerization ratios of each constitutive block in mPEG_114_-*b*-PZLL_6_, mPEG_114_-*b*-PZLL_6_-*b*-PBLA_30_, mPEG_114_-*b*-PZLL_6_-*b*-PASP(DET)_30_, and MMAE-P were calculated from their ^1^H NMR spectra (Supplementary Figs. 15–18); and, the polydispersity indexes (PDIs) and molecular weights of the copolymers were measured by gel permeation chromatography (GPC; Supplementary Fig. 19 and Supplementary Table 1).

Aqueous dissolution of mPEG_114_-*b*-PZLL_6_-*b*-PASP(DET)_30_ and MMAE-P led to spontaneous self-assembly of an unmodified NP and NP(MMAE), respectively (Supplementary Fig. 20 and Supplementary Table 2). The hydrodynamic diameter of NP(MMAE) was found to be 93.5 ± 7.4 nm by dynamic light scattering (DLS) while its core diameter was determined to be 53.7 ± 7.3 nm by transmission electron microscopy (TEM). Note that the unmodified NP formed from mPEG_114_-*b*-PZLL_6_-*b*-PASP(DET)_30_ had a hydrodynamic diameter of 120.1 ± 4.2 nm and a core diameter of 34.2 ± 5.7 nm. Taken together, these data support the segregation of the PASP-coupled MMAE-prodrug within the PZLL core of NP(MMAE), increasing its core diameter as compared to the unmodified NP. This leads to the depletion of PASP(DET) chains from the surface, which reduces the overall hydrodynamic diameter of NP(MMAE) with respect to that of the unmodified NP. Given that there is one PASP(DET) and one mPEG block per PZLL chain, that there are ~2 MMAE molecules per PASP(DET) block in MMAE-P, and assuming an interfacial area of ~1 nm^2^ per PZLL chain in an unmodified NP, NP(MMAE) contains at least ~2500–5000 MMAE molecules per particle. For dosing purposes, NP(MMAE) is comprised of ~10% MMAE by weight. As drug to antibody ratios (DARs) of 1–4 are typically employed in ADC development^27^, an MMAE-based ADC may be comprised of at most 2% weight MMAE (at a DAR = 4). Thus, both the numbers of MMAE molecules and the percentages of the resultant vehicles that are comprised of MMAE are significantly greater for NP(MMAE) when compared to clinically relevant MMAE-based ADCs.

Having validated drug loading levels, we next sought to determine the stability and triggered release capabilities of NP(MMAE). Release of free MMAE from NP(MMAE) was confirmed under high reducing conditions (PBS, 5 mM GSH), using LC–MS, which demonstrated elution times, mass-to-charge ratios, and transformation lifetimes for reaction intermediates that were nearly identical to those observed upon GSH addition to the uncoupled MMAE-prodrug (Supplementary Fig. 21A–B). Note that the presence of the sulfhydryl-containing MMAE intermediate (Compound 5) was not, however, detected in suspensions of NP(MMAE) after addition of exogenous GSH. The disulfide bond in the MMAE-prodrug linker of NP(MMAE) was cleaved after 5 min. Thereafter, free MMAE was released through spontaneous nucleophilic attack of the self-immolative linker. Under identical solution conditions but in the absence of exogenously added GSH (i.e., PBS, pH 7.4), there were no observed peaks in the HPLC spectrum that corresponded to free MMAE or to the uncoupled MMAE-prodrug (Supplementary Fig. 21C). The results support the stability of NP(MMAE) under physiological conditions, such as may be found in the vasculature, and confirm rapid release of free MMAE in highly-reductive environments, which may be found intracellularly after NP uptake into tumor cells.

While MMAE conjugation results in consumption of some of the cationically-charged PASP chains from the surface of the unmodified NP, the zeta potential of NP(MMAE) is still positive (+18.7 mV). To minimize non-specific biological adhesion, as well as to prevent premature opsonization that would compromise in vivo tumor delivery by promoting more rapid phagocytosis by cells of the reticuloendothelial system (RES)^28^, the surface charge of NP(MMAE) must be neutralized. To address this issue, we employed layer-by-layer assembly to complex an anionically-charged polymer of mPEG_114_-*b*-PGA_30_ (Supplementary Table 1) with the cationically-charged NP(MMAE) (Supplementary Fig. 22). By increasing the initial molar ratio of negatively-charged mPEG_114_-*b*-PGA_30_ to positively-charged MMAE-P (i.e., the *N*/*P* ratio), we found that we could effectively decrease both the average hydrodynamic diameter and the magnitude of the positive surface charge of the resultant coated and MMAE-conjugated NP (CNP(MMAE)). A centrifugation filtration step was further employed to remove any non-complexed (i.e., free) mPEG_114_-*b*-PGA_30_. CNP(MMAE) suspensions formed at an *N*/*P* ratio of 1 resulted in minimization of the average hydrodynamic diameter (from 93.5 nm for NP(MMAE) to 68.5 nm for CNP(MMAE); Supplementary Fig. 22A) and in effective neutralization of the surface charge (from +18.7 mV for NP(MMAE) to +0.8 mV for CNP(MMAE); Supplementary Fig. 22B). The *N*/*P* ratio of 1 was thereafter adopted for all further experiments with CNP(MMAE). CNP(MMAE) is thus comprised of ~6% MMAE by weight.

For effective therapeutic application, it is imperative to validate the colloidal stability of NP formulations as aggregation during physiological conditions leads to poor in vivo performance. Serial DLS (Supplementary Fig. 22C) and zeta potential measurements (Supplementary Fig. 22D) of small volume aliquots of NP(MMAE) and CNP(MMAE) in HEPES buffer were taken at 37 ^o^C and at 12 h intervals over the course of 48 h. While NP(MMAE) experienced a loss in both its hydrodynamic diameter and surface charge over time, which was likely attributable to non-enzymatic hydrolysis of PASP chains from its surface, CNP(MMAE) displayed consistent properties under the same conditions. The improved stability of CNP(MMAE) when compared to NP(MMAE) may be attributed to the dense surface brush imparted by its mPEG_114_-*b*-PGA_30_ coating, which stabilizes the assembly and further prevents loss of the PASP-coupled MMAE-prodrug.

### In vitro uptake of MMAE-conjugated NPs into ovarian cancer cells

The intracellular delivery of CNP(MMAE) into ovarian cancer cells was examined, using the established OVCAR8 cell line that has been shown to possess a gene expression signature that resembles that of HGSOC^29^. The mPEG_114_-*b*-PGA_30_ coat of CNP(MMAE) was labeled with the fluorophore 5′-carboxyfluorescein (5′-FAM; *λ*_ex_ = 492 nm, *λ*_em_ = 518 nm; green) while the core NP(MMAE) was conjugated with Cy5.5™ (Cy5.5; *λ*_ex_ = 675 nm, *λ*_em_ = 695 nm; red), using EDC/NHS chemistry. These dual-fluorophore-labeled, coated, and MMAE-containing NPs (5′-FAM-CNP(MMAE/Cy5.5)) were then incubated with OVCAR8 cells for different durations of time, and intracellular uptake of each component was independently monitored, using confocal laser scanning fluorescence microscopy (Supplementary Fig. 23). The time-dependent increases in both red and green fluorescence and the nearly perfect co-localization of the two colors over time supported the stability of CNP(MMAE) during the cellular internalization process.

To assess for any effects on intracellular uptake imparted by the mPEG_114_-*b*-PGA_30_ diblock copolymer, OVCAR8 cells were incubated with either Cy5.5-labeled NP(MMAE) (NP(MMAE/Cy5.5)) or Cy5.5-labeled CNP(MMAE) formulations (CNP(MMAE/Cy5.5)) and confocal laser scanning fluorescence microscopy was again performed at different time points (Supplementary Fig. 24). These qualitative results, which showed a time-dependent increase in fluorescence within a punctate distribution pattern throughout the cytoplasm of OVCAR8 cells that had been treated with either MMAE-containing NP, were further supported by quantitative comparisons of intracellular uptake by flow cytometry (Supplementary Fig. 25). By gating on the populations of Cy5.5-labeled OVCAR8 cells over time, it was evident that mPEG_114_-*b*-PGA_30_ had at most a modest effect on decreasing the rate of NP uptake. OVCAR8 cells that had been incubated with CNP(MMAE/Cy5.5) displayed approximately 2/3 of the mean Cy5.5 fluorescence intensity of cells that had been treated with NP(MMAE/Cy5.5) for 2 h. When examined in total, these confocal and flow cytometry experiments demonstrate the intact uptake of CNP(MMAE) by OVCAR8 cells with minimal effects from its mPEG_114_-*b*-PGA_30_ coating on influencing the rate and extent of the cellular internalization process.

### In vitro activity of different MMAE-conjugated nanoparticle formulations

**Fig. 3:**
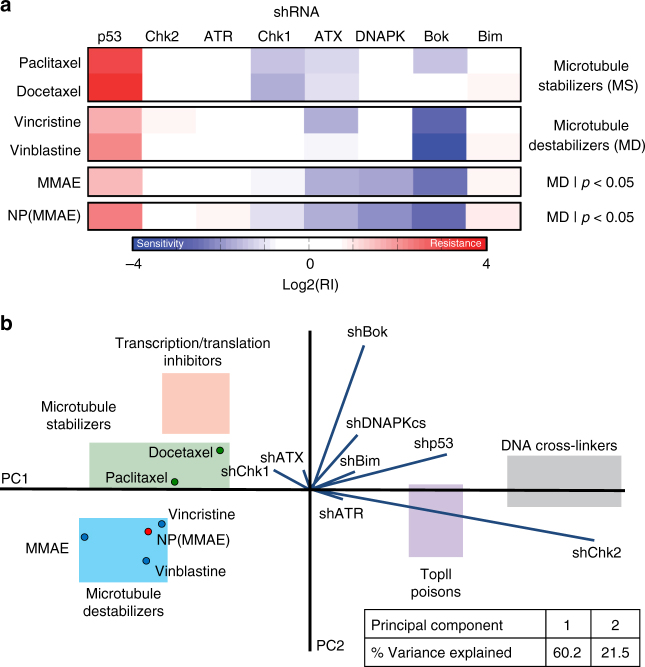
Therapeutic classification of MMAE-conjugated nanoparticles using an shRNA screen. **a** Heat maps and RNAi signature classifications of conventional microtubule stabilizers (paclitaxel, docetaxel) and microtubule destabilizers (vincristine, vinblastine), the highly potent toxin MMAE, and its nanoparticle conjugate (NP(MMAE)). **b** Principal component analysis of RNAi signatures from each of these aforementioned agents as well as in relation to known transcription/translation inhibitors, topoisomerase II (topII) poisons, and the DNA cross-linkers reference sets

As chemical conjugation of small-molecule anticancer agents to either antibodies^30^ or NPs^31^ have been shown to potentially alter their mechanistic activities, we utilized an established RNA interference (RNAi) screen^31–34^ to study changes in the intracellular behavior of free MMAE that could be imparted by NP conjugation. This RNAi screen uses an established cMyc-driven lymphoma cell line that is partially infected with one of eight different green fluorescent protein (GFP)-labeled short hairpin RNAs (shRNAs) that confer either resistance or sensitivity to administered therapeutic agents, depending on their mechanisms of action. After treatment with a given anticancer agent, the cells are subject to flow cytometry to assess for the percentages of cellular GFP expression. The resultant patterns of resistance or sensitivity that are imparted by the different shRNAs are then processed by a probabilistic K-nearest neighbor (K-NN) algorithm that assigns novel compounds to a category by comparing their signatures to that of a reference set of drugs. The RNAi signatures of free MMAE and NP(MMAE) were compared to one another, to those of paclitaxel and docetaxel (two conventional microtubule stabilizers), as well as to those of vincristine and vinblastine (two known microtubule destabilizers) (Fig. 3a). Using the modified K-NN algorithm, MMAE and NP(MMAE) were categorized as microtubule destabilizers (Fig. 3b). Additionally, principal component analysis, which allows one to further appreciate how shp53 enrichment and shBok depletion affect the activities of MMAE or NP(MMAE), helped to classify the two agents as microtubule destabilizers. The results of this shRNA screen thus confirm that NP-conjugation of MMAE does not affect its mechanistic behavior and that the active toxin is released within tumor cells.

**Fig. 4:**
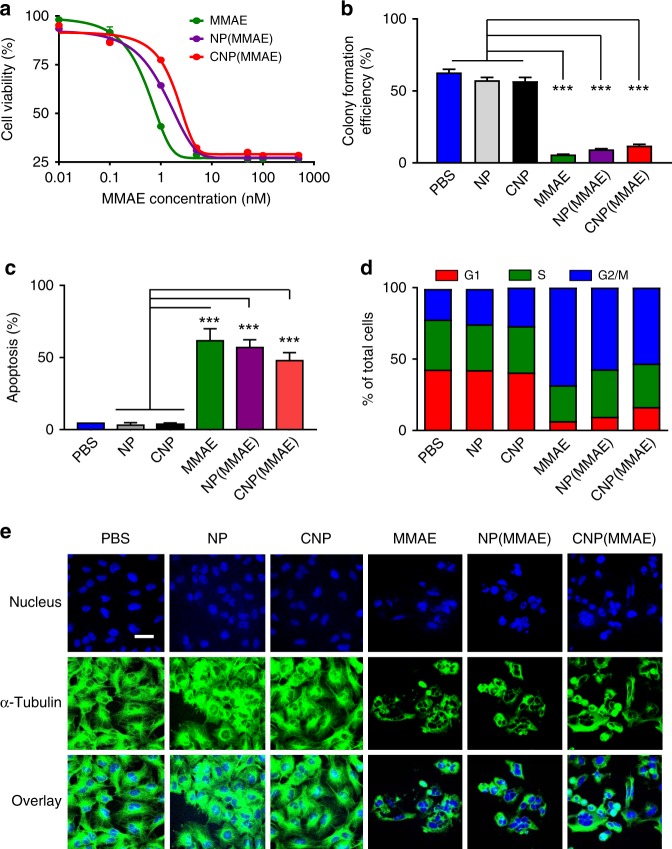
In vitro activity of MMAE-conjugated nanoparticles. **a** Relative cellular viability of OVCAR8 ovarian cancer cells after 72 h of incubation with free MMAE, uncoated and MMAE-conjugated nanoparticles (NP(MMAE)), or coated and MMAE-conjugated nanoparticles (CNP(MMAE)). **b** Relative efficiency of colony formation for OVCAR8 cells at 7 days after cellular exposure to free MMAE, NP(MMAE), or CNP(MMAE) and in comparison to various control treatments (PBS, empty nanoparticles (NP), and empty coated nanoparticles (CNP)). Flow cytometry measurements were performed to determine **c** the percentages of cells undergoing apoptosis and **d** the cell-cycle distribution of OVCAR8 cells at 48 h after continuous exposure to different MMAE-containing formulations or various controls. **e** α-Tubulin immuno-detection by confocal microscopy, confirming the preserved ability of MMAE-conjugated nanoparticles (NP(MMAE) and CNP(MMAE)) to enable destabilization of tubulin within the cytoskeleton of OVCAR8 cells and as compared to the cellular responses to free MMAE. Scale bar = 40 μm (***p-value < 0.001, unpaired *t*-test)

We next sought to examine the in vitro potency of different MMAE-conjugated NPs. A panel of ovarian cancer cell lines (A2780, COV362, COV318, OVCAR4, and OVCAR8) was used to screen for the relative cytotoxicity of NP(MMAE) as compared to CNP(MMAE)). The cells were incubated with each formulation for 72 h and relative cellular viability was thereafter determined, using the colorimetric MTT assay. The results were compared to the free drug formulation of MMAE (MMAE) on an equimolar basis of active toxin (Fig. 4a and Supplementary Fig. 26). By evaluating the concentrations of each agent (on a molar basis of MMAE) that inhibited cellular viability by 50% after 72 h (i.e., the IC_50_ values), a clear trend emerged: the relative potency of free MMAE > NP(MMAE) > CNP(MMAE) for each cell line (Supplementary Table 3).

While the IC_50_ values for free MMAE were generally consistent across the examined cell lines, ranging from 0.24 to 0.92 nM (when excluding A2780, which had an IC_50_ value of 3.65 nM for free MMAE), the potencies of NP(MMAE) varied within one order of magnitude, displaying IC_50_ values between 0.87 to 3.86 nM. As the intracellular concentrations of reducing agents (e.g., GSH and/or ascorbic acid) within tumor cells have been shown to be 100- to 1000-fold higher than those found within normal cells or in the extracellular milieu^35–39^, these results may be attributable to the slight differences in the relative intracellular concentrations of reducing agent that are found in the various ovarian cancer cell lines, which lead to triggered release of free MMAE from an MMAE-conjugated NP. In all cases, the IC_50_ values of CNP(MMAE) were approximately 1–4 fold higher than those of NP(MMAE), which indicate a mild decrease in potency upon coating of NPs with mPEG_114_-*b*-PGA_30_; the polymeric constituents of the NPs had no cytotoxic effects on the cells in the absence of MMAE conjugation (Supplementary Fig. 27). Despite slight reductions in potency on a per molecule basis of toxin, the IC_50_ values for CNP(MMAE) were still in the single nM range for each of the examined cell lines (again excluding A2780). It is important to underscore that thousands of MMAE molecules are bound to a single NP, which vastly increases the relative cytotoxicity on a per macromolecule basis when compared to the free drug formulation or to any reported MMAE-based ADC.

The in vitro antiproliferative activity of CNP(MMAE) was further confirmed, using a colony formation assay that was performed on OVCAR8 cells that were treated with various NP formulations or with free MMAE (Fig. 4b and Supplementary Fig. 28). Statistically significant reductions in colony formation were evident after 7 days of treatment with either free MMAE, NP(MMAE), or CNP(MMAE) (at equimolar concentrations of toxin) when compared to controls. While the differences were not statistically significant, there remained a correlation whereby the relative potency of free MMAE > NP(MMAE) > CNP(MMAE) by this assay. Determination of the levels of apoptosis by flow cytometry of OVCAR8 cells after 48 h of incubation with the same experimental and control agents showed similar trends (Fig. 4c and Supplementary Fig. 29): all MMAE-containing treatments significantly increased the levels of apoptosis when compared to controls. While the levels of apoptosis induced by treatment of OVCAR8 cells with CNP(MMAE) were less those of NP(MMAE) and free MMAE, the total numbers of apoptotic events in the CNP(MMAE) group were at ~75% of the levels imparted by treatment with free MMAE (Supplementary Table 4).

The cell-cycle distribution of OVCAR8 cells showed a predominance of G2/M phase arrest after treatment with any MMAE-containing group (Fig. 4d and Supplementary Fig. 30), which was consistent with the known mechanism of tubulin destabilization and growth arrest induced by MMAE. The percentages of cells in G2/M phase after treatment with CNP(MMAE) were at ~78% of the levels imparted by free MMAE (at equal concentrations of toxin; Supplementary Table 5). To further validate the biological consequences of CNP(MMAE) treatment with respect to NP(MMAE), free MMAE, and various control groups, confocal microscopy experiments of OVCAR8 cells were conducted after staining for intracellular tubulin, using an Alexa488-labeled antitubulin antibody (Fig. 4e). Incubation with any MMAE-containing group led to substantial disruption in the microtubule network of the cells, causing them to round up into clusters that then underwent apoptosis. Together, these results indicate that any reductions in the relative potency of MMAE after NP conjugation are not biologically relevant and are likely counteracted by the increased numbers of MMAE molecules that are delivered intracellularly by a single NP.

### In vivo safety and antitumor efficacy of MMAE-conjugated nanoparticles

While important to the success of any in vivo delivery system, mitigation of potential off-target toxicities is a critical challenge that must be addressed to ensure the safe administration of a highly potent toxin. To examine effects on healthy tissues, dose escalation studies with CNP(MMAE) were undertaken in healthy (4–6 week old) female BALB/c mice. Mice were randomly assigned (*n* = 3 mice/group) and were treated with a single dose of one of the following by IP administration: PBS, free MMAE (MMAE; at 0.25 or 0.5 mg/kg, the latter of which corresponded to its known maximum tolerated dose (MTD) after a single administration)^40^, or CNP(MMAE) (at either a 1- or 3-mg/kg dose equivalent of free MMAE). It should be noted that the 3-mg/kg dose equivalent of free MMAE was selected to test for the enhanced safety of CNP(MMAE) as compared to auristatin-based ADCs, which have repeatedly been shown to induce significant toxicities after a single administration at dose equivalents that are greater than 1.1–2.3 mg/kg of toxin^19,27,40,41^ (Supplementary Table 6). The mice were monitored and weighed daily, and they were sacrificed when they exhibited >15% loss in body weight or at 14 days after treatment administration (Supplementary Fig. 31A). At the time of sacrifice, terminal blood draws were taken for renal function studies (Supplementary Fig. 31B), liver function tests (LFTs; Supplementary Fig. 31C), completed blood counts (CBC; Supplementary Fig. 31D), and white blood cell differential counts (Supplementary Fig. 31E); major organs were also harvested for H&E analyses (Supplementary Fig. 32). The results demonstrated no toxic effects from CNP(MMAE) that was administered at the 3-mg/kg dose equivalent of free MMAE, establishing the augmented safety of toxins that are delivered by NPs via this route.

**Fig. 5:**
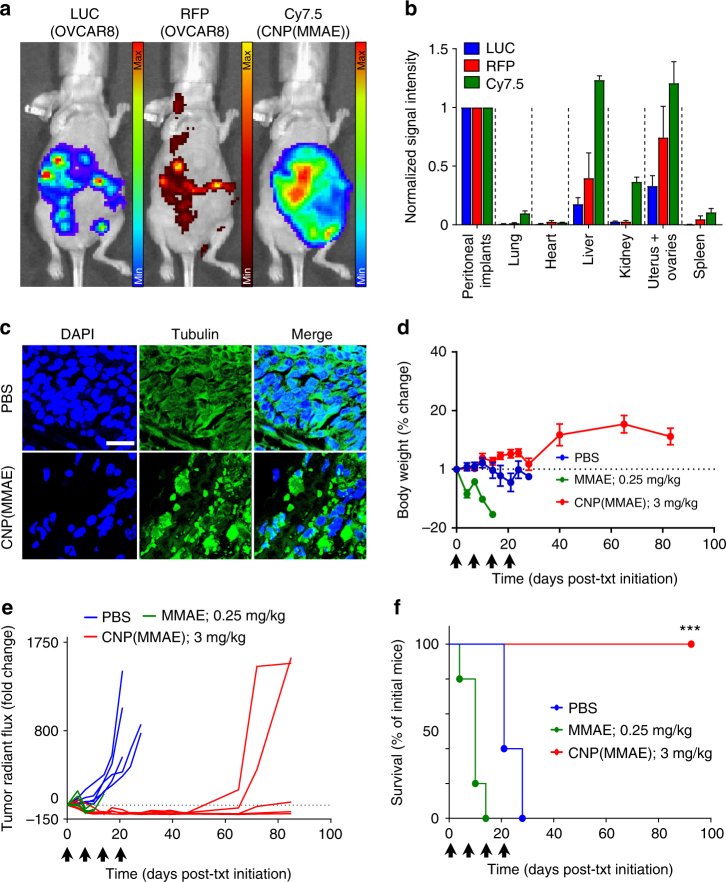
On-target activity and therapeutic efficacy of MMAE-conjugated nanoparticles in an orthotopic cell-line xenograft model of disseminated ovarian cancer. **a** Representative in vivo images of a single LUC^+^/RFP^+^ OVCAR8 tumor-bearing nude mouse at 72 h after IP administration of coated, Cy7.5-labeled, and MMAE-conjugated nanoparticles (CNP(MMAE/Cy7.5)). **b** Ex vivo signal intensities in each organ at the time of animal sacrifice (*n* = 3 mice/group). Signal intensity was normalized to the value measured from the intestines of each animal, which had high burdens of micrometastatic tumor foci. **c** Immunofluorescence staining for α-tubulin in OVCAR8 tumor implants excised from nude mice at 72 h after IP administration of CNP(MMAE) or PBS (control). **d** Changes in the body weights as compared to baseline. **e** Tumor burden over time as determined by changes from the baseline radiant flux associated with the BLI signal intensity. **f** Survival of OVCAR8 tumor-bearing nude mice that received ×4 weekly IP injections of CNP(MMAE) (at an equivalent dose of 3 mg/kg MMAE), free MMAE (at 0.25 mg/kg), or PBS (control treatment). The black arrows indicate the timing of each dose of treatment. The CNP(MMAE) group demonstrated a statistically significant improvement in survival as compared to mice that received either PBS and free MMAE (***p-value < 0.001, Log-rank test)

Biodistribution studies were conducted by optical imaging of nude mice bearing disseminated LUC^+^ OVCAR8 tumors both before and after IP delivery of CNP(MMAE), which was fluorescently tagged with Cy7.5 (CNP(MMAE/Cy7.5)); they confirmed high tumor accumulation of NPs after their IP administration (Fig. 5a and Supplementary Fig. 33A). Ex vivo imaging performed on excised organs at the time of animal sacrifice confirmed the co-localization of luminescent and fluorescent signals within tumor deposits (Fig. 5b and Supplementary Fig. 33B). Note that the majority of the administered dose of CNP(MMAE/Cy7.5) was found within peritoneal tumor implants; the liver was the only healthy organ exhibiting significant uptake, which was at comparable levels. To verify on-target biological activity, separate nude mice, bearing disseminated OVCAR8 tumors, were sacrificed at 72 h after the administration of the second of two weekly doses of CNP(MMAE); their tumors were harvested, and immunofluorescence staining for α-tubulin was performed, demonstrating disruption of the cellular cytoskeleton (Fig. 5c).

Therapeutic testing commenced by dosing additional nude mice, bearing disseminated LUC^+^ OVCAR8 tumors, with four weekly IP injections of CNP(MMAE) (at the 3-mg/kg dose-equivalent level of MMAE)) or controls (PBS or free MMAE at 0.25 mg/kg). Changes in body weight were monitored at biweekly intervals (Fig. 5d) and tumor growth was visualized by serial BLI measurements (Supplementary Fig. 34). Mice that had been treated with free MMAE exhibited statistically significant reductions in their tumor burdens as compared to those that had received PBS (Fig. 5e and Supplementary Fig. 34); but, they all experienced profound weight loss and expired quickly (Fig. 4f), presumably due to MMAE toxicity. Multi-dose administration of CNP(MMAE) at the 3-mg/kg dose-equivalent level of MMAE, which exceeded the single-dose MTD of all auristatin-based ADCs^19,27,40,41^ (Supplementary Table 6), did not induce a loss in body weight. It did, however, substantially inhibit tumor growth and extend animal survival to >90 days.

### Overcoming platinum resistance in patient-derived, advanced-stage HGSOC

**Fig. 6:**
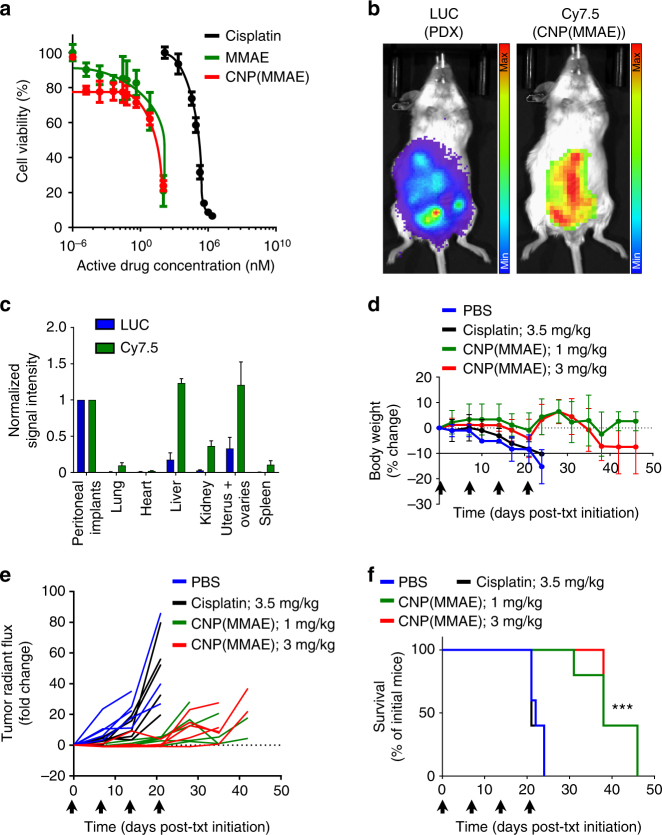
Therapeutic activity of MMAE-conjugated nanoparticles against platinum-resistant and high-grade serous ovarian cancer (HGSOC). **a** Primary HGSOC cells were cultured and treated with either cisplatin, free MMAE, or coated and MMAE-conjugated nanoparticles (CNP(MMAE)) for 72 h prior to cellular viability measurements by the colorimetric CCK8 assay; the results were compared to those obtained from untreated cells. **b** Representative in vivo images of a single C.B-17/Icr-SCID/Sed mouse implanted with LUC^+^ primary HGSOC cells (LUC^+^ PDX model) at 72 h after IP administration of coated, Cy7.5-labeled, and MMAE-conjugated nanoparticles (CNP(MMAE/Cy7.5)). **c** Quantification of the relative ex vivo signal intensities in each organ at the time of animal sacrifice (*n* = 3 identically processed mice). The signal intensity from each reporter channel was normalized to the value measured from the intestines of each animal, which had high burdens of micrometastatic tumor foci. **d** Changes in the body weights as compared to baseline. **e** A plot of tumor burden over time as determined by changes from the baseline radiant flux associated with the BLI signal intensity. **f** Survival of LUC^+^ PDX-bearing mice that received ×4 weekly IP injections of CNP(MMAE) (at an equivalent dose of either 1 or 3 mg/kg MMAE), free cisplatin (at 3.5 mg/kg platinum), or PBS (control treatment); note that the black arrows indicate the timing of each dose of treatment. Mice that were administered CNP(MMAE) at either 1- or 3-mg/kg dose equivalent of free MMAE demonstrated significant improvements in survival as compared to mice treated with PBS or cisplatin (***p-value < 0.001, Log-rank test)

We studied the effects of CNP(MMAE) against primary cells obtained from the ascites of a patient with platinum-resistant HGSOC. Flow cytometry of primary cells in suspended culture confirmed their time-dependent uptake of dual-fluorophore-labeled, coated, and MMAE-containing NPs (5′-FAM-CNP(MMAE/Cy5.5)) (Supplementary Fig. 35A). CNP(MMAE) and free MMAE displayed comparable cellular cytotoxicites when examined at equivalent doses of MMAE (Fig. 6a); both agents were ~10,000× more potent than cisplatin at comparable levels of active drug (IC_50_ values in the single nM range for all MMAE-based formulations vs. ~20 μM for cisplatin). Comparisons of the levels of apoptosis induced by CNP(MMAE), NP(MMAE), and the free toxin (MMAE) confirmed the equivalent activities of all three MMAE formulations, which promoted apoptosis in >90% of the primary cells after 48 h of incubation (Supplementary Fig. 35B).

Analogous to the results seen in the disseminated cell-line xenograft model, IP administration of CNP(MMAE/Cy7.5) in a disseminated LUC^+^ PDX model of advanced-stage and platinum-resistant HGSOC, which was established from the same primary cells, confirmed uptake of the NPs within in vivo tumor locations (Fig. 6b and Supplementary Fig. 36A). Ex vivo enumeration of luminescent and fluorescent signal intensities again confirmed co-localization of CNP(MMAE/Cy7.5) within tumor implants on the serosal surfaces of excised organs (Fig. 6c and Supplementary Fig. 36B). Given the profound in vitro antiproliferative activity and the effective tumor accumulation afforded by IP administration of CNP(MMAE) within the PDX model, therapeutic testing commenced at the previously-examined dose level (i.e., 3 mg/kg equivalent of free MMAE) and at a reduced level (1 mg/kg equivalent of free MMAE). Separate mice were administered either PBS (control) or standard first-line therapy (cisplatin) at its known MTD (3.5 mg/kg based on platinum^42^) for comparison. LUC^+^ PDX tumor-bearing mice exhibited negligible weight loss when treated with four weekly administrations of either dose of CNP(MMAE) and when compared to controls (PBS and cisplatin; Fig. 6d). The reduced dose of CNP(MMAE) (i.e., 1 mg/kg equivalent of free MMAE) provided identical results with respect both to inhibition in the rate of tumor growth (Fig. 6e, Supplementary Fig. 37A–B) and to prolongation of animal survival (Fig. 6f) as compared to the higher dose level (i.e., 3 mg/kg equivalent of free MMAE). While cisplatin afforded comparable results to PBS, CNP(MMAE) was able to double the durations of both tumor growth inhibition and overall survival in this highly aggressive, platinum-resistant, and advanced-stage model of HGSOC. As supported by the sizes of their tumor masses at the time of demise (Supplementary Fig. 37C), all animals exhibited comparable tissue histological findings that were attributable to the effects of their advanced-stage cancers rather than to de novo material toxicities imparted by CNP(MMAE) (Supplementary Fig. 38).

## Discussion

While “platinum-resistant” epithelial ovarian cancers have proven refractory to most existing therapeutic strategies, we demonstrate that IP delivery of NP-toxin conjugates enables marked tumor accumulation in both disseminated cell-line xenograft and PDX models of HGSOC. Importantly, NP-toxin conjugates have no adverse effects on healthy tissues when introduced via this route of administration, presumably due to their preferential accumulation within peritoneal tumor deposits. Moreover, NP-based delivery obviates the substantial costs and time associated with ADC development and circumvents potential immunological toxicities imparted by antibody infusion. It should be noted that when testing experimental anticancer agents, most investigators employ subcutaneous cell-line xenografts or PDX tumors that are implanted on the flanks of mice. These murine models exhibit significant tumor neovascularization that aids in therapeutic delivery. The peritoneal tumor models adopted in our study more closely resemble the patterns of dissemination and neovascularization seen with human HGSOCs. In these aggressive and advanced-stage model systems, IP administration of untargeted and MMAE-conjugated NPs result in significant tumor growth inhibition and substantially increase overall survival. As in vivo microtubule inhibition may result in tumoristatic as opposed to purely tumoricidal effects, the tumors of mice treated in this current study did experience regrowth, albeit, after prolonged durations of time. Importantly, even in a highly resistant PDX model, our NP-toxin conjugate doubled the duration of activity seen with standard first-line chemotherapy, validating our therapeutic approach.

Given the success of these initial endeavors, NP conjugates of highly potent toxins may usher in a new class of anticancer agents formed from a modular and adaptable design strategy. With the enhanced safety and the marked antitumor efficacy seen after IP administration of our MMAE-conjugated NPs, they may find broader utility in the treatment of other tumors that spread by peritoneal dissemination, including advanced-stage gastrointestinal, genitourinary, and gynecologic malignancies. Additional cycles of dosing may increase the durations of tumor growth inhibition and may further improve survival outcomes. Our current experimental construct will need to be studied in multiple PDX models with varied genetic backgrounds and at different dose levels to better ascertain its therapeutic index and the spectrum of activities that may be expected in clinical populations. Future formulations may incorporate even more potent agents, additional therapies with complementary mechanisms of action, and/or imaging agents onto a single NP^31^. For targeting of other cancers, alternative linker chemistries will be examined, depending on the tumor type; additional modes of therapeutic administration may also need to be optimized^43^. We envision that NP conjugates of highly potent toxins will serve as a valuable addition to the clinical armamentarium, helping to realize the long-sought goal of retiring the use of non-targeted cytotoxic chemotherapy for the treatment of solid tumor and hematologic malignancies.

## Methods

### Reagents

Hydrogen disulfide, 4-nitrophenyl chloroformate, N-hydroxysuccinimide (NHS), 1-(3-dimethylaminopropyl)-3-ethylcarbodiimide hydrochloride (EDC·HCl), succinic anhydride, and hydroxybenzotriazole were purchased from Sigma-Aldrich (MA, USA). MMAE was purchased from Jiangyin Concortis Biotechnology Co., Ltd. (Jiangsu, China). N^3^-carbobenzyloxy-L-lysine (Z-Lys), β-benzyl L-aspartate (Bzl-Asp), and triphosgene were purchased from Shanghai Gilbiochem Co. (Shanghai, China). N-carboxy anhydride (NCAs) of Z-Lys and Bzl-Asp were synthesized, following previously reported procedures^44,45^. Amino-terminated methoxy-poly(ethylene glycol) (mPEG(5 KDa)-NH_2_) was purchased from Laysanbio, Inc. Methoxy-poly(ethyl glycol)-*block*-poly(L-glutamic acid) (mPEG_114_-*b*-PGA_30_) was synthesized, following previously reported methods^26^. Protocols for generating the intermediates (Compounds 1 and 2; Supplementary Figs. 1 and 2, respectively) and the final MMAE-bound linker (i.e., Compound 3; MMAE-prodrug; Supplementary Fig. 3), the mPEG_114_-*b*-PZLL_6_-*b*-PASP(DET)_30_ polymer (Supplementary Fig. 12–13), and the MMAE-conjugated polymer (i.e., mPEG_114_-*b*-PZLL_6_-*b*-PASP(DET)_30_—MMAE_2_; MMAE-P; Supplementary Fig. 14) are described in detail in the Supplementary Methods in the Supporting Information. All reactions were performed under N_2_. Unless otherwise stated, solvents were of HPLC quality and were purchased from Sigma-Aldrich (MA, USA); all chemicals were of analytical grade and were used without further purification.

### Cell culture

Established ovarian cancer cell lines (A2780, COV318, COV362, OVCAR4, OVCAR8, JHSO2, and SKOV3) were obtained from ATCC and cultured at 37 °C, 5% CO_2_ in RPMI 1640, which was supplemented with 10% fetal bovine serum (FBS, Gibco) and 1% penicillin/streptomycin (Corning, USA). Obtained from the laboratory of Dr. David Pepin under an IRB-approved protocol (MGH, 2007P001918), primary cells (ptAM-sph) were collected from the ascites of a patient with advanced-stage and “platinum-resistant” HGSOC and were utilized to generate a primary cell line^46^. The primary cell line was maintained in RPMI 1640 supplemented with 1% MEM-NEAA (Life technologies, USA), 2% B-27 (Gibco, USA), 1% insulin-transferrin-selenium (Gibco, USA), and 1% penicillin/streptomycin (Corning, USA). All cells lines were tested for mycoplasma, using the MycoAlert Mycoplasma Testing Kit (Lonza).

### Formation and characterization of the MMAE-prodrug and the unmodified, MMAE-conjugated and mPEG-b-PGA-coated nanoparticles

See Supplementary Methods in the Supporting Information.

### shRNA screen

An in vitro RNAi signature assay that employs murine lymphoma cells that are partially infected with one of eight different GFP-tagged shRNAs, which target genes related to p53 activation and cell death, was used to study the mechanism(s) of action of NP(MMAE), following previously established procedures^47^. In brief, partial populations of shRNA-treated cells were dosed with NP(MMAE) or free MMAE at their LD_80–90_ (i.e., the doses required to kill 80–90% of the cells) for 48 h. The relative enrichment or depletion imparted by each of the eight different shRNAs in response to the agents was used to provide a unique “signature” that accurately classified each species by its mechanism of action, when compared to an established reference set derived from drugs with known mechanisms of action ^31–34^.

### Intracellular uptake and the in vitro activity of unmodified, MMAE-conjugated and mPEG-b-PGA-coated nanoparticles

See Supplementary Methods in the Supporting Information.

### Animal handling

Female BALB/c (non-tumor bearing) mice at 4–6 weeks of age (Taconic, USA) were utilized for toxicity experiments (at MIT). Female NCR nu/nu mice at 5 weeks of age were used for pharmacology and efficacy experiments upon establishment of a disseminated cell-line xenograft model of HGSOC (8 × 10^5^ LUC^+^/RFP^+^ OVCAR8 cells/animal; 0.5 mL; IP injection) and were similarly purchased from Taconic. C.B-17/Icr-SCID/Sed mice were purchased from Charles River and bred at MGH; they were implanted with primary cells obtained from a patient with “platinum-resistant” and advanced-stage HGSOC after lentiviral transduction of firefly luciferase, establishing LUC^+^ PDX tumors (10 million cells/animal; 0.5 mL PBS; IP injection); assessments of CNP(MMAE) pharmacology and efficacy were performed in these PDX models. Tumor growth was monitored by BLI. All animal studies were performed under protocols approved by the MIT CAC (0615-069-1) and by the Massachusetts General Hospital IACUC (2009N000117).

### Toxicity study

Female BALB/c mice were administered one of the following treatments by IP injection: PBS (control), free MMAE (MMAE; at either 0.25 or 0.5 mg/kg), or coated and MMAE-conjugated NPs (CNP(MMAE)), at either 1- or 3-mg/kg dose equivalent of free MMAE). The body weights of the animals were monitored every other day starting with the first day of treatment administration (Day 0). At the end of the study, mice were sacrificed and blood was collected via cardiac puncture for serum chemistries and for complete blood counts. The major tissues and organs from each animal were also collected, fixed with 4% formalin, and stained with H&E.

### Biodistribution study

Tumors cells (0.8 million LUC^+^/RFP^+^ OVCAR8 cells/mouse or 4 million LUC^+^ primary HGSOC cells/mouse) were introduced into mice via IP injection and were allowed to grow until the LUC signals from their tumors reached 1 × 10^7^ radians (photons/sec/cm^2^/surface area; ~3 weeks for OVCAR8 and 2 weeks for PDX tumors). Thereafter, the animals were administered Cy7.5-conjugated CNP(MMAE) (CNP(MMAE/Cy7.5), at 3 mg/kg equivalent dose of free MMAE) by IP injection. After 24 h, bioluminescence (LUC) and fluorescence imaging (RFP and/or Cy7.5) proceeded using an IVIS Caliper LS system (auto exposition mode; Preseton Brook Runcorn, UK). The relative location of the tumors was visualized via in vivo imaging of RFP (*λ*_ex_ = 540 nm; *λ*_em_ = 580 nm) and/or LUC signals upon injection of d-luciferin (50 mg/kg). The in vivo biodistribution of CNP(MMAE/Cy7.5) was observed by gating on the Cy7.5 channel (*λ*_ex_ = 740 nm; *λ*_em_ = 820 nm). Upon completion of in vivo imaging, the mice were sacrificed and their organs were harvested and imaged ex vivo using the same parameters. The average photon flux in radians for the different reporter signals in each excised organ were quantified by gating on regions of interest, using Living Image Software V.4.5.2, for three separate mice per tumor type that were similarly processed. The relative signal intensity distribution in each organ (after normalization to the signal intensity recorded from the intestines, which was the major organ from which tumor deposits were explanted) was determined. Note that even after resection of peritoneal implants from the serosal surfaces of the intestines of each mouse, a residual signal remained that was attributed to the presence of microscopically infiltrating tumor cells.

### Pharmacodynamics study

Once the BLI radiant efficiency of their LUC^+^/RFP^+^ OVCAR8 tumors reached 1 × 10^7^ radians (photons/s/cm^2^/surface area; ~ 3 weeks), mice were administered ×2 weekly doses of one of the following treatments by IP injection: PBS, free MMAE (MMAE; 0.25 mg/kg), or coated and MMAE-conjugated NPs (CNP(MMAE); 3 mg/kg equivalent of free MMAE). Seventy-two hours after receiving the second dose, the mice were sacrificed and their tumors were harvested and fixed with 10% formaldehyde. The fixed tumors were sectioned for confocal imaging after IF staining with DAPI (nucleus) and FITC-labeled monoclonal anti-α-tubulin antibodies (intratumoral tubulin expression). The tumor slides were imaged at ×20 magnification with a FV1100 confocal microscope imaging system (Olympus, Tokyo, Japan).

### Efficacy study

Tumors cells (0.8 million LUC^+^/RFP^+^ OVCAR8 cells/mouse or 4 million LUC^+^ primary HGSOC cells/mouse) were introduced into mice via IP injection and were allowed to grow until the LUC signals from their tumors reached 1 × 10^7^ radians (photons/sec/cm^2^/surface area; ~3 weeks for OVCAR8 and 2 weeks for PDX tumors). Thereafter, the mice were administered free MMAE (0.25 mg/kg), CNP(MMAE) (at either 1- or 3-mg/kg dose equivalent of free MMAE), cisplatin at its MTD (3.5 mg/kg equivalent of platinum), or PBS by once weekly IP injection (on days 0, 7, 14, and 21). LUC signals emanating from the tumors of the animals were imaged periodically until the animals showed gross signs of toxicity or a loss of 15% in body weight. Changes in signal intensities were compared to baseline, were enumerated by gating on the whole peritoneal cavity (i.e., the area of tumor growth), and were determined by measuring the average photon flux in radians, which enabled normalization for differences in imaging areas between mice and in the same mouse over time. The average and distribution of the weights of the tumors collected from animals in each treatment group were also recorded at the time of sacrifice.

### Statistical methods

Statistical analyses were performed with GraphPad (GraphPad Prism 7). The differences between groups were evaluated by the two-tailed unpaired *t*-test; in vivo survival studies were compared by the Kaplan–Meier test.

### Data availability

The data that support the findings of this study are available from the corresponding author upon reasonable request.

## Electronic supplementary material

Supplementary Information
