# Publisher Correction: Nanoparticle conjugates of a highly potent toxin enhance safety and circumvent platinum resistance in ovarian cancer

**DOI:** 10.1038/s41467-018-02963-0

**Published:** 2018-02-07

**Authors:** Ruogu Qi, Yongheng Wang, Peter M. Bruno, Haihua Xiao, Yingjie Yu, Ting Li, Sam Lauffer, Wei Wei, Qixian Chen, Xiang Kang, Haiqin Song, Xi Yang, Xing Huang, Alexandre Detappe, Ursula Matulonis, David Pepin, Michael T. Hemann, Michael J. Birrer, P. Peter Ghoroghchian

**Affiliations:** 1 Koch Institute for Integrative Cancer Research at MIT, 500 Main Street, Cambridge, MA 02139 USA; 2 Massachusetts General Hospital, 55 Fruit Street, Boston, MA 02114 USA; 3 Dana Farber Cancer Institute, 450 Brookline Avenue, Boston, MA 02215 USA; 4 Harvard Medical School, 25 Shattuck Street, Boston, MA 02115 USA

Correction to: *Nature Communications* 10.1038/s41467-017-02390-7, published online 18 December 2017.

The original version of this Article contained an error in the spelling of the author Yingjie Yu, which was incorrectly given as Yu Yingjie. Furthermore, in Figure 3a, the labels ‘MD | *p* < 0.05’ incorrectly read ‘MD | *p* > 0.05’. These errors have now been corrected in both the PDF and HTML versions of the Article.

