# Author Correction: Nanoparticle conjugates of a highly potent toxin enhance safety and circumvent platinum resistance in ovarian cancer

**DOI:** 10.1038/s41467-020-14903-y

**Published:** 2020-04-17

**Authors:** Ruogu Qi, Yongheng Wang, Peter M. Bruno, Haihua Xiao, Yingjie Yu, Ting Li, Sam Lauffer, Wei Wei, Qixian Chen, Xiang Kang, Haiqin Song, Xi Yang, Xing Huang, Alexandre Detappe, Ursula Matulonis, David Pepin, Michael T. Hemann, Michael J. Birrer, P. Peter Ghoroghchian

**Affiliations:** 1 Koch Institute for Integrative Cancer Research at MIT, 500 Main Street, Cambridge, MA 02139 USA; 2 Massachusetts General Hospital, 55 Fruit Street, Boston, MA 02114 USA; 3 Dana Farber Cancer Institute, 450 Brookline Avenue, Boston, MA 02215 USA; 4 Harvard Medical School, 25 Shattuck Street, Boston, MA 02115 USA

**Keywords:** Nanoparticles, Nanoparticles, Chemotherapy, Chemotherapy, Ovarian cancer

Correction to: *Nature Communications* 10.1038/s41467-017-02390-7, published online 18 December 2017.

This Article contains an error in Fig. 4. In Fig. 4e, the image representing NP was taken from the PBS image. The correct version of Fig. 4 is:which replaces the previous incorrect version:The raw data for the images presented in Fig. 4 can be accessed using the Figshare link 10.6084/m9.figshare.11755248.v1. The error has been corrected in the PDF and HTML versions of the article.

