# Supplementary material for: Nanoparticle conjugates of a highly potent toxin enhance safety and circumvent platinum resistance in ovarian cancer
Source: Nat Commun. 2017 Dec 18;8:2166. doi: 10.1038/s41467-017-02390-7 (PMC5735131; doi:10.1038/s41467-017-02390-7)
Supplement: Supplementary file 1 — Supplementary Information [file 41467_2017_2390_MOESM1_ESM.pdf]

SUPPLEMENTARY METHODS

<u>*General Measurements:*</u> ^{1}H and ^{13}C NMR spectra were recorded at 400 and at 100 MHz, respectively, using a Bruker 400 AVANCE III instrument (Bruker Avance III NMR, Rheinstetten, Germany). Chemical shifts (δ) are reported in parts per million (ppm) from low to high field and referenced to residual solvent (i.e. the $CHCl_3$ singlet at 7.26 ppm, the DMSO quintet at 2.50 ppm, or the CH_2Cl_2 singlet at 5.30 ppm). ^{13}C-NMR spectra were referenced to the center line of the $CDCl_3$ triplet at 77.0 ppm or to that of the DMSO septet at 39.5 ppm. Standard abbreviations of multiplicity are indicated as follows: br = broad, s = singlet, d = doublet, t = triplet, m = multiplet.

Liquid chromatography–mass spectrometry (LC-MS) and preparative HPLC were performed on an Agilent 1260 LC system equipped with a Zorbax SB-C18 rapid resolution HT column (Agilent Technologies, Palo Alto, CA, USA). Solvent gradients consisted of mixtures of Milli-Q water with 0.1% acetic acid (AcOH) and HPLC-grade acetonitrile (ACN), beginning with H_2O:ACN = 90:10 v/v and reaching H_2O:ACN = 0:100 v/v within 8 min and at a flow rate of 0.5 mL/min (*Method 1*). Mass spectra were obtained using an Agilent 6130 single quadrupole mass spectrometer (Agilent Technologies). All chromatograms were recorded at 220 nm. Spontaneous reductive hydrolysis of the MMAE-bound linker (Compound 3; MMAE-prodrug) was also monitored by LC-MS, using same method.

Semi-preparative RP-HPLC was performed on the same Agilent 1260 LC system with a Zorbax SB-C18 semi-preparative column and at 5 mL/min, using a linear gradient beginning with water:ACN = 90:10 v/v and reaching water:ACN = 0:100 v/v within 15 min (*Method 2*). GPC measurements were performed on the Agilent 1260 LC system, using a Shodex KD-806M GPC column (Wyatt Technology, Wellensieck, Minden, Germany) at 60 °C and at a flow rate of 1 mL/min. Filtering *N*,*N*-Dimethylformamide (DMF, Sigma-Aldrich), containing 0.2 M LiBr, was used

as the elution phase. A refractive index detector (T-REX, Wyatt Technology) was used for copolymer analysis.

For NP characterization studies, DLS and zeta potential measurements were conducted at room temperature, using a ZS90 Malvern Nanosizer (Malevern Instruments, UK) equipped with a He-Ne laser source (633 nm). NP samples were suspended in Milli-Q water at a concentration of ~1 mg/mL. At least 3 measurements were made per sample. TEM images were obtained with a JEOL-1100 Transmission Electron Microscope (JEOL Corporation, Tokyo, Japan) upon phosphate acetic negative staining (Nano Core facility of the David H. Koch Institute for Integrative Cancer Research at MIT (Cambridge, MA)).

Determination of the kinetics for the reaction of Compound 3 with GSH: An aqueous solution of Compound 3 (final 10 μM) was combined with glutathione (GSH, 5 mM in PBS, pH 7.4) under Ar and placed in a 37 °C oven. At specified time points, 10 μL of the reaction mixture were extracted and injected into an HPLC column in order to utilize LC-MS (*Method 1*) to observe the reaction kinetics and to identify the intermediate residues that were produced upon cleavage of Compound 3 to yield free MMAE.

Formation of MMAE-conjugated nanoparticles and determination of the kinetics of MMAE release in different in situ environments: Uncoated MMAE-conjugated nanoparticles (NP(MMAE)) were formed by spontaneous self-assembly upon dissolution of MMAE-P into phosphate buffer saline (PBS, pH 7.4). For measurements of the release kinetics of free MMAE from the MMAE-prodrug or from NP(MMAE), aliquots of PBS suspensions with or without glutathione (GSH; 5 mM) were taken at various time points after incubation at 37 °C and under Ar. At each time point, small volume aliquots (10 μL) were extracted and subject to LC-MS to assess for the presence of free MMAE and various reaction intermediates.

Synthesis of Cy5.5-labeled and MMAE-conjugated nanoparticles (NP(MMAE)/Cy5.5)): Cy5.5-NHS (Lumiprobe, FL, USA) in DMSO was added to an aqueous suspension of NP(MMAE). This solution was stirred for 24 h in the dark and the unreacted dye was removed by centrifiguation filtration (Centricon, Thermo-Fisher; 4000 rpm, cut-off 3 kDa). The fluorescently-labeled NPs (NP(MMAE/Cy5.5)) were then lyophilized for further reconstitution at a later time. For *in vivo* biodistribution studies, Cy7.5-NHS was similarly reacted with NP(MMAE) in lieu of Cy5.5-NHS.

Neutralization of the cationic charge of NP(MMAE) with mPEG-b-PGA, generating CNP(MMAE): mPEG-*b*-PGA was used to coat NP(MMAE) by layer-by-layer deposition in order to neutralize its positive surface charge. The mPEG-*b*-PLGA polymer was mixed with a preformed suspension of NP(MMAE) (in 10 mM HEPES) at different initial molar ratios of carboxyl groups (of the PGA that comprised the mPEG-*b*-PGA coating) to amino groups (of PASP that comprised the mPEG-*b*-PZLL-*b*-PASP(DET)-based NPs), which served to alter the negative to positive charge (N/P) ratio in solution. The diameters and the zeta potentials of the resultant CNP(MMAE) formulations were measured using a Zetasizer (Malvern Instruments). Centrifugation filtration (Millipore; 100 kDa cutoff membrane; 3000 rpm for 5 min) was used to remove any uncomplexed mPEG-*b*-PGA from the CNP(MMAE) suspensions. The final diameters and zeta potentials of these coated formulations were subsequently measured. Conjugation of 5'-FAM to mPEG-*b*-PGA via EDC/NHS chemistry prior to complexation with Cy5.5-labeled NP(MMAE) (*vide supra*) generated dual-labeled, coated, and MMAE-conjugated NPs (5'-FAM-CNP(MMAE/Cy5.5)) that enabled independent tracking of each species during confocal microscopy and flow cytometry experiments (*vide infra*).

Stability of uncoated and coated MMAE-conjugated nanoparticles in different in situ environments: NP(MMAE) and CNP(MMAE) were prepared in PBS buffer, which was subsequently exchanged with solutions that varied in ionic strength and/or pH in order to assess

for particle stability in different environments and over time (e.g. 10 mM HEPES, pH 7.4; 10 mM HEPES with 150 mM NaCl, pH 7.4; or, sodium acetate, pH 5.0). The sizes (by DLS) and surface charges (as determined by zeta potential measurements) of the formulations were assessed from suspension aliquots in 24 h intervals and by using the Zetasizer instrument.

Intracellular uptake of fluorescently-labeled and MMAE-conjugated nanoparticles into ovarian cancer cells: OVCAR8 cells were plated onto coverslips in 6-well plates (1×10^5 cells/well) and cultured with RPMI 1640 supplemented with 10% FBS for 24 h. The cells were then incubated with NP(MMAE/Cy5.5) or CNP(MMAE/Cy5.5) at a fixed concentration of Cy5.5 (50 nM) for either 30 min or 2 h, washed twice with cold PBS (pH 7.4, 0.01 M), and fixed with 4% formaldehyde (Sigma-Aldrich, St. Louis, USA). To label the cell nuclei, samples were incubated with DAPI (1 mg/mL; Sigma-Aldrich, St. Louis, USA) for 15 min in PBS. The F-actin of the cells was stained with Alexa488-phalloidin (Thermo Fisher Scientific, MA, USA) in PBS. The coverslips were mounted on slides and the intracellular distributions of NPs with respect to F-actin and cellular nucleic were visualized, using an Olympus FV1100 confocal laser scanning fluorescence microscope (Olympus, Tokyo, Japan).

For flow cytometry measurements, OVCAR8 cells were cultured in 12-well plates (3×10^4 cells/well) prior to incubation with NP(MMAE/Cy5.5) or CNP(MMAE/Cy5.5) at a fixed concentration of Cy5.5 (50 nM) for either 30 min or 2 h. The cells were then washed twice with cold PBS, lysed with trypsin–EDTA solution, collected by centrifugation (1500 rpm for 5 min), and analyzed for fluorescence intensity, using a FACSCalibur flow cytometer (BD Biosciences, USA). Cells with a fluorescent signal intensity above the threshold value for untreated cells (i.e. blank) were quantified. All data were analyzed using FlowJo software (Version 7.6.2).

Cellular viability screens to determine the activity of MMAE-conjugated nanoparticles against established ovarian cancer cell lines and primary "platinum-resistant" HGSOC cells: A2780, COV318, COV362, OVCAR4, OVCAR8, and SKOV3 cells were seeded in 96-well plates (5,000 cells/well) and allowed to adhere overnight; the free drug formulation of MMAE (MMAE), NP(MMAE), or CNP(MMAE) were then incubated with the cells at different concentrations; and, relative cellular viability was assessed after 72 h using the calorimetric MTT assay and in comparison to untreated cells. Primary cells were similarly placed in suspended cultures at a density of 10,000 cells/mL; the same treatments were added to equivalent final concentrations of MMAE; they were allowed to incubate with the cells for 72 h; and, the CCK8 colorimetric assay was used to determine relative viability as a function of treatment administration.

Colony formation assay: OVCAR8 cells (1×10^3 cells/well) were treated with the free drug formulation of MMAE (MMAE), NP(MMAE), or CNP(MMAE) for 7 days and at a fixed MMAE concentration (5 nM); PBS, empty uncoated NPs (NP) and empty coated NPs (CNP) served as control treatments and were incubated with the cells at equal volumes and/or polymer concentrations. Following the 7 days of incubation with each experimental group, tumor cell colonies were fixed and stained with 0.5% gentian violet PBS solution and photographs were taken under illumination. The numbers of confluent colonies were ennumerated, using ImageJ software.

Determination of cellular apoptosis by flow cytometry: OVCAR8 cells and primary cells from a patient with platinum-resistant HGSOC were seeded in 6-well plates (5×10^5 cells/well) and allowed to adhere/equilibrate overnight. The cells were then treated with the free drug formulation of MMAE (MMAE), NP(MMAE), or CNP(MMAE) for 48 h and at a fixed MMAE concentration (5 nM). PBS, empty uncoated NPs (NP) and empty coated NPs (CNP) served as controls and were

incubated with the cells at equal volumes and/or polymer concentrations. Following the 48 h of incubation with each treatment group, the fractions of apoptotic cells were detected by flow cytometry, using the Annexin V-FITC Apoptosis Detection Kit I.

Cell cycle distribution of established ovarian cancer cells lines treated with MMAE-conjugated nanoparticles as assessed by flow cytometry: OVCAR8 cells (5×10^4 cells/well) were treated with the free drug formulation of MMAE (MMAE), NP(MMAE), or CNP(MMAE) for 48 h and at a fixed MMAE concentration (0.5 nM). PBS, empty uncoated NPs (NP) and empty coated NPs (CNP) served as control treatments and were incubated with the cells at equal volumes and/or polymer concentrations. Following 48 h of incubation with each treatment group, the cells were fixed and stained with PI. Their DNA content was measured, using a FACSCalibur flow cytometry (BD Biosciences, San Jose, CA, USA); and, cell cycle distribution was determined, using FlowJo software (Version 7.6.2).

In vitro immunofluorescence staining: OVCAR8 cells were seeded in 6-well plates (5×10^4 cells/well) on top of coverslips and allowed to adhere overnight. The cells were then treated with the free drug formulation of MMAE (MMAE), NP(MMAE), or CNP(MMAE) for 48 h and at a fixed MMAE concentration (5 nM). PBS, empty uncoated NPs (NP) and empty coated NPs (CNP) served as control treatments and were incubated with the cells at equal volumes and/or polymer concentrations. Following the 48 h incubation with each treatment group, the cells were fixed in cold methanol (4 °C) for 30 min and then incubated for an additional 30 min with FITC-labeled anti-α-tubulin antibody (Sigma-Aldrich) at room temperature (1:100 v:v; λ_{ex} = 488; λ_{em} = 570 nm). DAPI was used to stain the cell nuclei. The coverslips were mounted on slides and the distributions of α-tubulin with respect to cellular nuclei were visualized using an Olympus FV1100 confocal laser scanning fluorescence microscope (Olympus, Tokyo, Japan).

Synthetic Procedures:

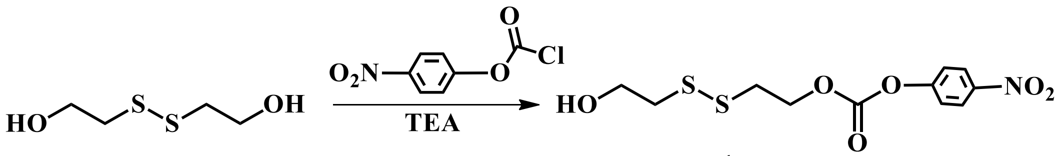

1

Supplementary Figure 1: Synthesis of Compound 1.

4-nitrophenyl chloroformate (131 mg, 0.65 mmol) was dissolved in THF (3 mL) and was combined with a stirred mixture of hydrogen disulfide (200 mg, 1.3 mmol) and pre-dried TEA (157 mg, 1.55 mmol) in anhydrous THF (5 mL) by drop-wise addition. The solution was subsequently stirred overnight and concentrated under reduced pressure. The crude product, comprising a yellowish material, was then purified by column chromatography over a silica gel, using a 20:1 v:v mixture of hexane:ethyl acetate as the eluent; the purified product consisted of a clear oil and was denoted as Compound 1 (150 mg, 72% yield). ^{1}H NMR (400 MHz, $CDCl_3$) δ 8.27 (d, 2H), 7.40 (d, 2H), 4.56 (t, 2H), 3.91 (t, 2H), 3.04 (t, 2H), 2.92 (t, 2H). ^{13}C NMR (101 MHz, $CDCl_3$) δ 155.38, 152.38, 145.51, 125.36, 121.79, 66.09, 60.29, 41.55, 36.51.

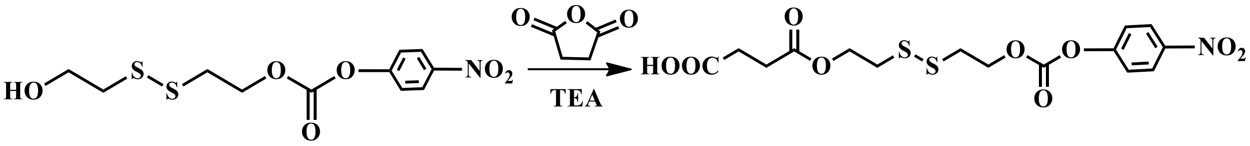

1

2

Supplementary Figure 2: Synthesis of Compound 2.

Succinic anhydride (60 mg; final 0.6 mmol/mL) was combined with a mixture of Compound 1 (100 mg, 0.3 mmol) and TEA (40 mg, 0.4 mmol) in THF (10 mL). The solution was stirred at room temperature for 24 h and then concentrated by rotary evaporation. The crude material was purified by column chromatography over a silica gel, using a 25:1 v:v mixture of dichloromethane:methanol as the eluent; the purified product consisted of a clear oil and was

denoted as Compound 2 (110 mg, 84% yield). ^{1}H NMR (400 MHz, DMSO-d_6) δ 12.22 (s, 1H), 8.31 (d, 2H), 7.56 (d, 2H), 4.50 (t, 2H), 4.28 (t, 2H), 3.11 (t, 2H), 2.99 (t, 2H), 2.50 (m, 4H, masked by DMSO). ^{13}C NMR (101 MHz, DMSO-d_6) δ 173.78, 172.51, 155.67, 152.34, 145.68, 125.89, 123.05, 67.11, 62.37, 55.37, 36.74, 36.50, 29.10.

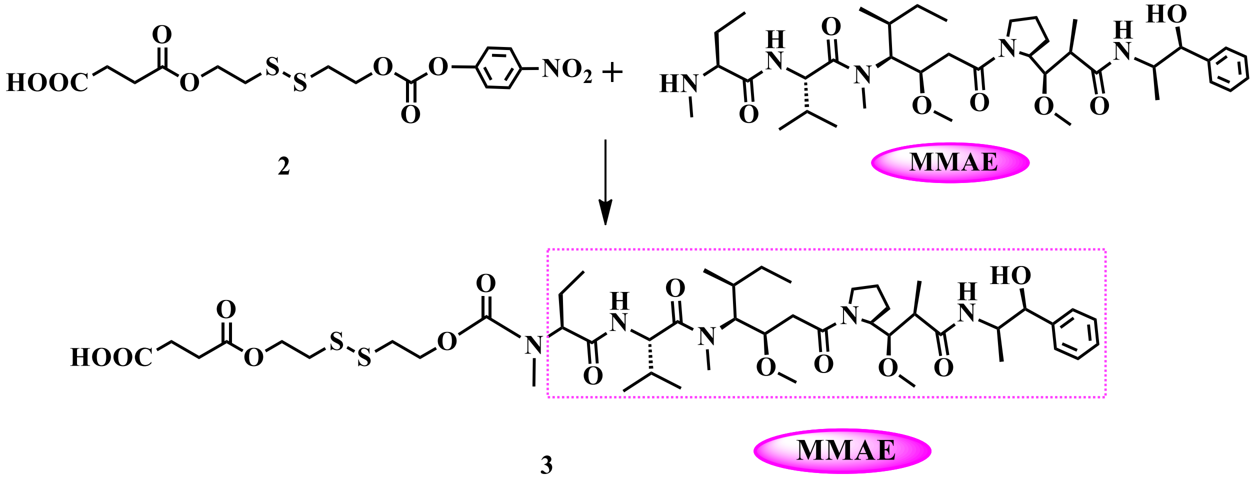

Supplementary Figure 3: **Synthesis of Compound 3.**

MMAE (55 mg, 0.8 equiv.) and hydroxybenzotriazole (HOBt) (2 mg, 0.013 mmol) were added to a stirred solution, consisting of Compound 2 (40 mg, 0. 14 mmol) in a mixture of DMF (0.3 mL) and pyridine (0.1 mL). The reaction was stirred for 24 h at room temperature and monitored by analytical HPLC. The solution was then concentrated under reduced pressure and the crude material was purified by semi-preparative RP-HPLC (using *Method 2*) to afford Compound 3 (49 mg, 64% yield). ^{1}H NMR (400 MHz, DMSO-d_6) δ 8.21 (s, 0.5H), 8.06 (br s, 0.5H), 7.91 (d, 0.5H), 7.62 (d, 0.5H), 7.30 (m, 4H), 7.17 (m, 1H), 5.4 (br s, 1H), 4.76 (m, 0.5H), 4.72 (m, 0.5H), 4.49 (m, 2H), 4.26 (m, 5.5H), 3.94 (m, 2H), 3.78 (m, 0.5H), 3.62-3.54 (m, 3H), 3.31-3.18 (m, 6.5H, masked by water), 3.03 (m, 6H), 2.97 (m, 3H), 2.82 (m, 0.5H), 2.51-2.46 (m, 5H, masked by DMSO), 2.30(m, 1H), 2.12 (br, 3H), 1.75 (m, 3H), 1.54(m, 2H), 1.32 (m, 1H), 1.00-0.76 (m, 25H). ^{13}C NMR (101 MHz, DMSO-d_6) δ173.84, 172.83, 172.75, 172,53, 169.19, 144.13, 128.25, 127.18, 126.91, 85.81, 82.10, 78.07, 77.31, 75.23, 63.88, 63.24, 62.38, 61.38, 60.73, 59.12, 58.64, 57.60, 54.57,

50.22, 49.62, 46.69, 44.21, 43.66, 36.82, 35.60, 31.98, 30.12, 29.20, 27.18, 25.81, 24.86, 23.58, 21.58, 19.30, 18.81,15.92, 15.38, 10.87, 10.68. MS (ESI) m/z (M+H) Calcd. For $C_{48}H_{79}N_5O_{13}S_2$: 997.5 Obsd.: 998.5 (M+H), 499.8(M+2H). The purity of the Compound 3 was characterized by analytic HPLC (using *Method 1*), confirming a purity of 98%.

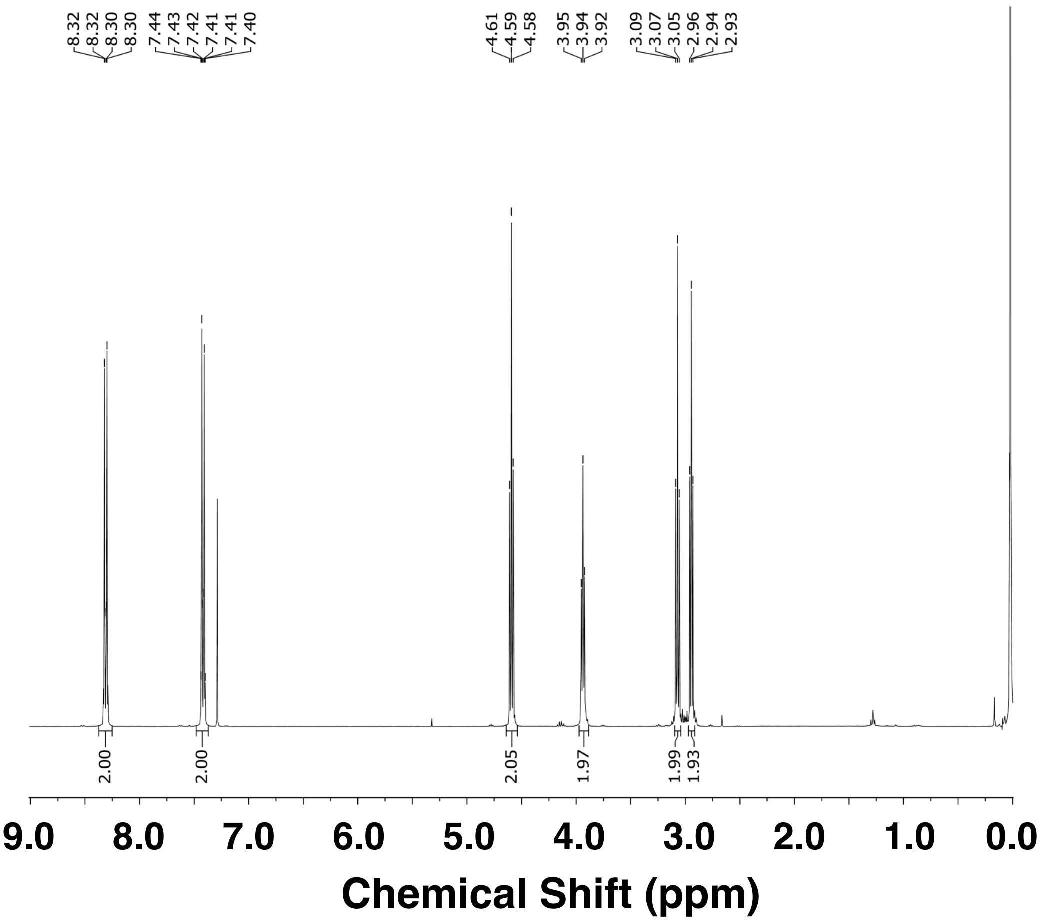

Supplementary Figure 4: ^{1}H NMR spectrum of Compound 1 ($CDCl_3$).

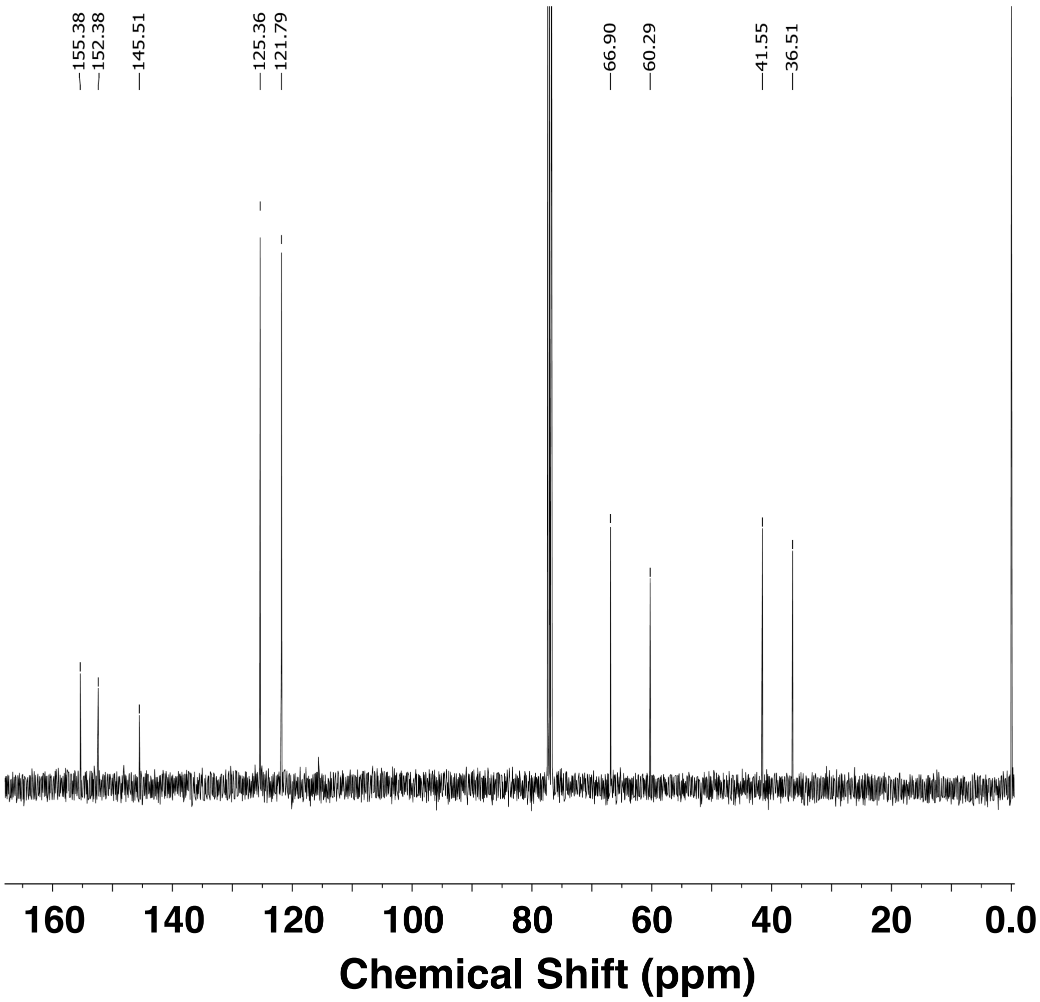

Supplementary Figure 5: ^{13}C NMR spectrum of Compound 1 ($CDCl_3$).

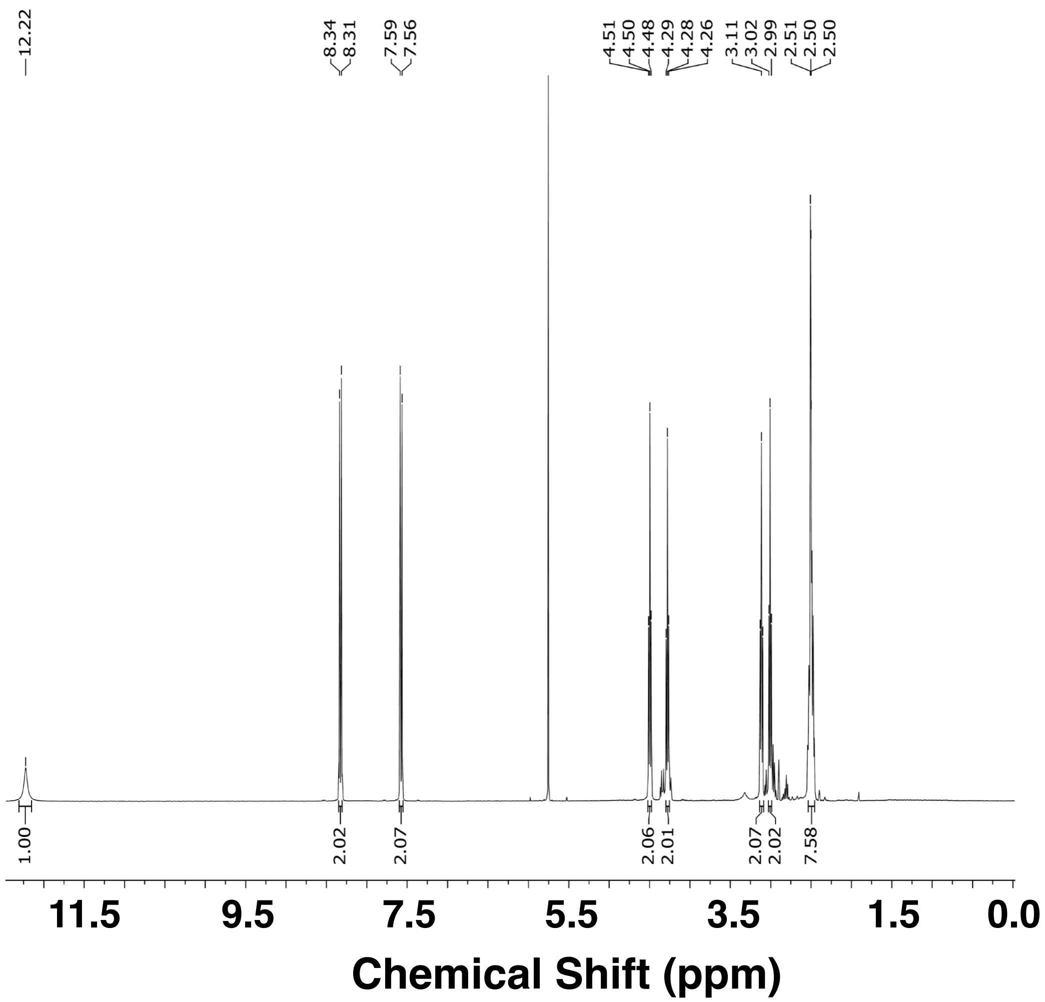

Supplementary Figure 6: ^{1}H NMR spectrum of Compound 2 (DMSO-d_6).

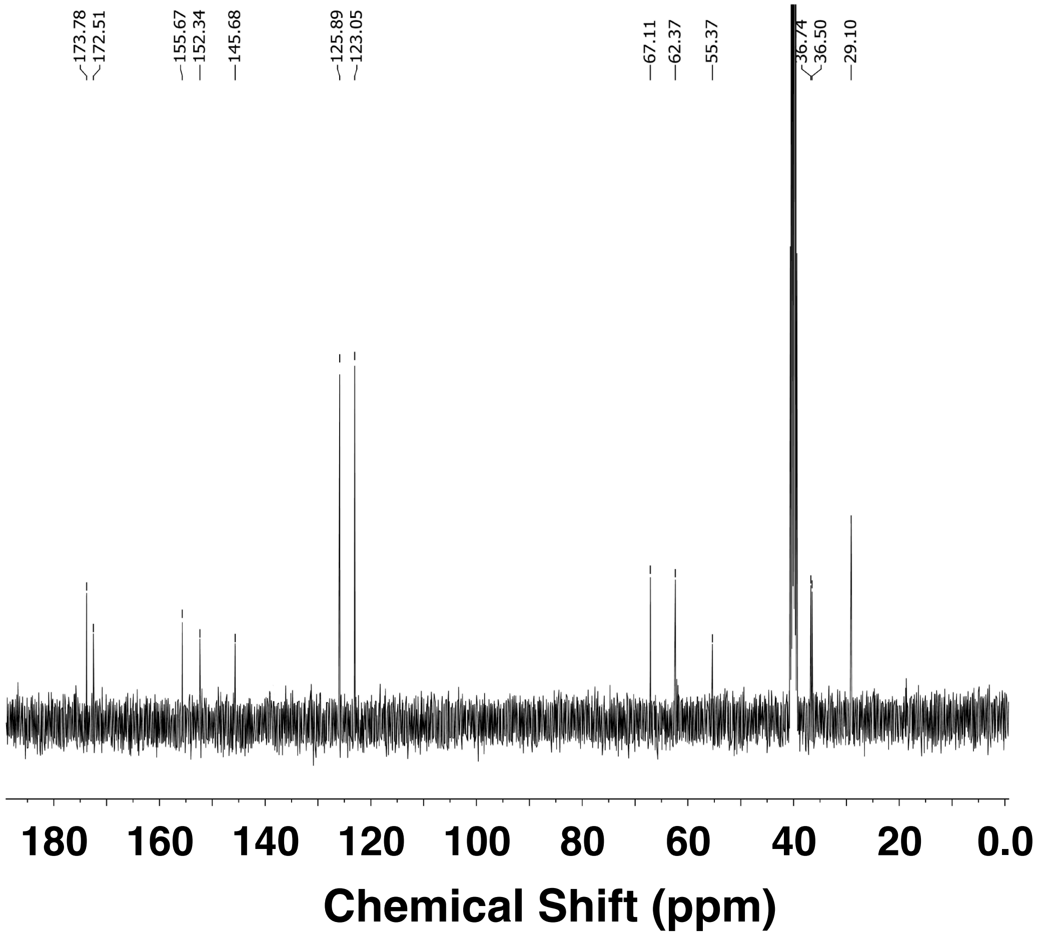

Supplementary Figure 7: ^{13}C NMR spectrum of Compound 2 (DMSO-d_6).

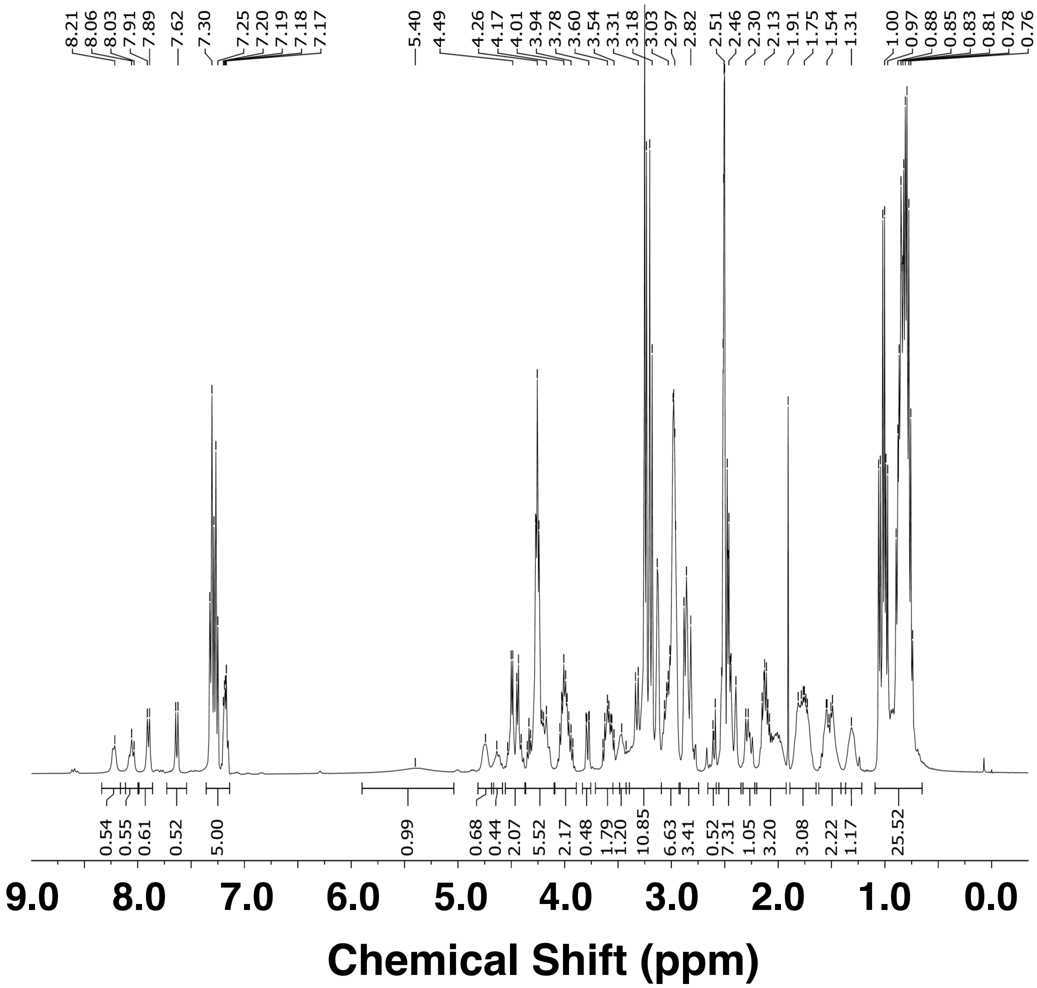

Supplementary Figure 8: ^{1}H NMR spectrum of Compound 3 (DMSO-d_6).

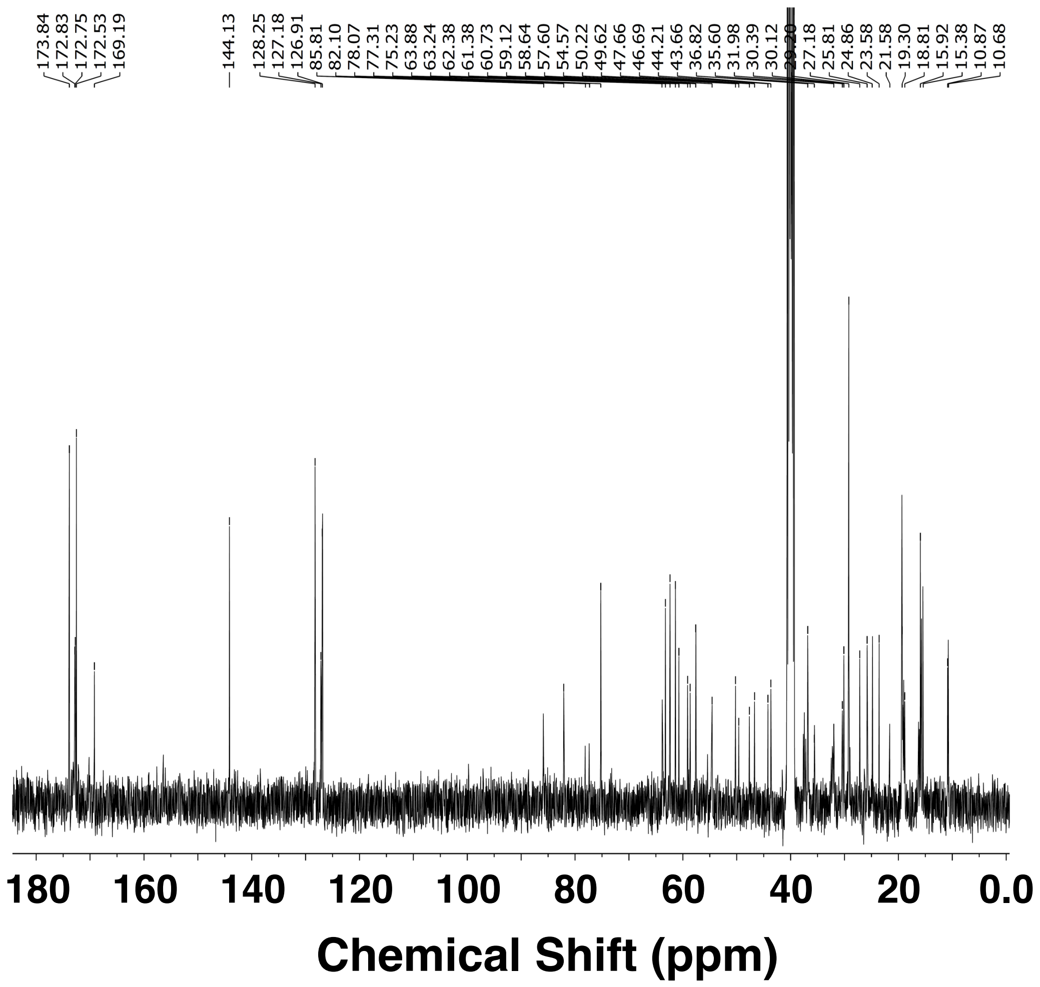

Supplementary Figure 9: ^{13}C NMR spectrum of Compound 3 (DMSO-d_6).

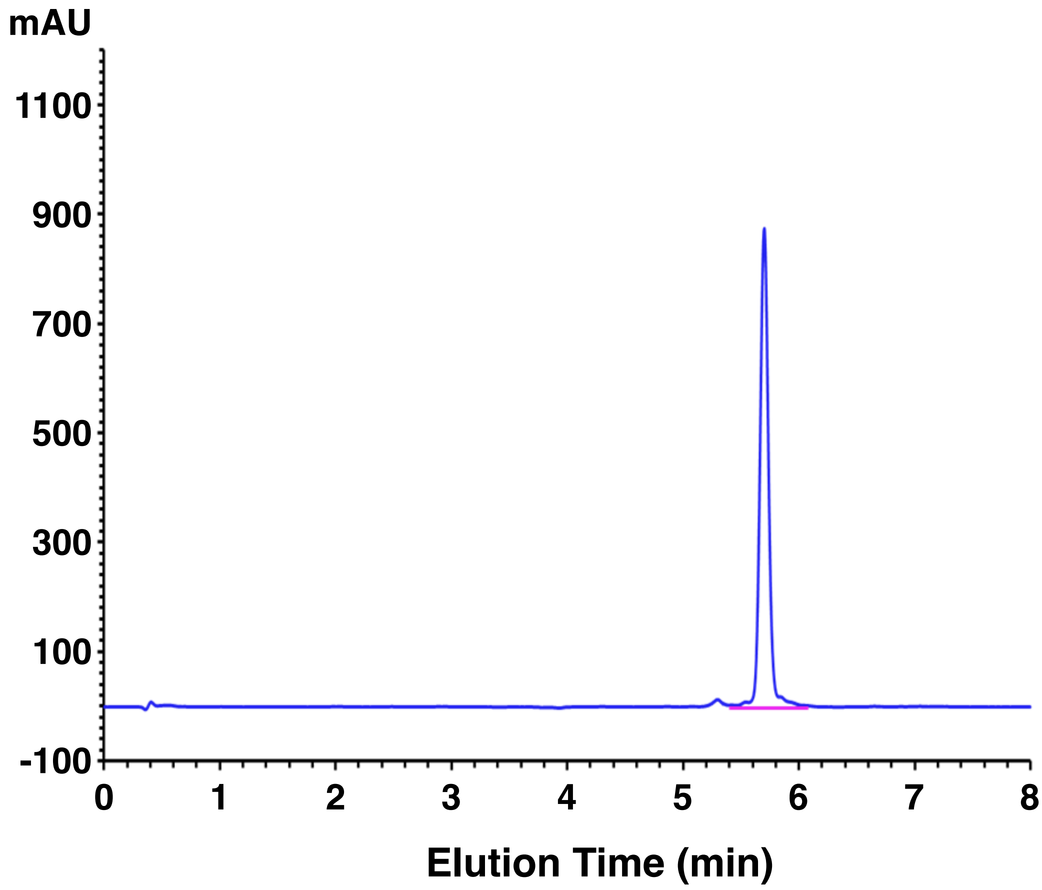

Supplementary Figure 10: HPLC spectrum of Compound 3. Solvent gradients consisted of mixtures of Milli-Q water with 0.1% acetic acid (AcOH) and acetonitrile (ACN), beginning with H_2O:ACN = 90:10 v/v and reaching H_2O:ACN = 0:100 v/v within 8 min; flow rate 0.5 mL/min (i.e. *Method 1*).

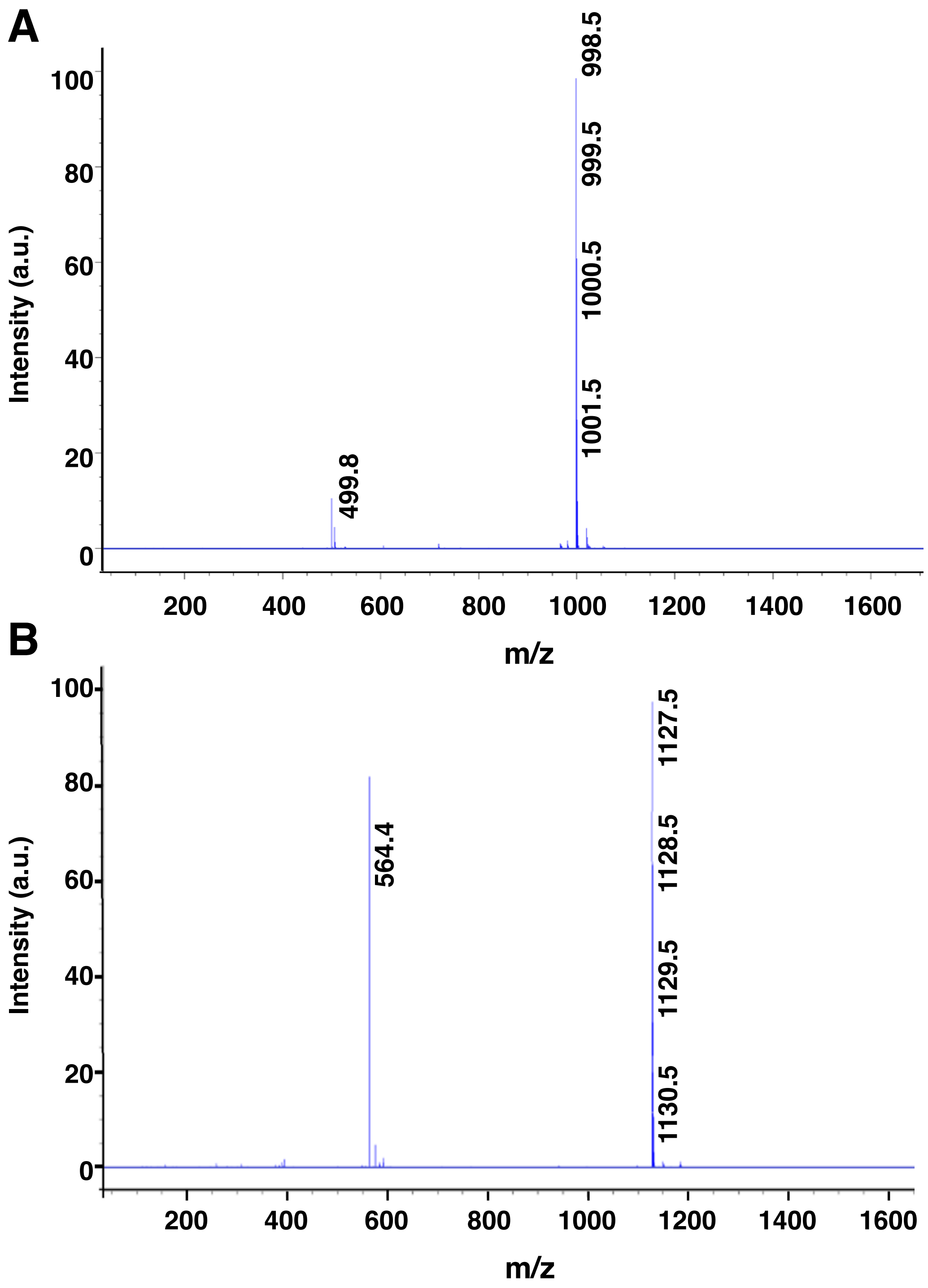
A
Intensity (a.u.)
100
80
60
40
20
0
998.5
999.5
1000.5
1001.5
499.8
200
400
600
800
1000
1200
1400
1600
m/z
B
Intensity (a.u.)
100
80
60
40
20
0
1127.5
1128.5
1129.5
1130.5
564.4
200
400
600
800
1000
1200
1400
1600
m/z

C

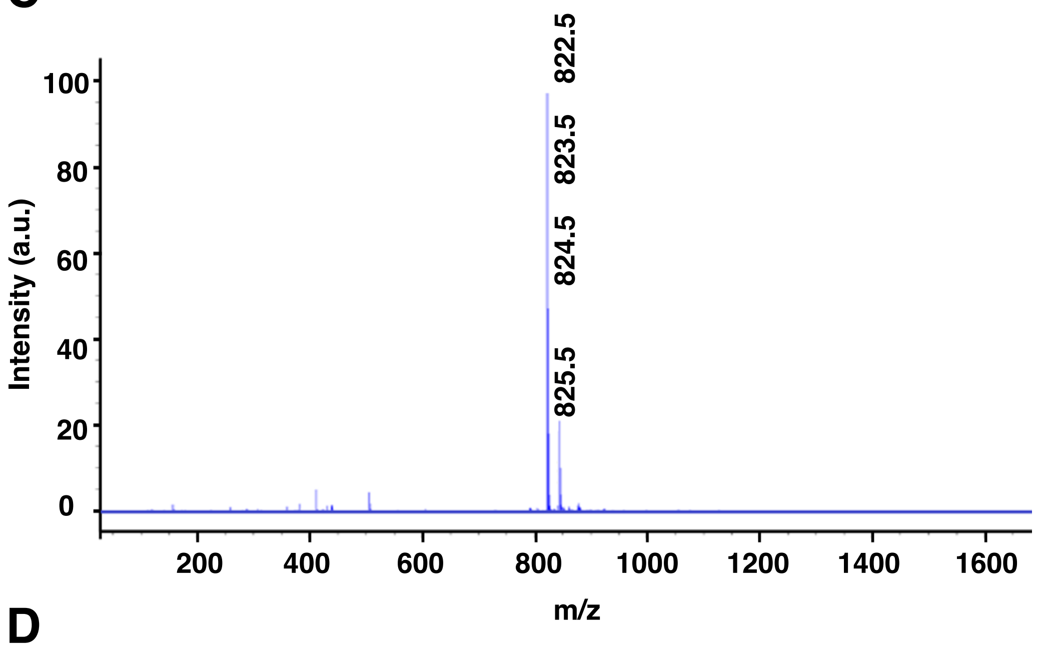
822.5
823.5
824.5
825.5
Intensity (a.u.)
100
80
60
40
20
0
200
400
600
800
1000
1200
1400
1600
m/z

D

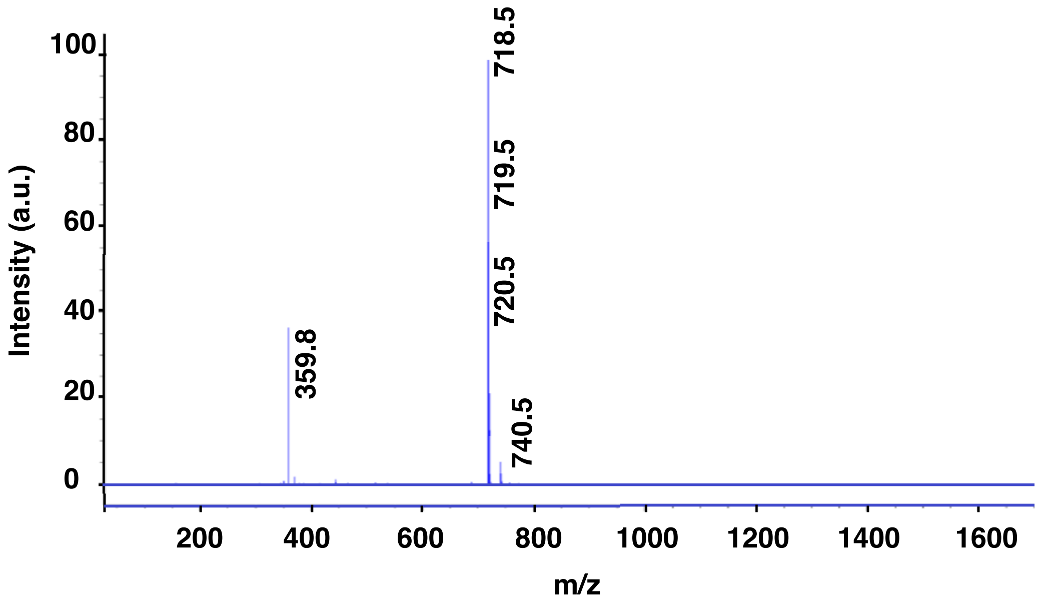
718.5
719.5
720.5
740.5
359.8
Intensity (a.u.)
100
80
60
40
20
0
200
400
600
800
1000
1200
1400
1600
m/z

E

Retention Time (min)	m/z	Chemical Structure and Identification	Calculated Mass (g/mol)
5.73	998.5 $(M+H)^+$<br>499.8 $(M+2H)^{2+}$	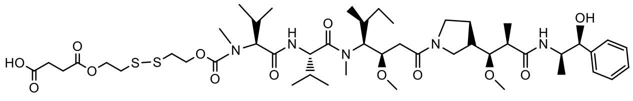<br>Compound 3	997.5
4.67	1127.5 $(M+H)^+$<br>564.4 $(M+2H)^{2+}$	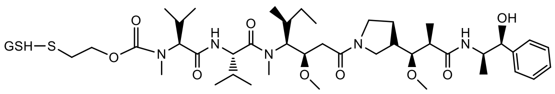<br>Compound 4	1126.5
5.63	822.5 $(M+H)^+$	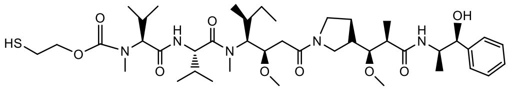<br>Compound 5	821.5
3.93	718.5 $(M+H)^+$<br>359.8 $(M+2H)^{2+}$<br>740.5 $(M+Na)^+$	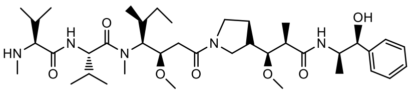<br>MMAE	717.5

Supplementary Figure 11: Mass spectra of various residues released from Compound 3 (MMAE-prodrug) upon reaction with glutathione. A solution of Compound 3 in PBS (final 10 μM) was combined with a solution of glutathione (GSH, 5 mM in PBS, pH 7.4) under Ar and incubated at 37 °C. LC-MS measurements (using *Method 1*) of aliquots extracted at various time points were performed in order to monitor for the presence of different reaction intermediates, which were identified from their positive mode mass spectra. The possible peak assignments for A) Compound 3, as well as for 3 additional compounds that were isolated by LC-MS: B) Compound 4, C) Compound 5, and D) free MMAE, are shown. E) Table summarizing the retention times for reaction products of Compound 3 with GSH and as recorded by HPLC with *Method 1*; the mass peaks of intermediate residues that were isolated, as well as the putative chemical structures, identifications, and calculated masses of their associated compounds, are reported.

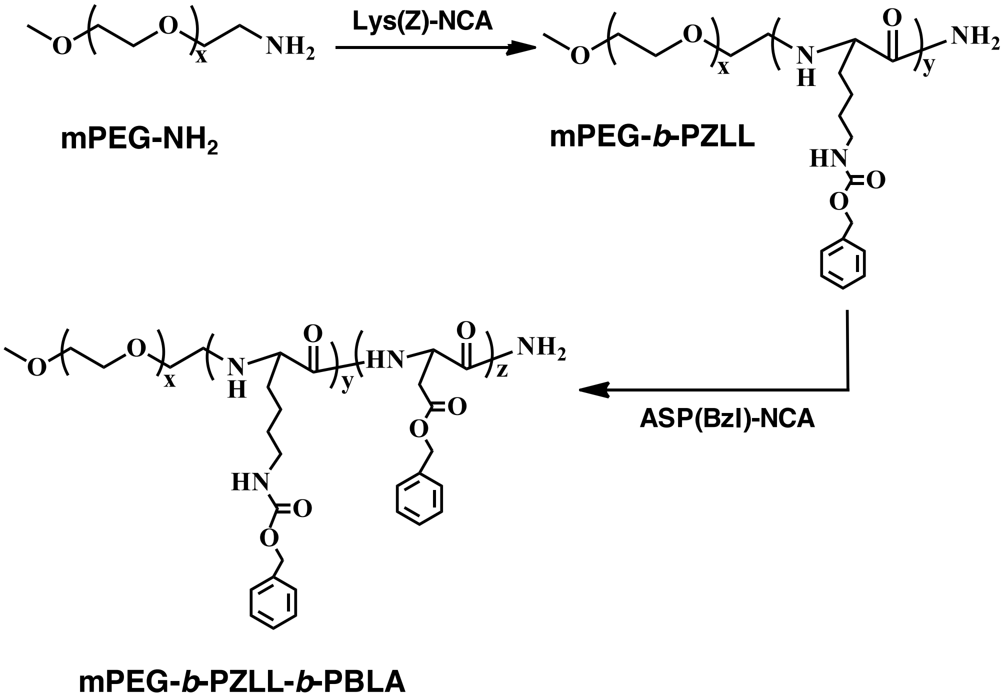

Supplementary Figure 12: **Synthesis of the triblock copolymer of mPEG-*b*-PZLL-*b*-PBLA.**

A diblock copolymer of methoxypoly(ethylene glycol)-*block*-poly(carbobenzyloxy-L-lysine) (mPEG-*b*-PZLL) and a triblock copolymer of methoxypoly(ethylene glycol)-*block*-poly(carbobenzyloxy-L-lysine)-poly(β-benzyl L-aspartate) (mPEG-*b*-PZLL-*b*-PBLA) were synthetized via ring-opening polymerization (ROP), using mPEG-NH_2 as the initiator, by combining N-epsilon-benzyloxycarbonyl-L-lysine-N-carboxy anhydride (Lys(Z)-NCA) and beta-benzyl-L-aspartic-N-carboxy anhydride (Asp(Bzl)-NCA) in DMF, and by modification of a previously reported method.[1] The polymerization ratio of mPEG-*b*-PZLL was calculated from its ^{1}H NMR spectrum, using the known Mw of mPEG (3.58 ppm, 451H) and by comparing its associated peak to those of the hydrogens of the carbobenzyloxy group of PZLL (5.03 ppm and 7.32 ppm for 2H and 5H per unit, respectively). The polymerization ratio of mPEG-*b*-PZLL-*b*-PBLA was calculated by a similar method, which further accounted for the hydrogens on the benzyloxy ester group and the main chain amide of PBLA (5.03 ppm, 7.32 ppm, and 4.67 ppm

for 2H, 5H and 1H per unit, respectively). The polydispersity index (PDI) and the Mw of the copolymers were determined by GPC, using DMF as the eluent.

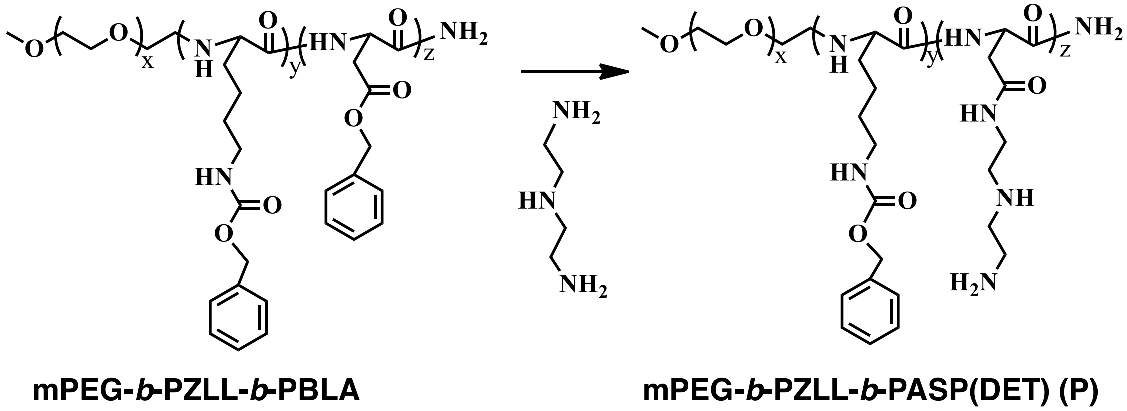

Supplementary Figure 13: Synthesis of mPEG-*b*-PZLL-*b*-PASP(DET) (P).

The triblock copolymer of methoxypoly(ethylene glycol)-*block*-poly(carbobenzyloxy-L-lysine)-*block*-poly{*N*-[*N*-(2-aminoethyl)-2-aminoethyl]aspartamide} (mPEG-*b*-PZLL-*b*-PASP(DET)) was prepared through aminolysis of mPEG-*b*-PZLL-*b*-PBLA upon addition of diethylenetriamine, using a previously reported method[2]. ^{1}H NMR spectroscopy of the product confirmed that all of the pendant benzyl ester groups of the PBLA segments had been aminolysed.

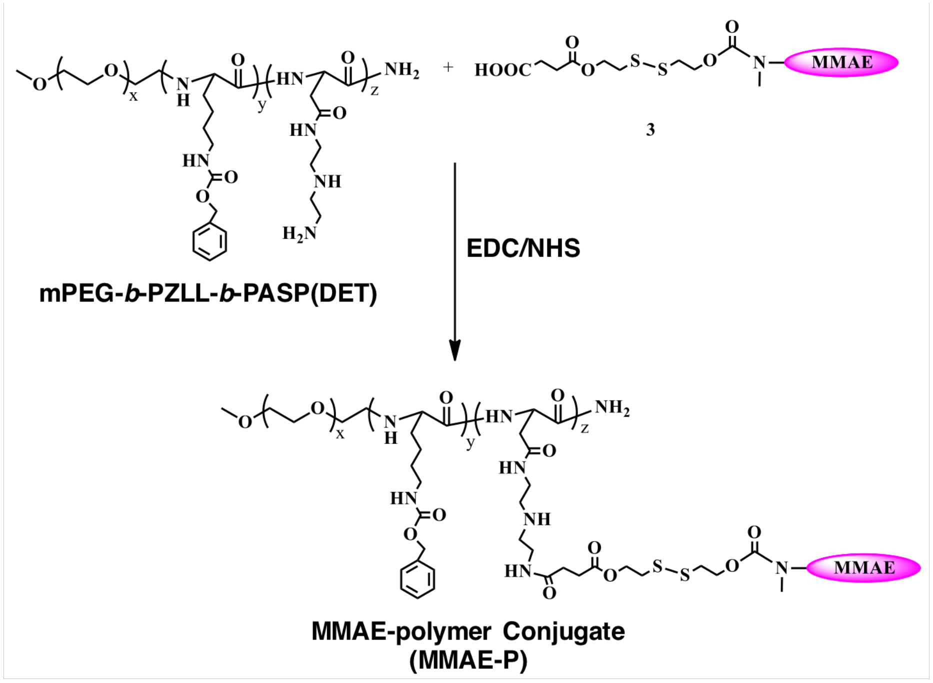

Supplementary Figure 14: Synthesis of the MMAE-polymer conjugate of mPEG-*b*-PZLL-*b*-PASP(DET)-MMAE$_2$ (MMAE-P).

The triblock copolymer of mPEG-*b*-PZLL-*b*-PASP(DET) (40 mg, 2.9 μmol) was combined with DIPEA (10 μl, 60 μmol) in MeOH/dichloromethane (5 mL, 1:2 v/v). A solution containing Compound 3 (9 mg, 9 μmol) and EDC/NHS (1.5 equiv. to Compound 3) in DMF (2 mL) was added drop-wise to the mPEG-*b*-PZLL-*b*-PASP(DET) solution. This mixture was stirred for 24 h, at 4 ^{o}C, and in the dark. Thereafter, the product was precipitated in diethyl ether (100 mL), isolated by centrifugation, dissolved in MeOH/water (2:1 v/v), dialyzed at 4 ^{o}C against 0.01 M HCl overnight, and further dialyzed against deionized water for 2 days. The final product, consisting of the MMAE-conjugated polymer (MMAE-P), was obtained after lyophilization. The molar ratio of MMAE to mPEG-*b*-PZLL-*b*-PASP(DET) in MMAE-P was determined to be 2; it was calculated from the integrated peak of the hydrogen of MMAE (25H, 0.8-1.1 ppm) and by comparison to the peaks corresponding to the degrees of polymerization for each monomeric block of mPEG-*b*-PZLL-*b*-PASP(DET).

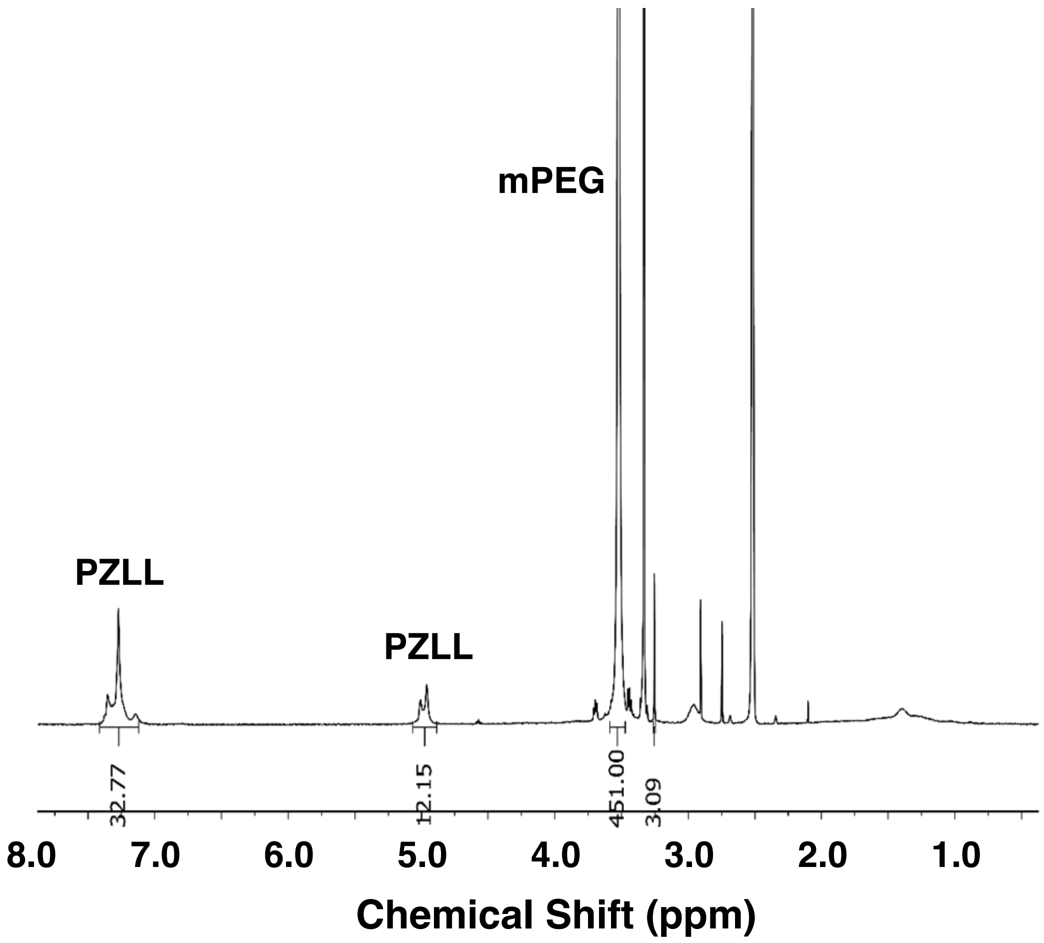

Supplementary Figure 15: ^{1}H NMR spectrum of mPEG-*b*-PZLL (DMSO-d$_6$). The polymerization ratio of mPEG$_{114}$-*b*-PZLL$_6$ was calculated from its ^{1}H NMR spectrum, using the known molecular weight of mPEG (3.58 ppm, 451H) and by comparing its associated peak to those of the hydrogens of the carbobenzyloxy group of PZLL (5.03 ppm and 7.32 ppm for 2H and 5H per unit, respectively).

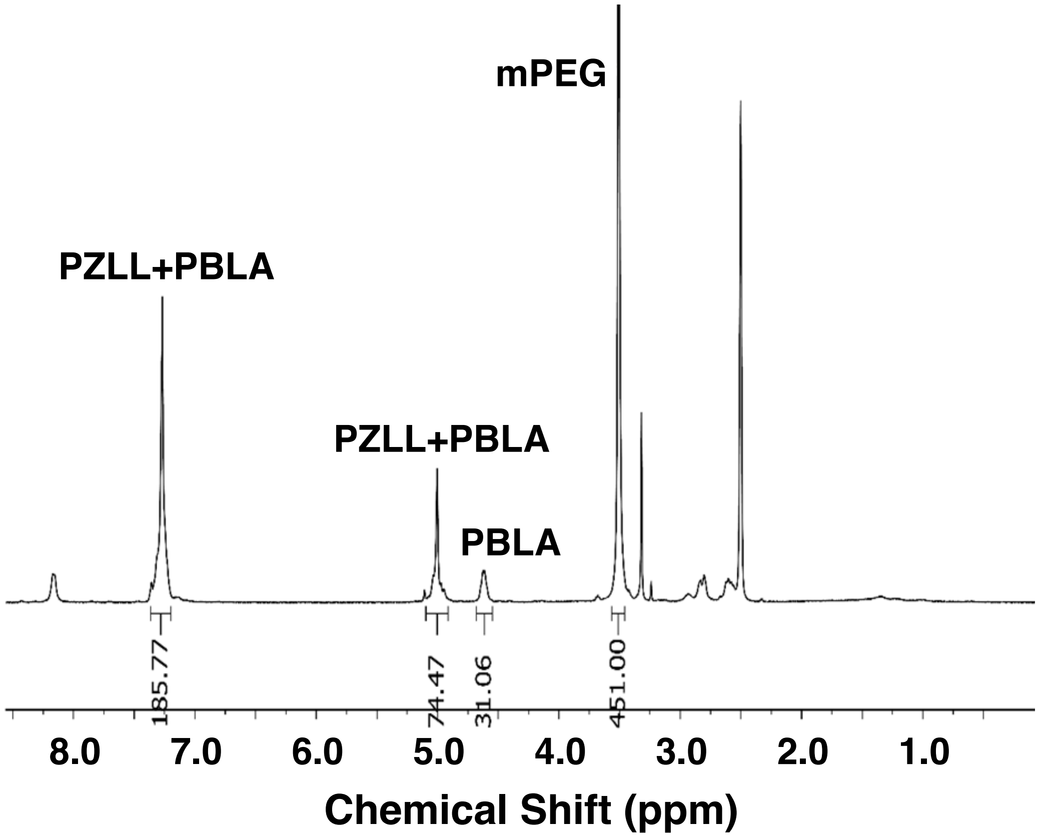

Supplementary Figure 16: ^{1}H NMR spectrum of mPEG-*b*-PZLL-*b*-PBLA (DMSO-d$_6$). The polymerization ratio of $mPEG_{114}$-*b*-$PZLL_6$-*b*-$PBLA_{30}$ was calculated from its ^{1}H NMR spectrum, using the known molecular weight of mPEG (3.58 ppm, 451H) and by comparing its associated peak to those of the hydrogens of the carbobenzyloxy group of PZLL (5.03 ppm and 7.32 ppm for 2H and 5H per unit, respectively) and to the hydrogens on the benzyloxy ester group and the main chain amide of PBLA (5.03 ppm, 7.32 ppm, and 4.67 ppm for 2H, 5H and 1H per unit, respectively).

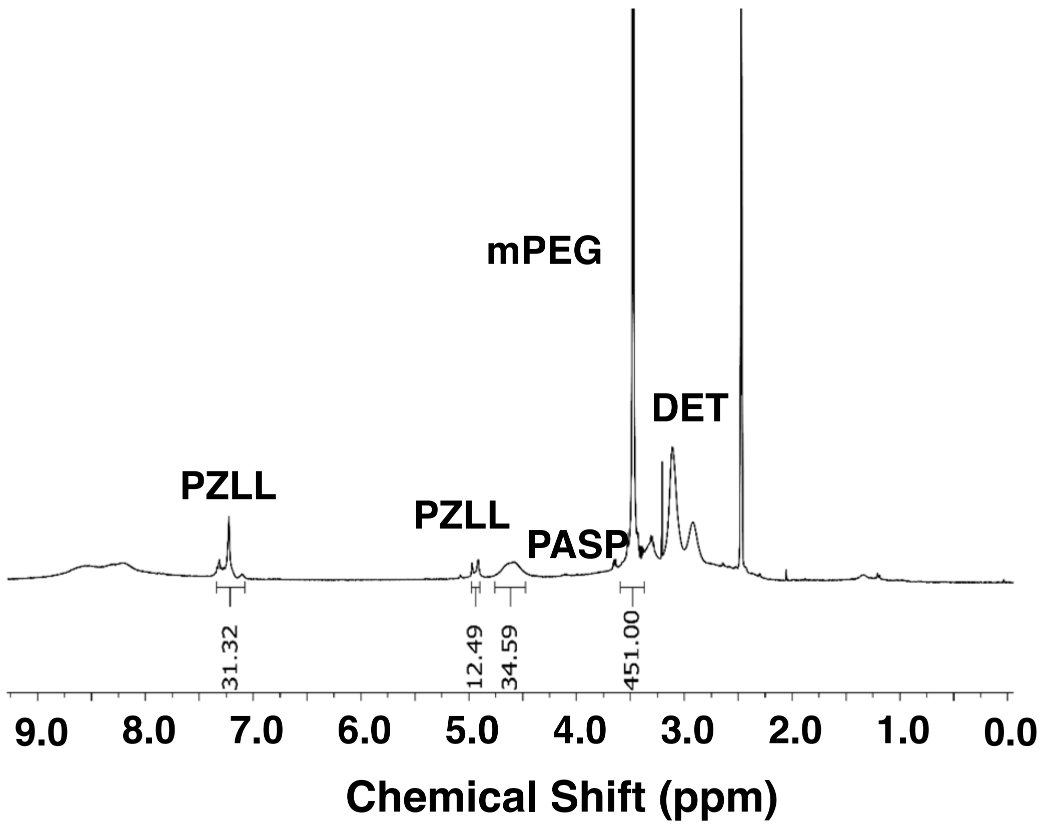

Supplementary Figure 17: ^{1}H NMR spectrum of mPEG-*b*-PZLL-*b*-PASP(DET) (P) (DMSO-d_6). The triblock copolymer of $mPEG_{114}$-*b*-$PZLL_6$ -*b*-$PASP(DET)_{30}$ was prepared through aminolysis of $mPEG_{114}$-*b*-$PZLL_6$ -*b*-$PBLA_{30}$ by diethylenetriamine. The reduced hydrogens on the benzyloxy ester group of PBLA (5.03 ppm and 7.32 ppm per unit), the integrating main chain amide of PASP (4.62 ppm), and the new broad peak of DET confirmed that all of the pendant benzyl ester groups of the PBLA segments had been aminolysed.

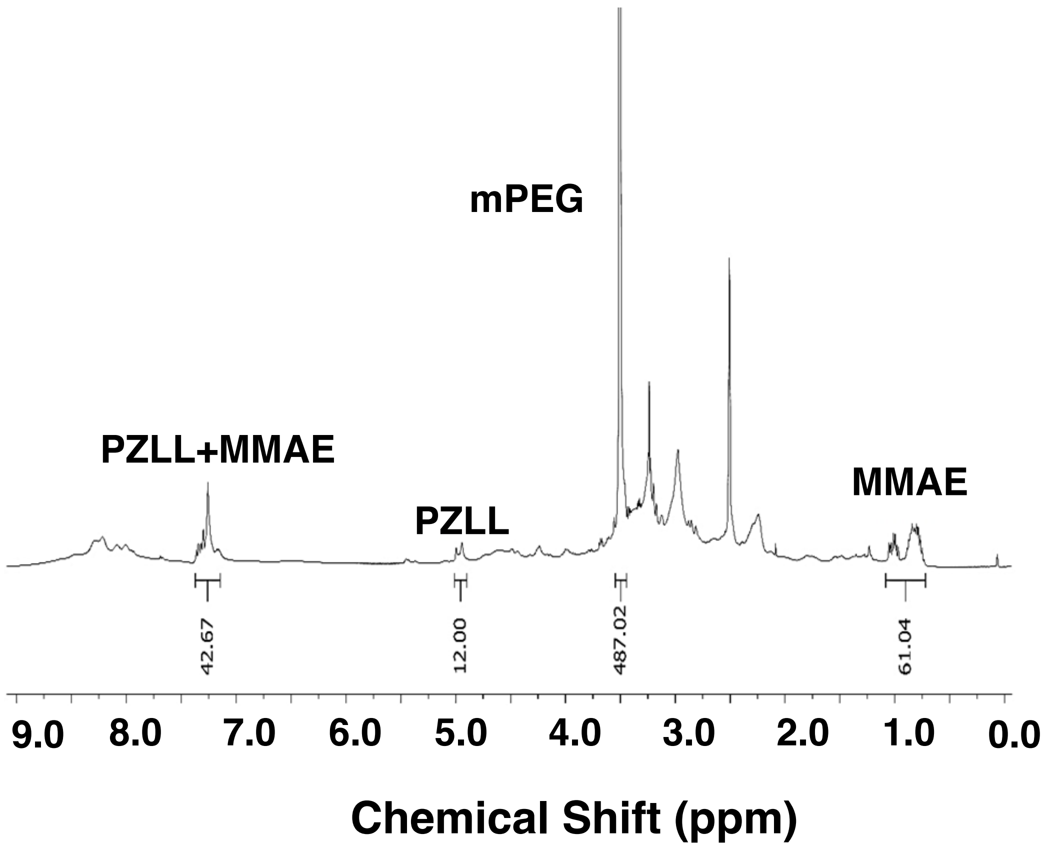

Supplementary Figure 18: ^{1}H NMR spectrum of mPEG-*b*-PZLL-*b*-PASP(DET)-MMAE$_2$ (MMAE-P) (DMSO-d$_6$). The molar ratio of MMAE to mPEG$_{114}$-*b*-PZLL$_6$-*b*-PASP(DET)$_{30}$ in MMAE-P was determined to be 2 and was calculated from the integrated peak of the hydrogen of MMAE (25H, 0.8-1.1 ppm) and by comparison to the peaks corresponding to the degrees of polymerization for each monomeric block of mPEG-*b*-PZLL-*b*-PASP(DET).

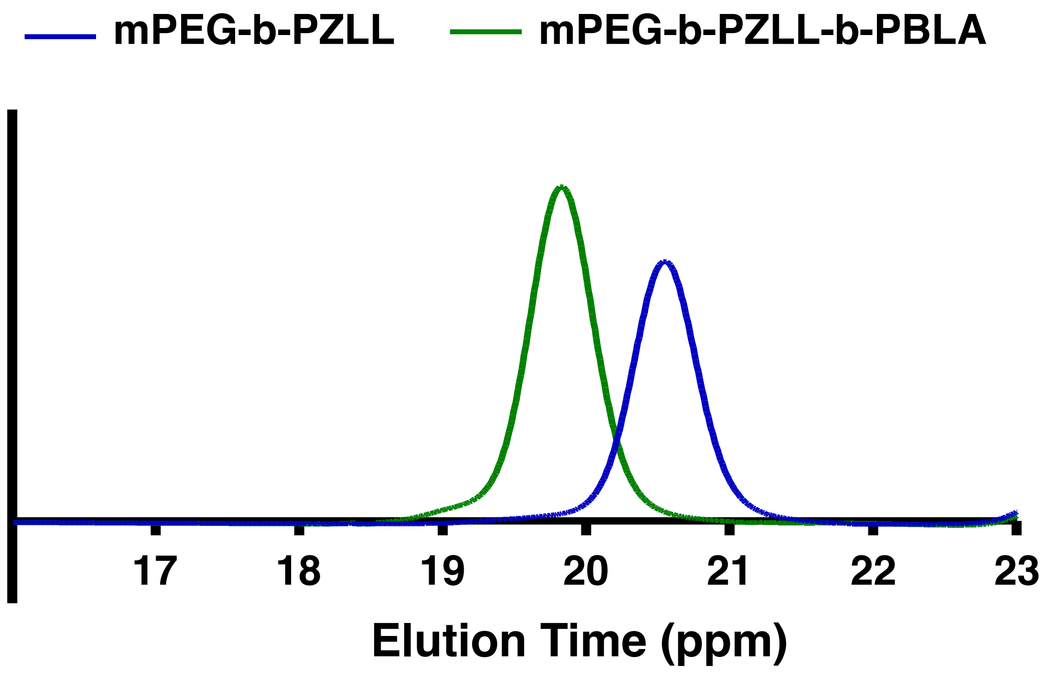

Supplementary Figure 19: GPC traces of mPEG$_{114}$-*b*-PZLL$_6$ and mPEG$_{114}$-*b*-PZLL$_6$-*b*-PBLA$_{30}$. GPC measurements were performed on an Agilent 1260 LC system equipped with a Shamdex KD-806M GPC column; the samples were run at 60 °C and at a flow rate of 1 mL/min. Filtered DMF containing 0.2 M LiBr was used as the eluent.

Supplementary Table 1: Molecular weights and polydispersity indices of copolymers characterized by ^{1}H NMR and by GPC.

Name	Mn^a (kDa)	Mn^b (kDa)	PDI^c
mPEG$_{114}$-*b*-PZLL$_6$	6.6	6.8	1.11
mPEG$_{114}$-*b*-PZLL$_6$-*b*-PBLA$_{30}$	12.9	14.5	1.13
mPEG$_{114}$-PZLL$_6$-PAsp(DET)$_{30}$	12.8		
mPEG$_{114}$-*b*-PZLL$_6$-*b*-PASP(DET)$_{30}$_MMAE$_2$	14.7		
mPEG$_{114}$-PGA$_{30}$	8.9		

a Number-average molecular weight (Mn) as determined by ^{1}H NMR.
b Number-average molecular weight (Mn) as determined by GPC.
c Polydispersity index as determined by GPC (Mw/Mn).

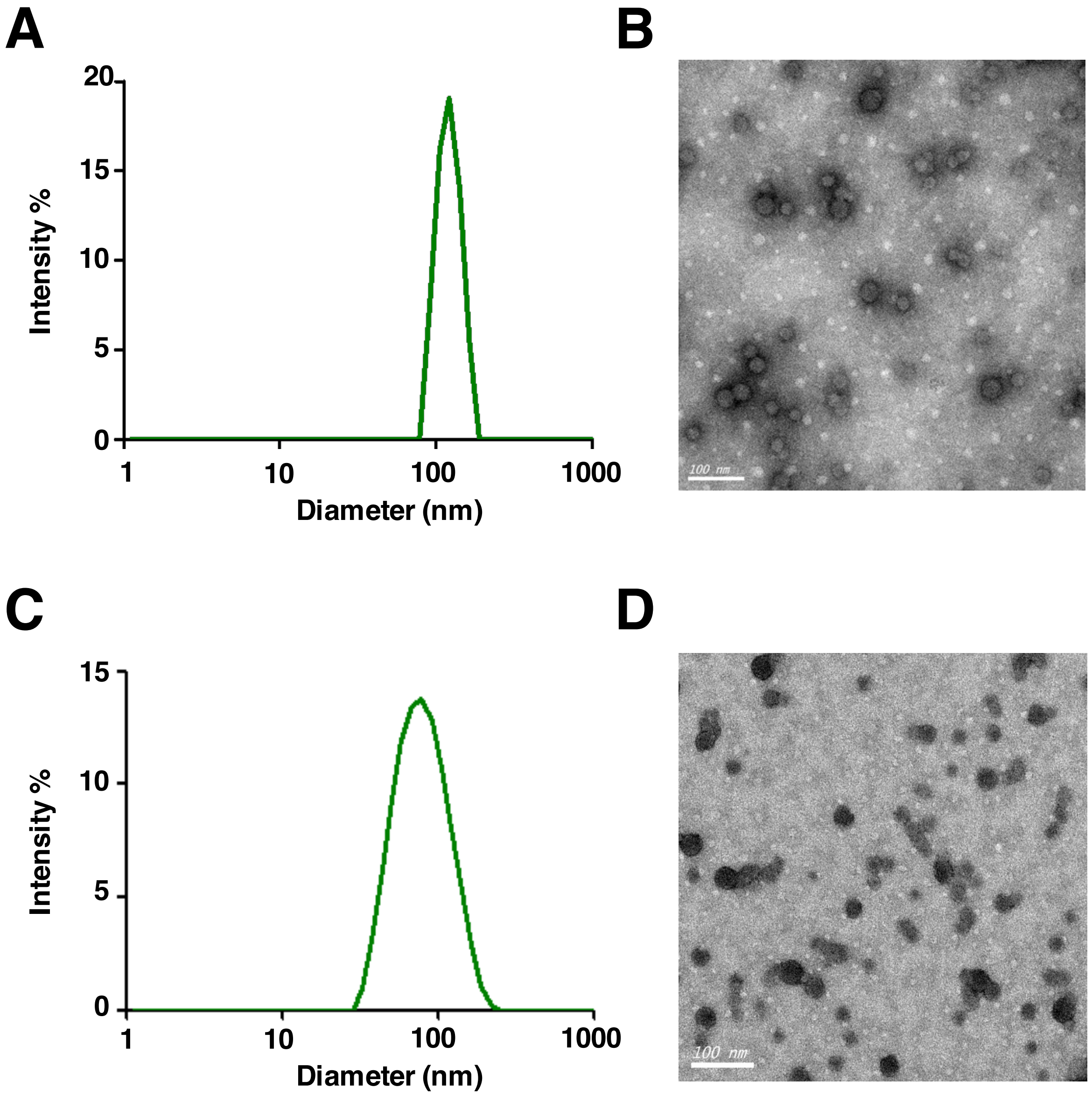

Supplementary Figure 20: Size and morphology of unmodified nanoparticles and NP(MMAE). Measurements of the hydrodynamic size distribution and morphologies of unmodified NPs formed from mPEG$_{114}$-*b*-PZLL$_6$-*b*-PASP(DET)$_{30}$ were conducted by A) dynamic light scattering (DLS) and by B) transmission electron microscopy (TEM), respectively. C) Hydrodynamic size distribution and D) TEM micrograph of a suspension of NP(MMAE). Scale bar = 100 nm.

Supplementary Table 2: Physicochemical properties of unmodified nanoparticles (NP) and MMAE-conjugated nanoparticles (NP(MMAE))

Sample	Diameter by TEM* (nm)	Diameter by DLS** (nm)	PDI	Zeta Potential (mV)
NP	34.2 +/- 5.7	120.1 +/- 4.2	0.118 ± 0.06	43.2 ± 0.9
NP(MMAE)	53.7 ± 7.3	93.5 ± 7.4	0.184 ± 0.05	27.8 ± 0.2

*As determined by TEM measurements of at least 20 separate nanoparticles from 3 individual imaging fields.

**As determined from DLS histograms.

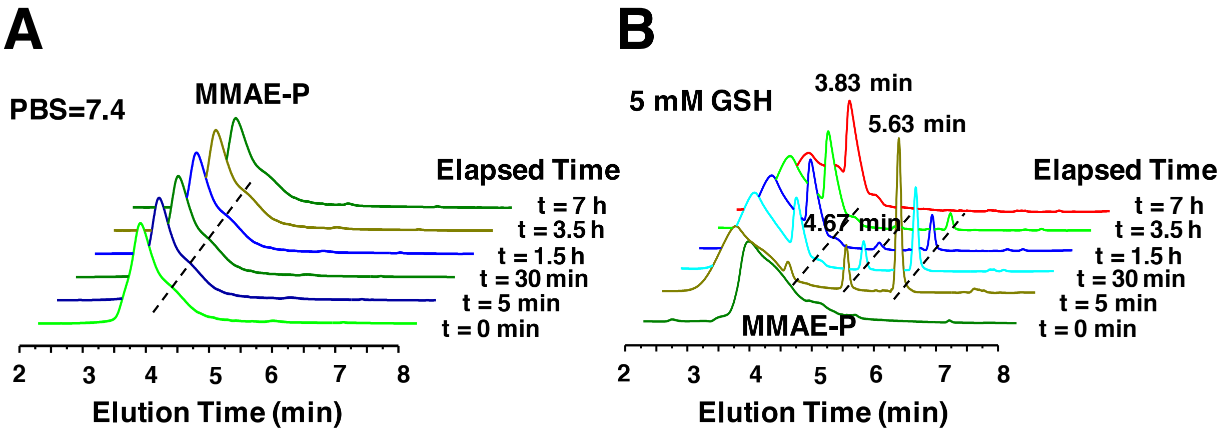

C

Retention Time (min)	m/z	Chemical Structure and Identification	Calculated Mass (g/mol)
5.63	998.5 $(M+H)^+$<br>499.8 $(M+2H)^{2+}$	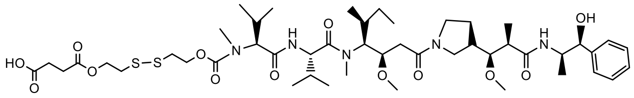<br>Compound 3	997.5
4.67	1127.5 $(M+H)^+$<br>564.4 $(M+2H)^{2+}$	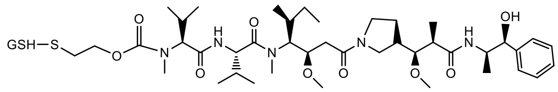<br>Compound 4	1126.5
3.83	718.5 $(M+H)^+$<br>359.8 $(M+2H)^{2+}$<br>740.5 $(M+Na)^+$	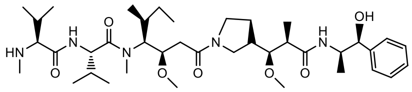<br>MMAE	717.5

Supplementary Figure S21: Release profile of MMAE from NP(MMAE) in different *in situ* environments. Nanoparticles formed from MMAE-P (NP(MMAE)) were generated immediately after its dissolution in water. NP(MMAE) was thereafter incubated with either: A) PBS with or B) PBS containing 5 mM glutathione (GSH), at 37 °C and under Ar. At specified time points, 10 µL of the reaction mixtures were extracted and subject to LC-MS. C) Table summarizing the retention times of reaction products of NP(MMAE) and GSH and as recorded by HPLC with *Method 1*; the mass peaks of intermediate residues that were isolated, as well as the putative chemical structures, identifications, and calculated masses of their associated compounds, are reported.

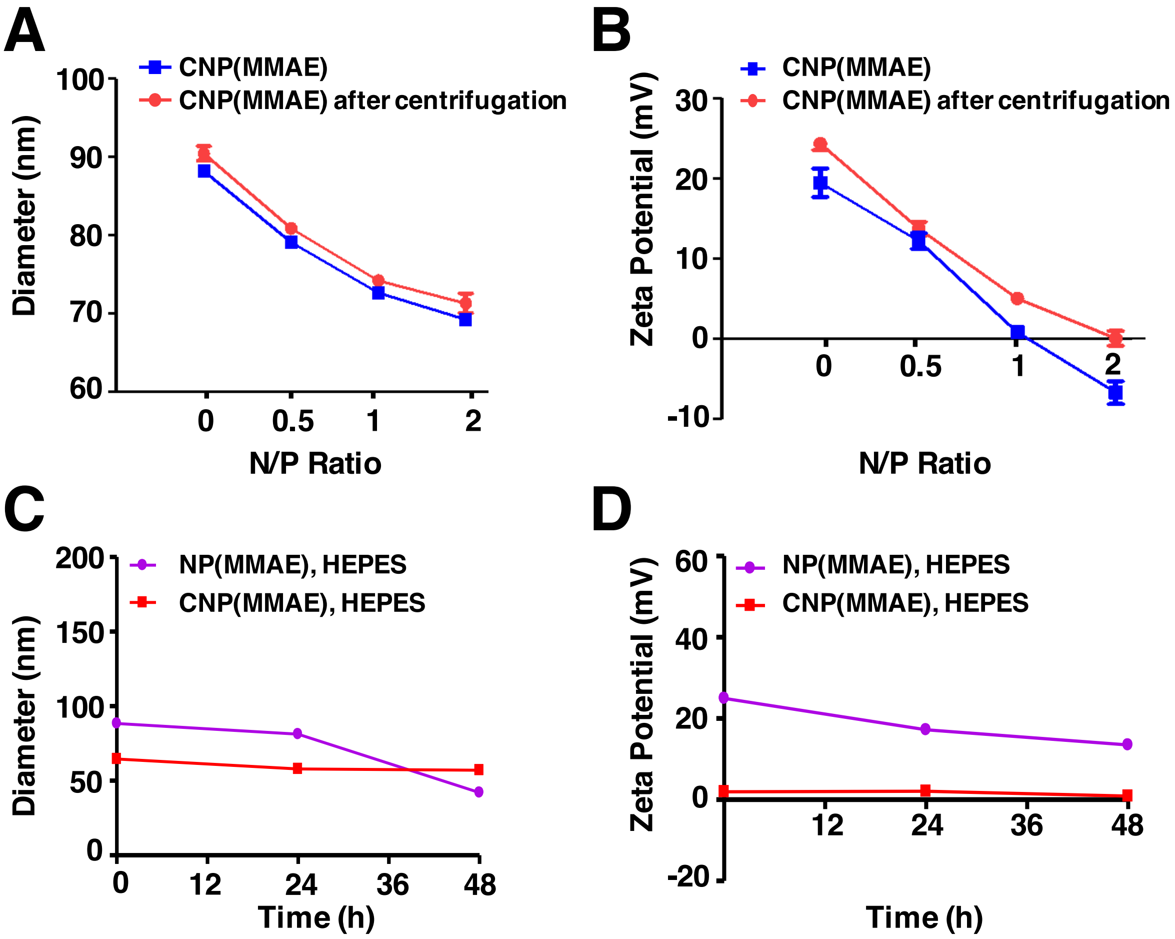

Supplementary Figure 22: Optimization of the coating of NP(MMAE) to generate CNP(MMAE) and examination of the stability of the colloidal assembly over time. CNP(MMAE) was formed by mixing mPEG-*b*-PGA with NP(MMAE) at different initial molar ratios of negatively charged (mPEG$_{114}$-*b*-PGA$_{30}$) to positively charged (MMAE-P) polymer (N/P ratio). A) the average hydrodynamic diameters (as assessed by DLS) and B) the surface charges (as determined by zeta potential measurements) of CNP(MMAE)) were examined before and after a centrifugation filtration step that was employed to remove any non-complexed (i.e. free) mPEG$_{114}$-*b*-PGA$_{30}$ in suspension. The stability of NP(MMAE) and CNP(MMAE) (after centrifugation filtration) were measured in HEPES buffer at 37 °C and over the course of 48 h by C) serial DLS and D) zeta potential measurements of small volume aliquots taken at 12 h intervals.

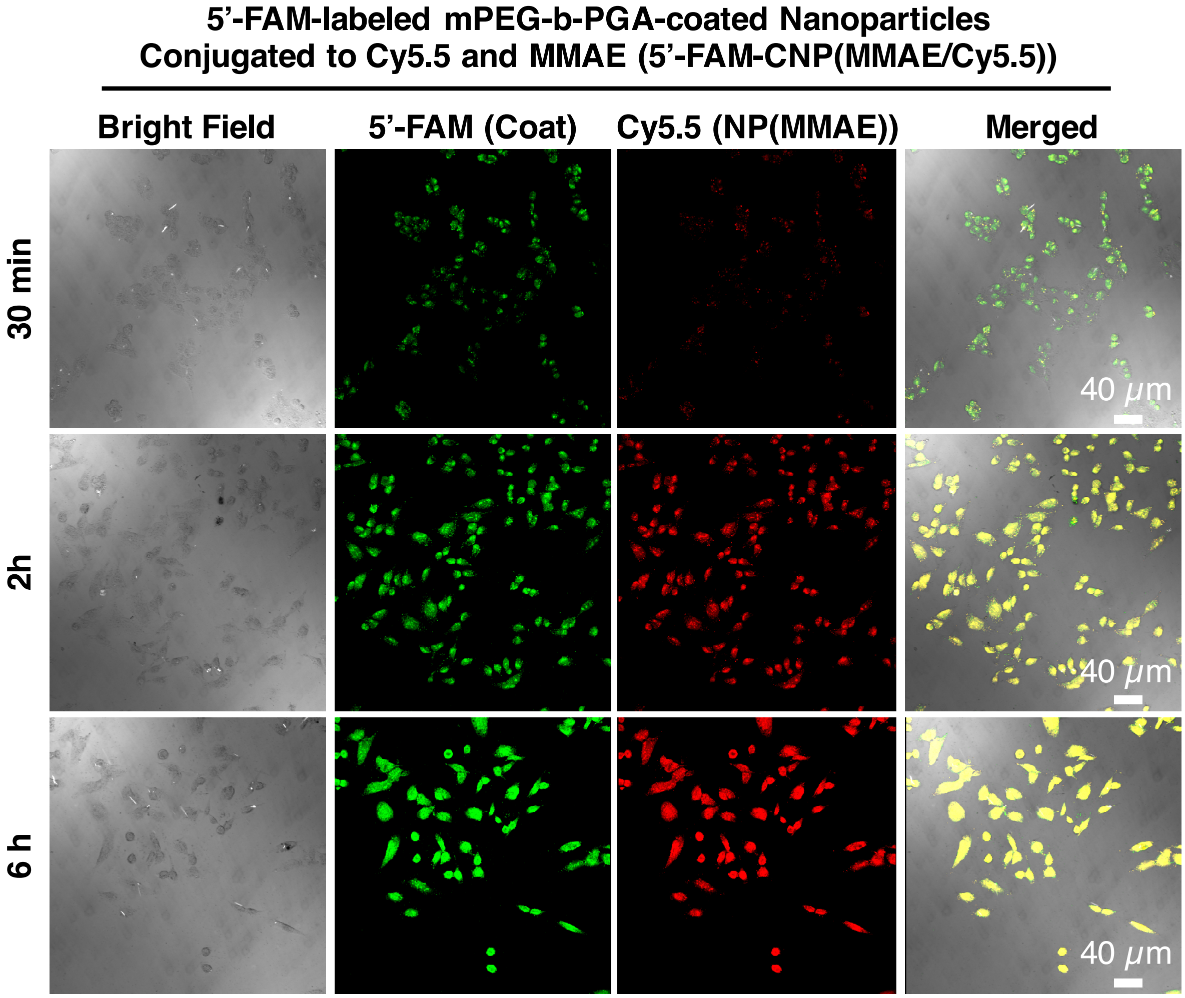

Supplementary Figure 23: Determination of the stability and uptake of coated and MMAE-conjugated nanoparticles by confocal microscopy. 5'-FAM was used to label the $mPEG_{114}$-b-PGA_{30} polymer and Cy5.5 was conjugated to NP(MMAE) prior to formation of the resultant dual-fluorophore-labeled, coated, and MMAE-containing NPs (5'-FAM-CNP(MMAE/Cy5.5)). OVCAR8 cells were seeded onto glass slides in six-well plates (4×10^5 cells/well) and allowed to adhere overnight. The cells were then treated with 5'-FAM-CNP(MMAE/Cy5.5) and the uptake of each component of this colloidal assembly was independently monitored: i.e. the fluorescence of the 5-FAM-conjugated $mPEG_{114}$-b-PGA_{30} polymer (5'-FAM (Coat); green) and the Cy5.5-lableled NP(MMAE) (Cy5.5 (NP(MMAE)), red) were imaged at each time point, using an Olympus FV1100 confocal laser scanning microscopy imaging system. Note that the concentration of 5'-FAM and Cy5.5 were held constant throughout (both were present at 1 μg/mL). The time dependent increases in both red and green fluorescence and the perfect co-localization of the two colors supported the stability of CNP(MMAE) structure during the cellular internalization process. Scale bar = 40 μm.

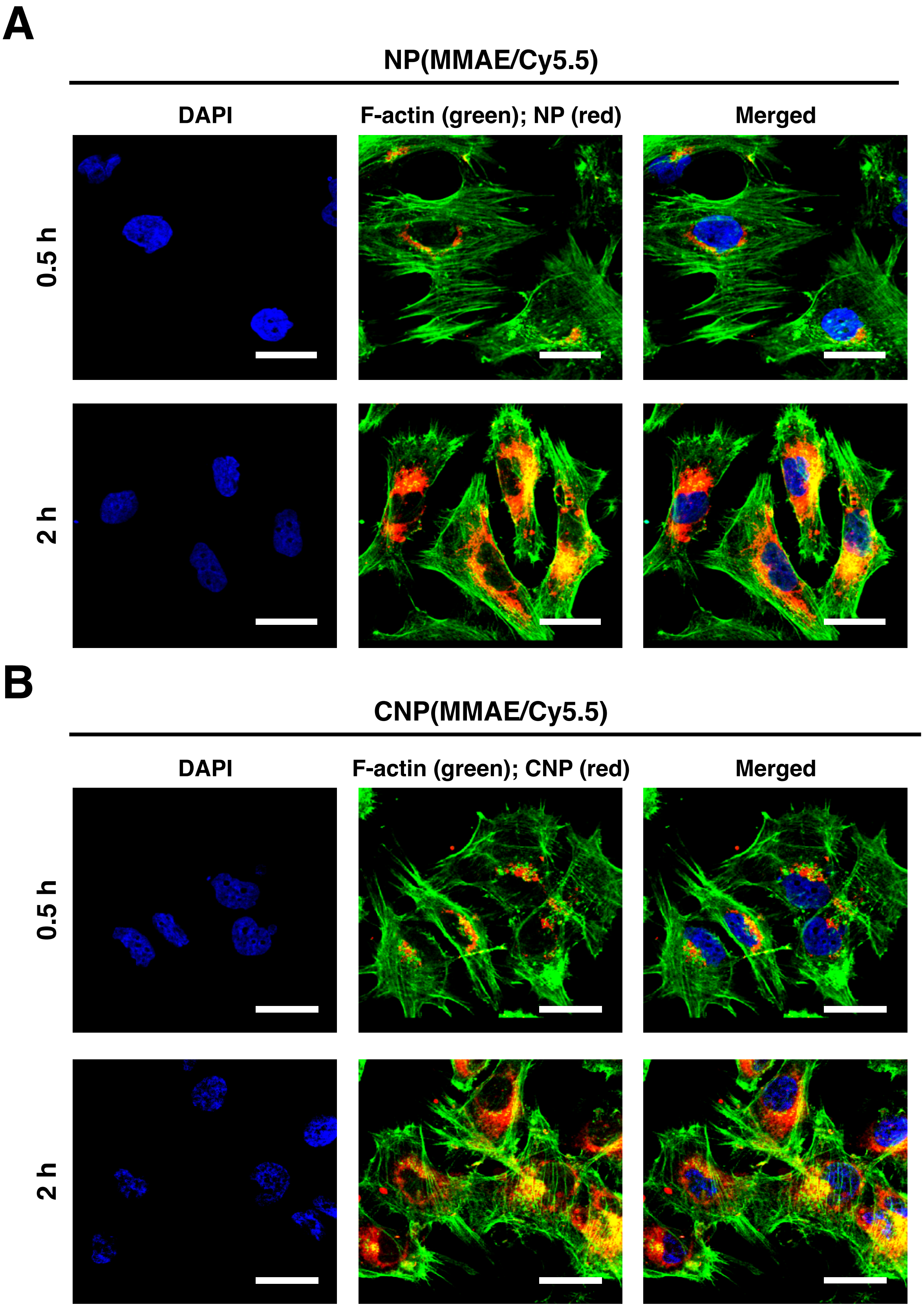
A
NP(MMAE/Cy5.5)
DAPI
F-actin (green); NP (red)
Merged
0.5 h
2 h
B
CNP(MMAE/Cy5.5)
DAPI
F-actin (green); CNP (red)
Merged
0.5 h
2 h

Supplementary Figure 24: Comparisons of the relative intracellular uptake of MMAE-containing nanoparticles by confocal microscopy. OVCAR8 cells were seeded in six-well plates (2×10^5 cells/well) and allowed to adhere overnight. The cells were then incubated with Cy5.5-labeled, uncoated, and MMAE-conjugated NPs (NP(MMAE/Cy5.5); top) or with Cy5.5-labeled, $mPEG_{114}$-b-PGA_{30}-coated, and MMAE-conjugated NPs (CNP(MMAE/Cy5.5); bottom) for either 30 min or for 2 h. The MMAE (50 nM) and Cy5.5 concentrations (1 μg/mL) in all NP suspensions were kept constant. After the aforementioned incubation periods, the cells were thoroughly washed; their nucleic were stained with DAPI; and, Alexa488-phalloidin was used to stain for F-actin. The cells were placed on mounted coverslips and the slides were imaged with an Olympus FV1100 confocal laser scanning microscopy imaging system. Scale bar = 20 μm.

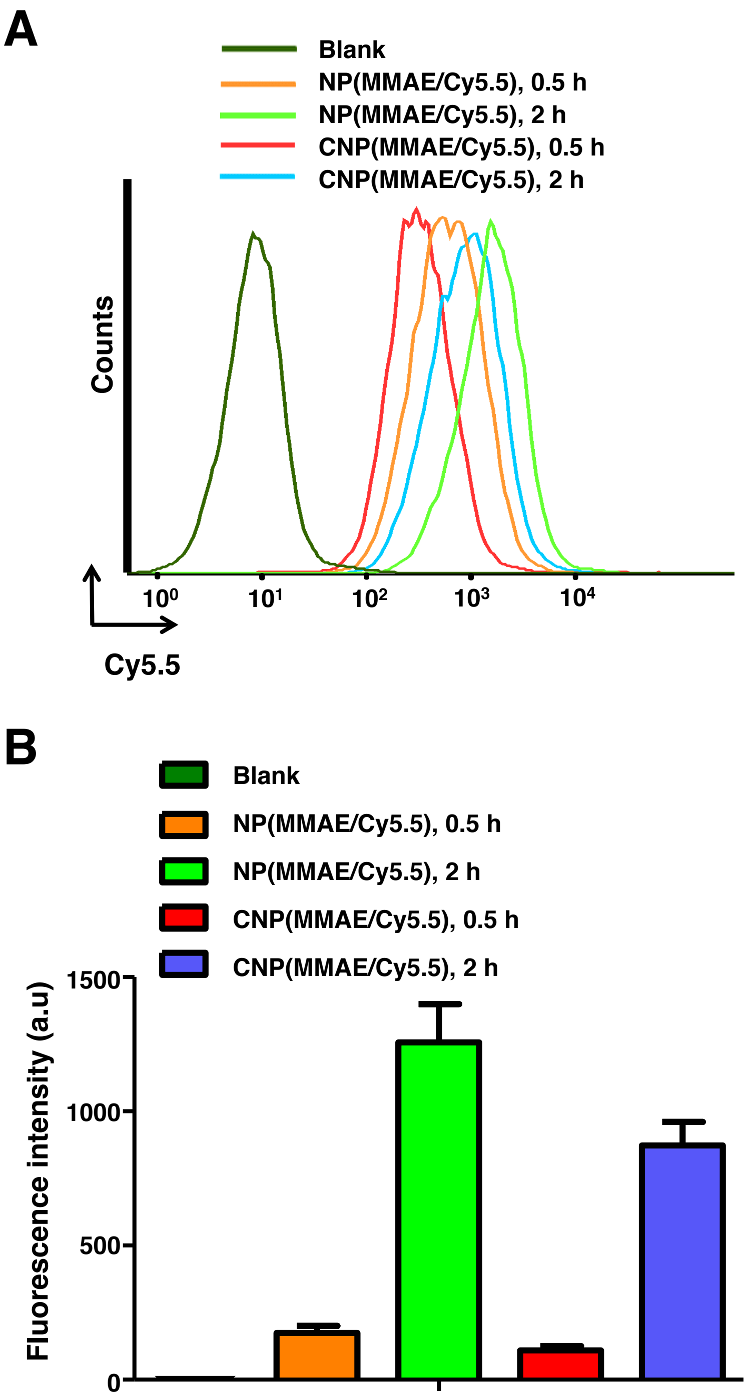

Supplementary Figure 25. Comparisons of the relative intracellular uptake of MMAE-containing nanoparticles by flow cytometry. Flow cytometry measurements were used to compare the relative uptake of uncoated and $mPEG_{114}$-b-PGA_{30}-coated NPs after labeling with Cy5.5 (i.e. NP(MMAE/Cy5.5) as compared to CNP(MMAE/Cy5.5), respectively). OVCAR8 cells were seeded in six well plates (5×10^5 cells/well) and allowed to adhere overnight. The cells were then incubated with NP(MMAE/Cy5.5) or CNP(MMAE/Cy5.5) at a fixed concentration of Cy5.5 (1 µg/mL) and for either 0.5 or 2 h. After washing, the relative intracellular uptake of the different NPs was quantified by determination of Cy5-5 fluorescence, using flow cytometry.

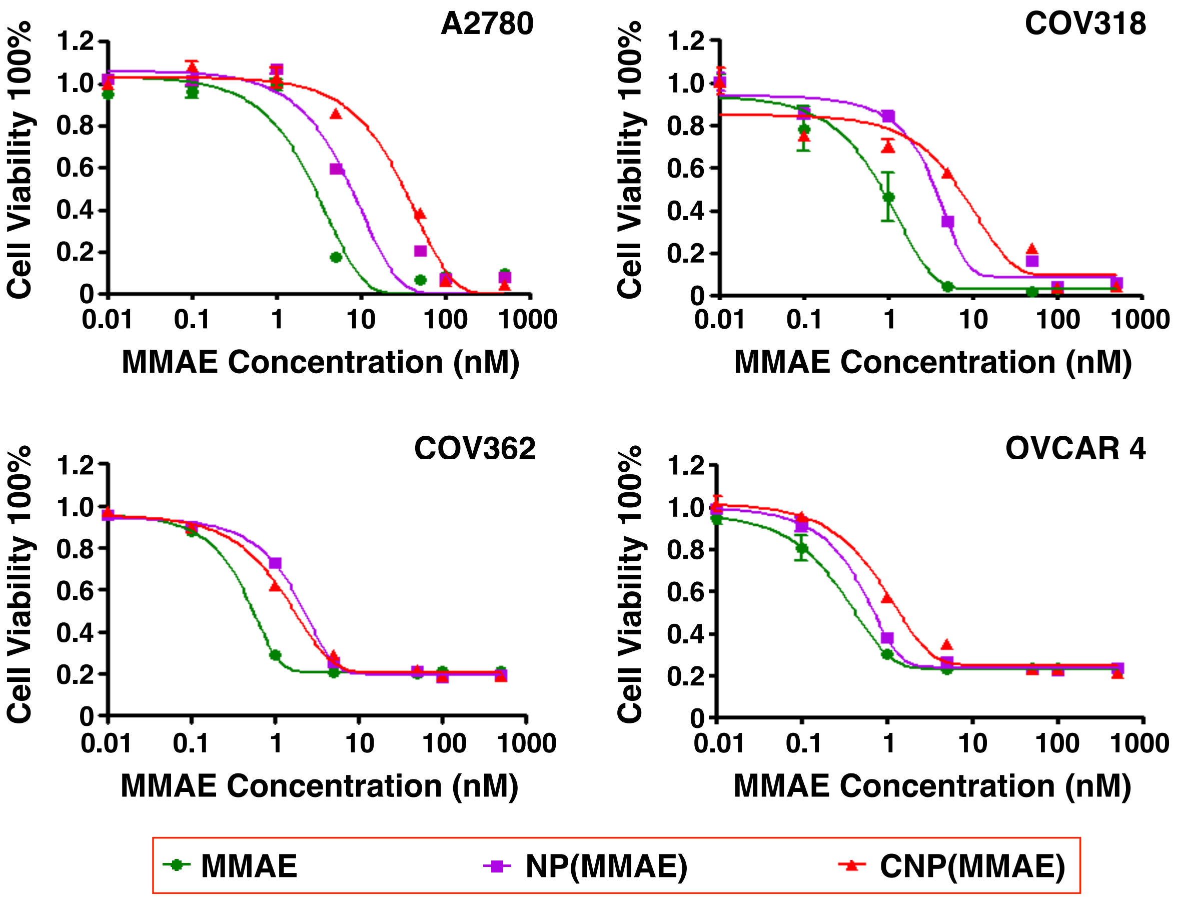

Supplementary Figure 26: Evaluation of the relative *in vitro* cytotoxicity of various formulations of MMAE. A panel of ovarian cancer cell lines (A2780, COV362, COV318, OVCAR4 and OVCAR8) was used to assess the relative cytotoxicities of the free drug formulation of MMAE (MMAE), NP(MMAE) and CNP(MMAE); note that the results obtained with OVCAR8 cells are shown in Figure 3A in the main manuscript. The cells were plated at a density of 5k cells/well in 96-well plates; they were allowed to adhere for 24 h; and, they were subsequently incubated with each formulation at a fixed MMAE concentration and for an additional 72 h. At the end of the incubation period, relative cellular viability was determined, using the colorimetric MTT assay, and was plotted after normalization to the results obtained with untreated cells.

Supplementary Table 3: IC_{50} values from multiple ovarian cancer cell lines that were treated with free MMAE and two different nanoparticle-based formulations (NP(MMAE) and CNP(MMAE)).

Cell Line	IC_{50} (nM)		
	MMAE	NP(MMAE)	CNP(MMAE)
A2780	3.65 ± 0.10	11.45 ± 0.19	27.56 ± 0.17
COV362	0.44 ± 0.04	3.86 ± 0.07	4.29 ± 0.10
COV318	0.92 ± 0.52	4.71 ± 0.18	10.35 ± 0.27
OVCAR 4	0.24 ± 0.19	0.87 ± 0.04	3.74 ± 0.07
OVCAR 8	0.71 ± 0.02	2.72 ± 0.06	3.49 ± 0.12

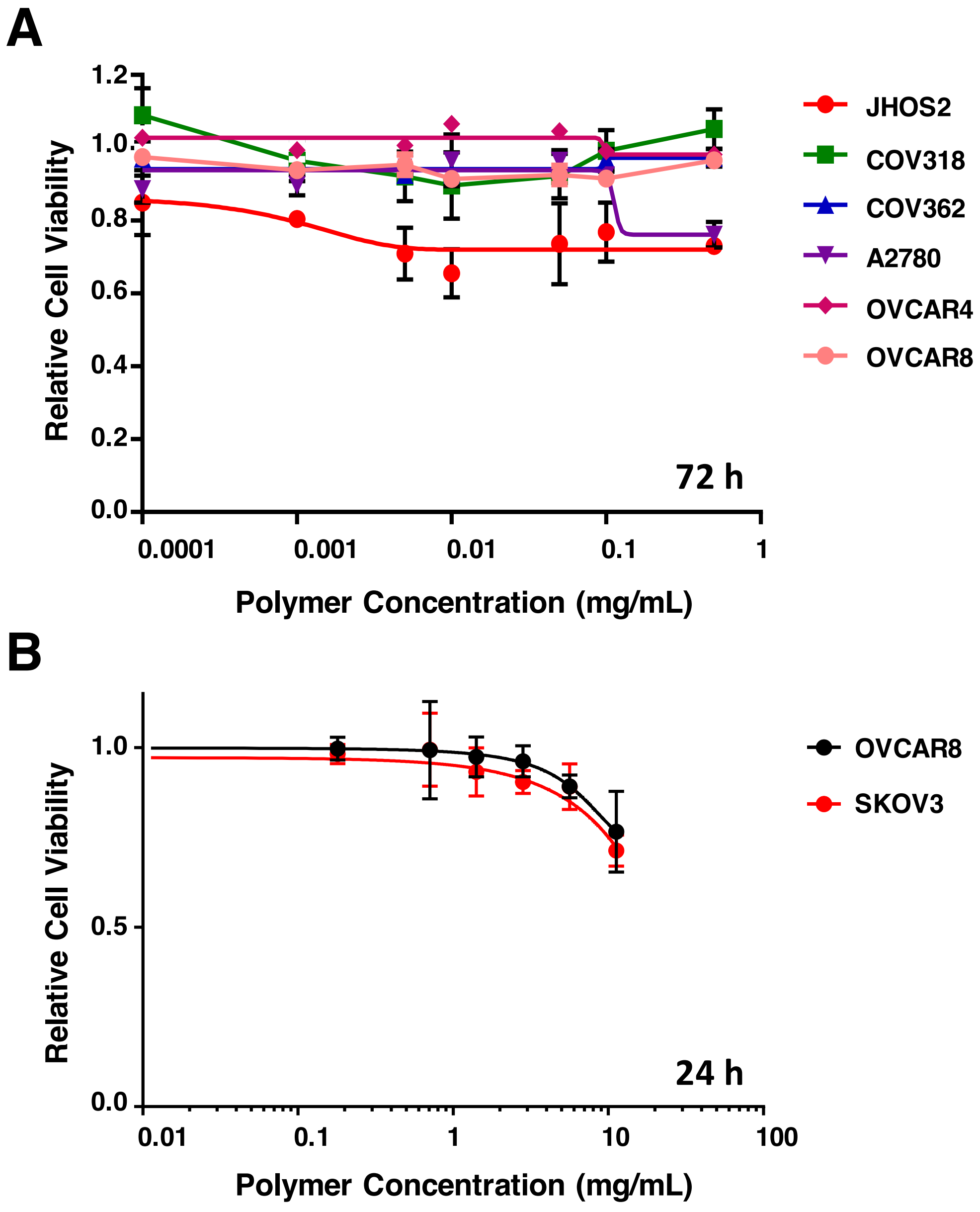

Supplementary Figure 27: Empty nanoparticles (i.e. nanoparticles without MMAE) do not impart cytotoxic effects to ovarian cancer cells in culture. Different ovarian cancer cell lines (e.g. JHOS2, COV318, COV362, A2780, OVCAR4, OVCAR8, and SKOV3) were each seeded in 96-well plates (5,000 cells/well) and allowed to adhere for 24 h. Thereafter, the cells were incubated with empty NPs at different concentrations of polymer (i.e. mPEG-*b*-PZLL-*b*-PASP(DET)) and cellular viability was measured by the colorimetric MTT assay. Cellular viability at A) 72 h and B) 24 h was plotted for various cell lines treated with empty nanoparticles as a function of polymer concentration; note that the results were normalized to those obtained with the corresponding cell lines in the absence of nanoparticle treatment.

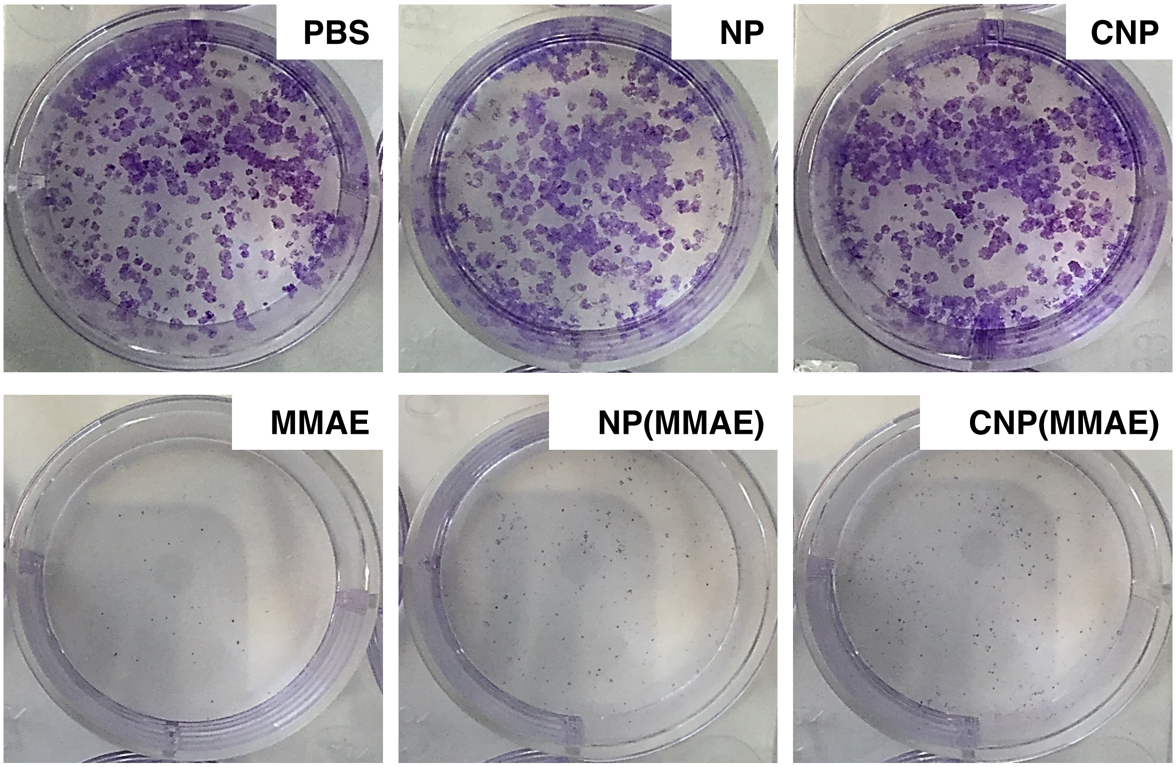

Supplementary Figure 28: Colony formation assay for proliferation performed on OVCAR8 cells after *in vitro* treatment with various formulations of MMAE. OVCAR8 cells (1×10^3 cells/well) were treated with the free drug formulation of MMAE (MMAE), NP(MMAE), or CNP(MMAE) for 7 days and at a fixed MMAE concentration (5 nM); PBS, empty uncoated nanoparticles (NP) and empty coated nanoparticles (CNP) served as control treatments and were incubated with the cells at equal volumes and/or polymer concentrations. Following the 7 days of incubation with each treatment group, tumor cell colonies were fixed and stained with 0.5% gentian violet PBS solution and photographs were taken under illumination. The numbers of confluent colonies were enumerated, using ImageJ software.

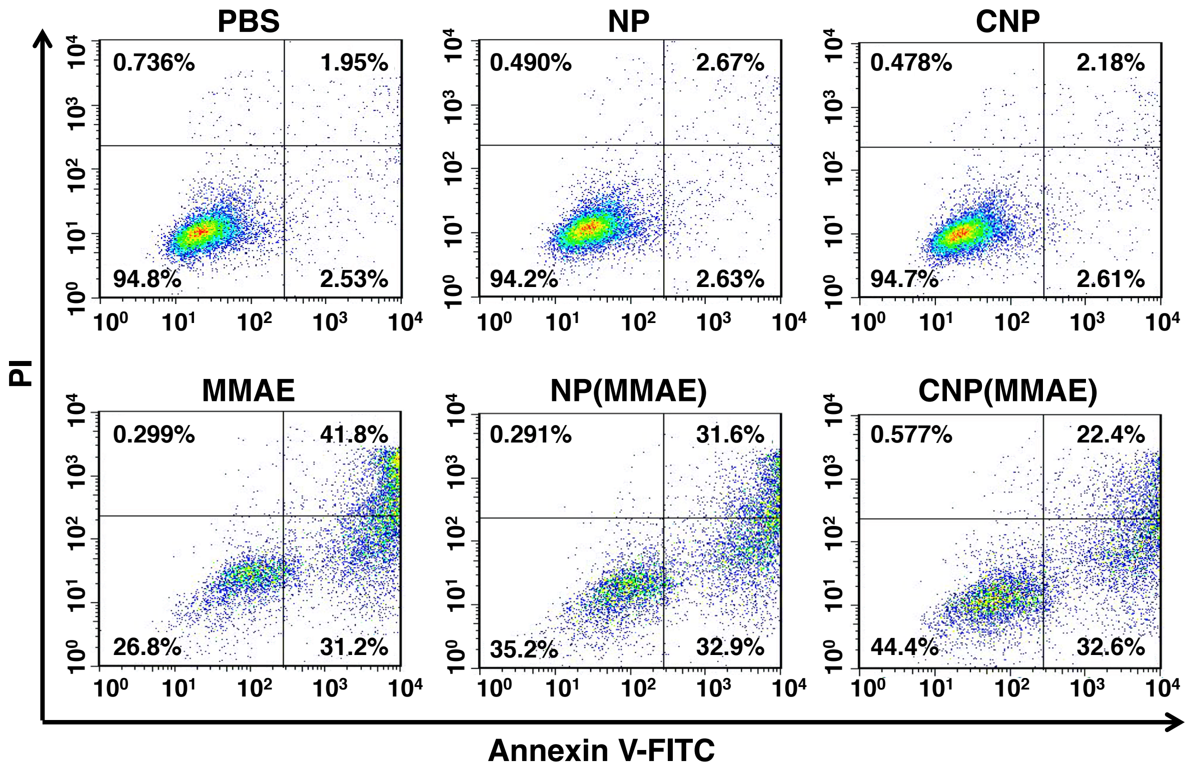

Supplementary Figure 29: Flow cytometry-based apoptosis assay performed on OVCAR8 cells after *in vitro* treatment with various MMAE-containing and control formulations. OVCAR8 cells were seeded in 6-well plates (5×10^5 cells/well) and allowed to adhere overnight. The cells were then treated with the free drug formulation of MMAE (MMAE), NP(MMAE) or CNP(MMAE) for 48 h and at a fixed MMAE concentration (5 nM). PBS, empty uncoated nanoparticles (NP) and empty coated nanoparticles (CNP) served as control treatments and were incubated with the cells at equal volumes and/or polymer concentrations. Following the 48 h of incubation with each treatment group, the fractions of apoptotic cells were detected by flow cytometry, using the Annexin V-FITC Apoptosis Detection Kit I.

Supplementary Table 4: Enumeration of the fractions of OVCAR8 cells that underwent apoptosis after 48 h of treatment with various MMAE-containing and control formulations.

Compound	% Apoptotic Cells
**PBS**	4.5 ± 1.28
**NP**	5.3 ± 1.14
**CNP**	4.8 ± 1.30
**MMAE**	73.0 ± 2.76
**NP(MMAE)**	64.5 ± 2.57
**CNP(MMAE)**	55.0 ± 2.31

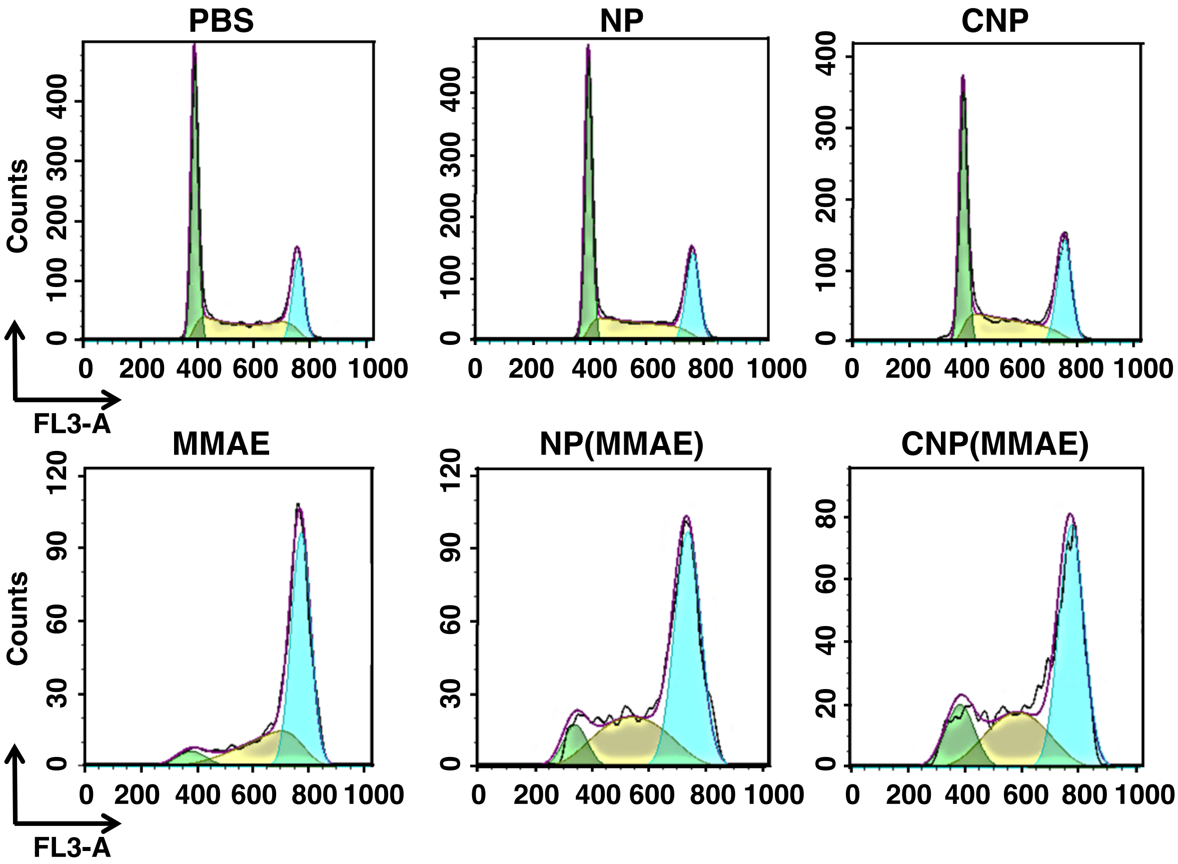

Supplementary Figure 30: Cell cycle distribution of cultured OVCAR8 cells after *in vitro* treatment with various formulations of MMAE. OVCAR8 cells ($5{\times}10^4$ cells/well) were treated with the free drug formulation of MMAE (MMAE), NP(MMAE) or CNP(MMAE) for 48 h and at a fixed MMAE concentration (0.5 nM). PBS, empty uncoated nanoparticles (NP) and empty coated nanoparticles (CNP) served as control treatments and were incubated with the cells at equal volumes and/or polymer concentrations. Following the 48 h of incubation with each treatment group, the cells were fixed and stained with PI. Their DNA content was measured, using flow cytometry; and, cell cycle distribution was determined, using FlowJo software.

Supplementary Table 5: Cell cycle distribution of OVCAR8 cells after treatment with varies MMAE-containing and control formulations.

Treatment	G1%	S%	G2/M%
**PBS**	42.5	35.1	22.4
**NP**	42.3	40.0	25.7
**CNP**	40.5	32.5	27.0
**MMAE**	6.5	25.2	68.3
**NP(MMAE)**	9.5	27.3	63.2
**CNP(MMAE)**	16.5	30.3	53.2

Supplementary Table 6: Maximum tolerated doses (MTDs) of auristatin-based antibody-drug conjugates (ADCs) in mice

Single-dose MTD of Auristatin-based ADCs[1]	Dose Equivalent of Toxin (Bound to the ADC)	Single-dose MTD of Free MMAE[7]	Mouse Strain	Reference
250 mg ADC per kg (2 MMAE per Ab)[2]	2.3 mg MMAE per kg (2 MMAE per Ab)[2]	N/A	BALB/c mice	3
100 mg ADC per kg (4 MMAE per Ab)[3]	1.45 mg MMAE per kg (4 MMAE per Ab)[3]			
50 mg ADC per kg (8 MMAE per Ab)[4]	1.75 mg MMAE per kg (8 MMAE per Ab)[4]			
50 mg ADC per kg (4 MMAF per Ab)	1.4 mg MMAF per kg (4 MMAF per Ab)	N/A	BALB/c mice	4
30 mg ADC per kg (8 MMAE per Ab)[5]	1.1 mg MMAE per kg (8 MMAE per Ab)[5]	0.5-1 mg/kg	CB17 SCID mice	5
30 mg ADC per kg (8 MMAE per Ab single dosage)[6]	1.1 mg MMAE per kg (8 MMAE per Ab)[6]	1 mg/kg	BALB/c mice<br>CB17 SCID mice	6

1: Defined as the highest dose that did not induce >20% weight loss, distress, or overt toxicities in any of the treated animals.

2: Resulted in a maximum of 10.5% weight loss at day 6 after injection.

3: Minimal weight loss at 100 mg/kg ADC; at 120 mg/kg ADC (dose equivalent of 1.74 mg/kg of MMAE), 1 of 3 mice experienced 17% weight loss, significant distress, and were euthanized.

4: Maximum of 14% weight loss by day 6 post-injection; at 60 mg/kg ADC (dose equivalent of 2.1 mg/kg of MMAE), 1 of 3 mice experienced 23% weight loss by day 6 after injection

5: At 40 mg/kg ADC, the mice experienced weight loss of up to 20% within 4 days of injection, at which point 3 of 5 animals were killed. The surviving mice began to regain weight 7 to 10 days after injection and returned to initial body weight by day 16. Doses higher than 40 mg/kg were lethal.

6: Administration of 40 mg/kg ADC resulted in substantial (>20%) weight loss and was not well tolerated.

7: Separate control group used in the corresponding study.

A

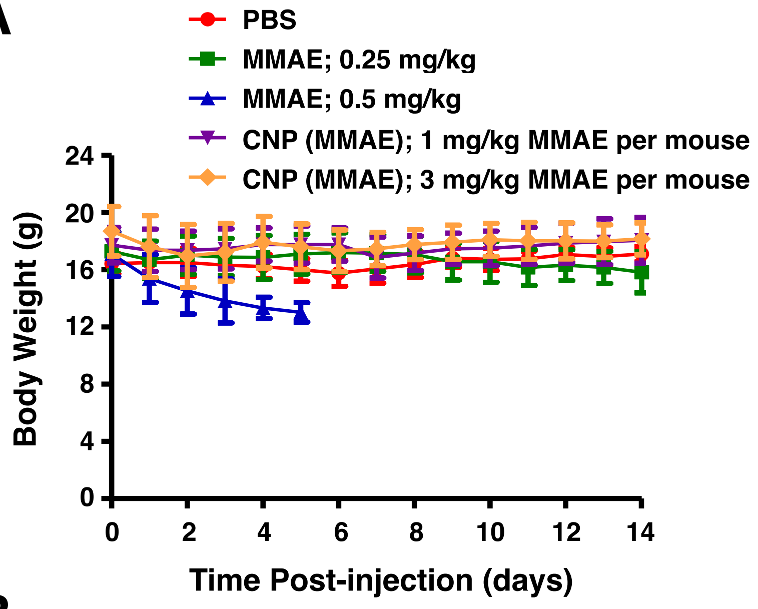

B

TREATMENT GROUP	MICE TAG	BUN	CREATININE
PBS	HX854		
	HX855	24	0
	HX856	20	0
MMAE; 0.25 mg/kg	HX896	26	0
	HX897	18	0
	HX898	21	0
CNP(MMAE); 1 mg/kg MMAE/mouse	HX802	27	0
	HX803		
	HX804	29	0
CNP(MMAE); 3 mg/kg MMAE/mouse	HX805	28	0
	HX810	28	0
	HX812	18	0

C

TREATMENT GROUP	MICE TAG	ALT (SGPT)	AST(SGOT)	ALK PHOSPHATASE	TOTAL BILIRUBIN	DIRECT BILIRUBIN	ALBUMIN
PBS	HX854						
	HX855	151	559	119	0.4	0	2.4
	HX856	105	387	149	0.4	0	2.8
MMAE; 0.25 mg/kg	HX896	170	201	107	0.2	0	2.8
	HX897	54	107	87	0.2	0	2.6
	HX898	60	277	111	0.3	0	2.8
CNP(MMAE); 1 mg/kg MMAE	HX802	44	241	112	0.3	0.1	3
	HX803	49	661				
	HX804	48	252	113	0.4	0.1	3
CNP(MMAE); 3 mg/kg MMAE	HX805	99	442	124	0.4	0	3.1
	HX810	84	446	99	0.4	0	2.9
	HX812	41	251	166	0.3	0	2.9

D

TREATMENT GROUP	MICE TAG	WBC (K/μL)	Hb (g/dL)	Hct (%)	Plt (K/μL)
PBS	**HX854**	6.68	14	60.3	384
	**HX855**	9.72	15.8	65	753
	**HX856**	11.34	14.3	59	584
MMAE; 0.25 mg/kg	**HX896**	6.42	13.4	57.5	841
	**HX897**	5.64	12.7	56.1	800
	**HX898**	5.92	13.3	55.8	780
CNP(MMAE); 1 mg/kg MMAE	**HX802**	5.72	13.5	57.2	794
	**HX803**	6.9	10.3	41.3	348
	**HX804**	6.28	16.3	67.1	837
CNP(MMAE); 3 mg/kg MMAE	**HX805**	5.36	15.8	63.7	776
	**HX810**	6	14.9	61.3	892
	**HX812**	8.64	16.6	68.8	1091

E

TREATMENT GROUP	MICE TAG	Ne (K/μL)	Ly (K/μL)	Mo (K/μL)	Eo (K/μL)	Ba (K/μL)
PBS	**HX854**	1.53	4.6	0.46	0.08	0.02
	**HX855**	2.61	5.48	1.5	0.12	0.01
	**HX856**	2.53	7.74	1.04	0.03	0
MMAE; 0.25 mg/kg	**HX896**	2.13	3.76	0.42	0.09	0.02
	**HX897**	1.67	3.4	0.54	0.03	0.01
	**HX898**	1.72	3.58	0.59	0.02	0
CNP(MMAE); 1 mg/kg MMAE	**HX802**	1.98	3.14	0.49	0.09	0.01
	**HX803**	1.22	5.04	0.42	0.16	0.06
	**HX804**	2.24	3.22	0.72	0.09	0.02
CNP(MMAE); 3 mg/kg MMAE	**HX805**	2.28	2.41	0.58	0.06	0.04
	**HX810**	2.2	2.93	0.77	0.08	0.01
	**HX812**	3.21	4.22	1.05	0.13	0.02

Supplementary Figure 31: Assessment of gross, serologic, and hematologic toxicities of BALB/c mice at 14 days after a single dose administration of free MMAE or coated and MMAE-conjugated nanoparticles (CNP(MMAE)). Female BALB/c mice at 4-6 weeks of age were randomly grouped (n=3 mice per group). Each mouse was treated with a single dose of the following by IP administration: PBS, MMAE (at either 0.25 or 0.5 mg/kg), or CNP (MMAE) (at either a 1 or 3 mg/kg dose equivalent of free MMAE). The mice were monitored and weighed daily; and, they were sacrificed when they exhibited > 15% loss in body weight or at 14 days after administration. The accompanying subfigures depict: A) the daily weights, B) the serology panel for biomarkers of renal function, C) the serology panel for biomarkers of hepatic function, D) the complete blood count (CBC) and E) the white blood cell differential counts; terminal blood draws were performed by cardiac puncture and major organs were harvested for H&E analysis (see Figure S26). White blood cell (WBC), hemoglobin (Hb), hematocrit (Hct), platelets (Plt), neutrophils (Ne), lymphocytes (Ly), monocytes (Mo), eosinophils (Eo), and Basophils (Ba).

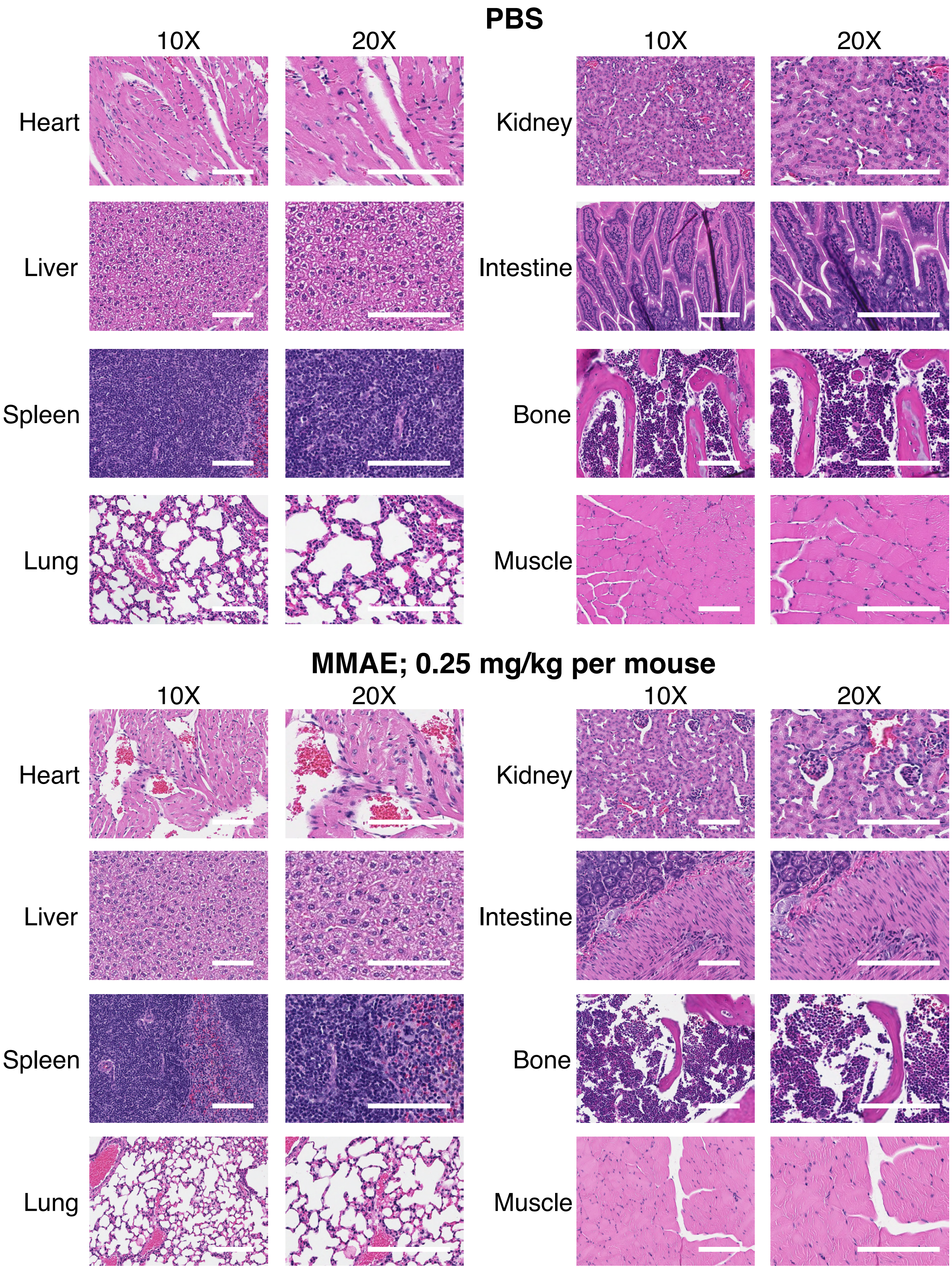
PBS
10X
20X
10X
20X
Heart
Kidney
Liver
Intestine
Spleen
Bone
Lung
Muscle
MMAE; 0.25 mg/kg per mouse
10X
20X
10X
20X
Heart
Kidney
Liver
Intestine
Spleen
Bone
Lung
Muscle

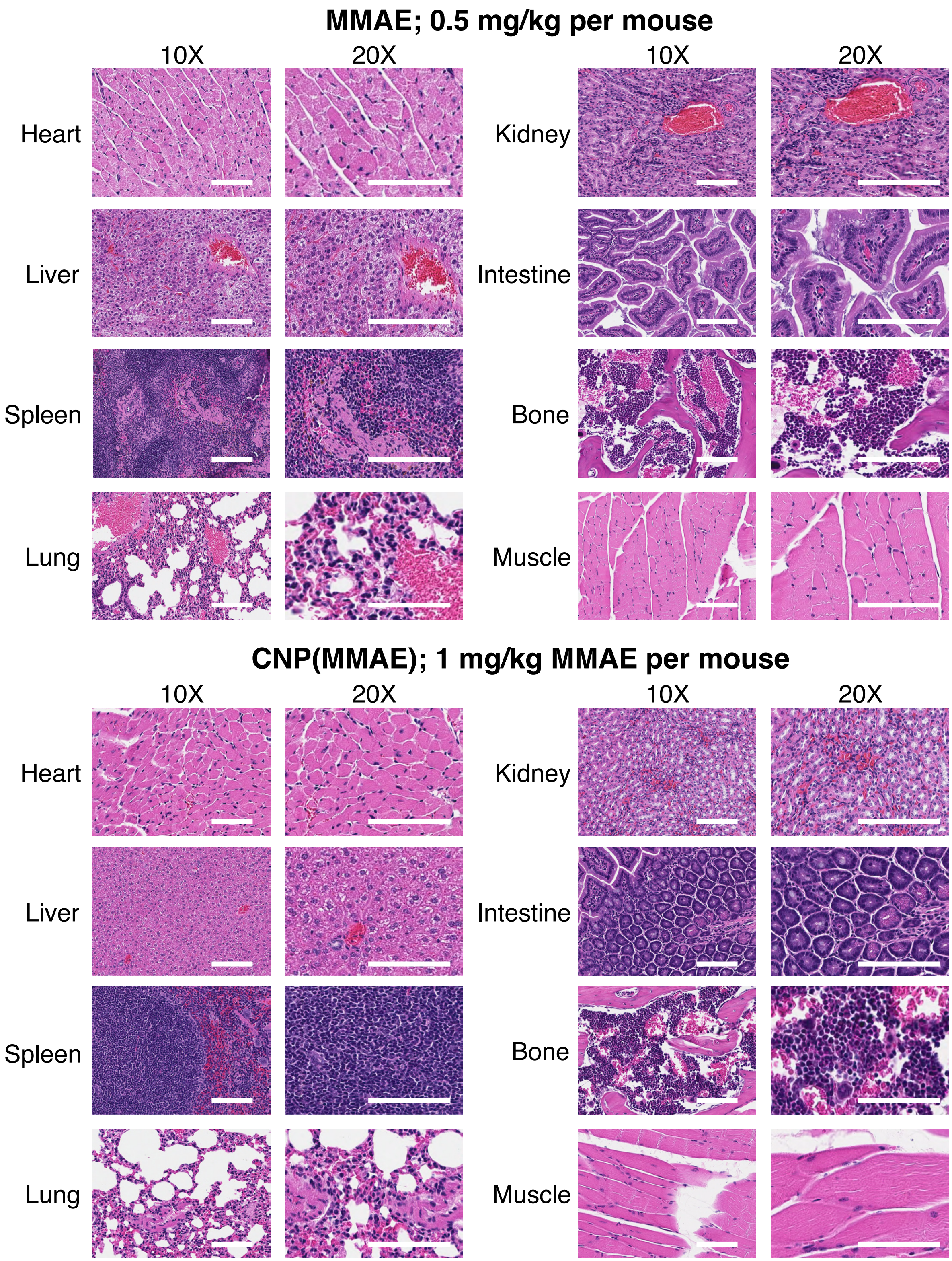
MMAE; 0.5 mg/kg per mouse
10X
20X
10X
20X
Heart
Kidney
Liver
Intestine
Spleen
Bone
Lung
Muscle
CNP(MMAE); 1 mg/kg MMAE per mouse
10X
20X
10X
20X
Heart
Kidney
Liver
Intestine
Spleen
Bone
Lung
Muscle

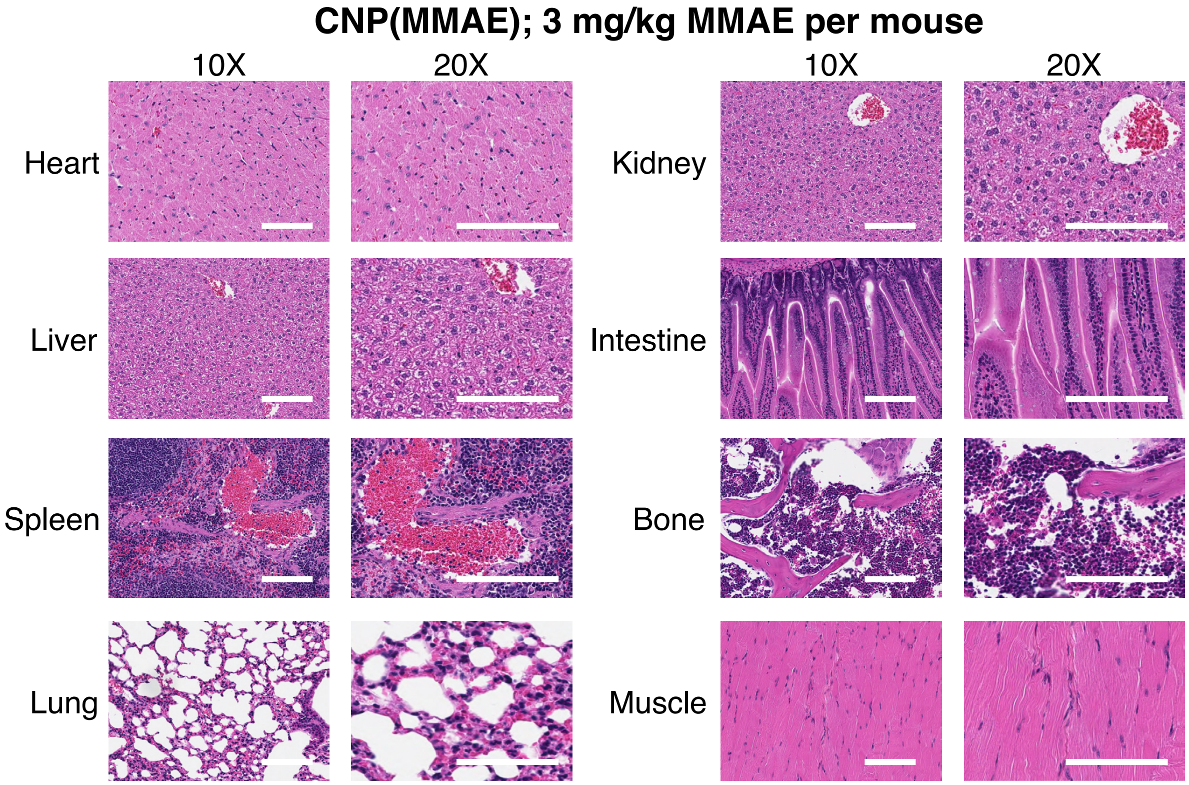

Supplementary Figure 32: H&E of organs harvested from BALB/c mice at 14 days after a single dose administration of free MMAE or coated and MMAE-conjugated nanoparticles (CNP(MMAE)). Female BALB/c mice at 4-6 weeks of age were randomly grouped (n=3 mice per group). Each mouse was treated with a single dose of the following by IP administration: PBS, MMAE (at either 0.25 or 0.5 mg/kg), or CNP (MMAE) (at either a 1 or 3 mg/kg dose equivalent of free MMAE). The mice were monitored and weighed daily; and, they were sacrificed when they exhibited > 15% loss in body weight or at 14 days after administration. Scale bar: 200 μm.

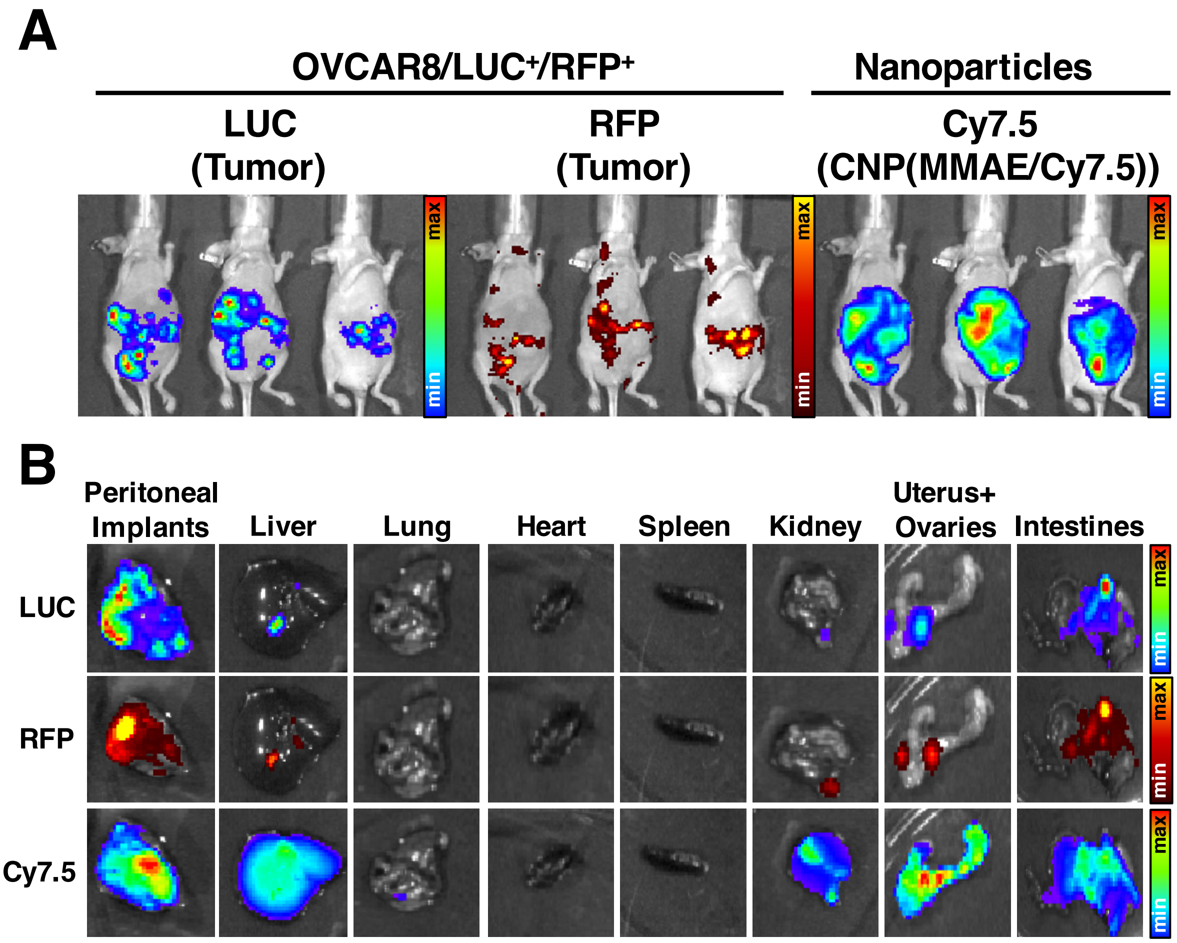

Supplementary Figure 33: Biodistribution of coated and MMAE-conjugated nanoparticles (CNP(MMAE)) in a disseminated OVCAR8 cell-line xenograft model of ovarian cancer as assessed by optical imaging. LUC^+/RFP^+ OVCAR8 cells (0.8 million cells/mouse) were introduced via IP injection into female NCr nude mice and were allowed to grow until the LUC signals from their tumors reached $1x10^7$ radians (photons/sec/cm^2/surface area; ~3 weeks). Thereafter, the animals were administered Cy7.5-conjugated CNP(MMAE) (3 mg/kg dose equivalent of MMAE) by IP injection. After 24 h, bioluminescence (LUC) and fluorescence imaging (RFP and Cy7.5) commenced, using an IVIS Caliper LS system (auto exposition mode). The *in vivo* biodistribution of CNP(MMAE/Cy7.5) was observed by gating on the Cy7.5 channel (λ_{ex} = 740 nm; λ_{em} = 820 nm). The relative locations of the tumors were visualized via *in vivo* imaging in the RFP channel (λ_{ex} = 540 nm; λ_{em} = 580 nm) and from their LUC signals upon injection of d-luciferin (50 mg/kg). Upon completion of *in vivo* imaging, the mice were sacrificed and their organs were harvested and imaged *ex vivo,* using the same imaging parameters. The average photon flux in radians for the different reporter signals in each excised organ were quantified by gating on regions of interest, using Living Image Software V.4.5.2, for 3 separate mice that were similarly processed. The relative signal distribution intensities from each organ (after normalization to the signal intensities recorded from the intestines, which were the major organs from which peritoneal tumor implants were explanted) are reported in Figure 4B in the main manuscript.

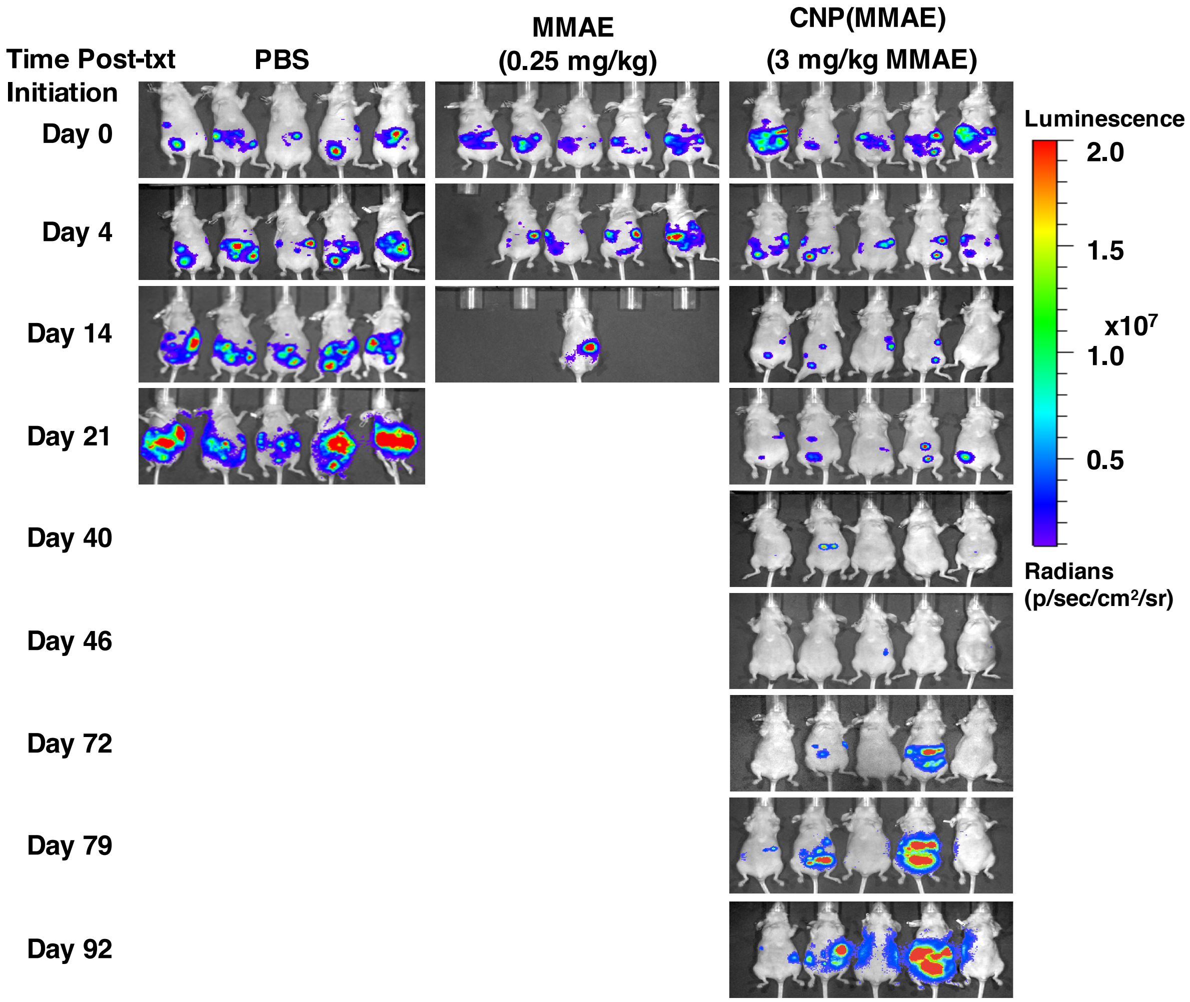

Supplementary Figure 34: Therapeutic efficacy of coated and MMAE-conjugated nanoparticles (CNP(MMAE)) in a disseminated OVCAR8 cell-line xenograft model of ovarian cancer. LUC^+/RFP^+ OVCAR8 cells (0.8 million cells/mouse) were introduced via IP injection into female NCr nude mice and were allowed to grow until the BLI signals from their tumors reached $1x10^5$ radians (photons/sec/cm^2/surface area; ~1 week). Thereafter, the animals were administered free MMAE (0.25 mg/kg), CNP(MMAE) (3 mg/kg dose equivalent of free MMAE) or PBS by once weekly IP injection (on days 0, 7, 14, and 21). The LUC signals emanating from the tumors of the animals were imaged periodically until the animals showed gross signs of toxicity or a loss of 15% in body weight. Changes in signal intensities were compared to baseline, were enumerated by gating on the whole peritoneal cavity (i.e. the area of tumor growth), and were determined by measuring the average photon flux in radians, which enabled normalization for differences in imaging areas between mice and in the same mouse over time.

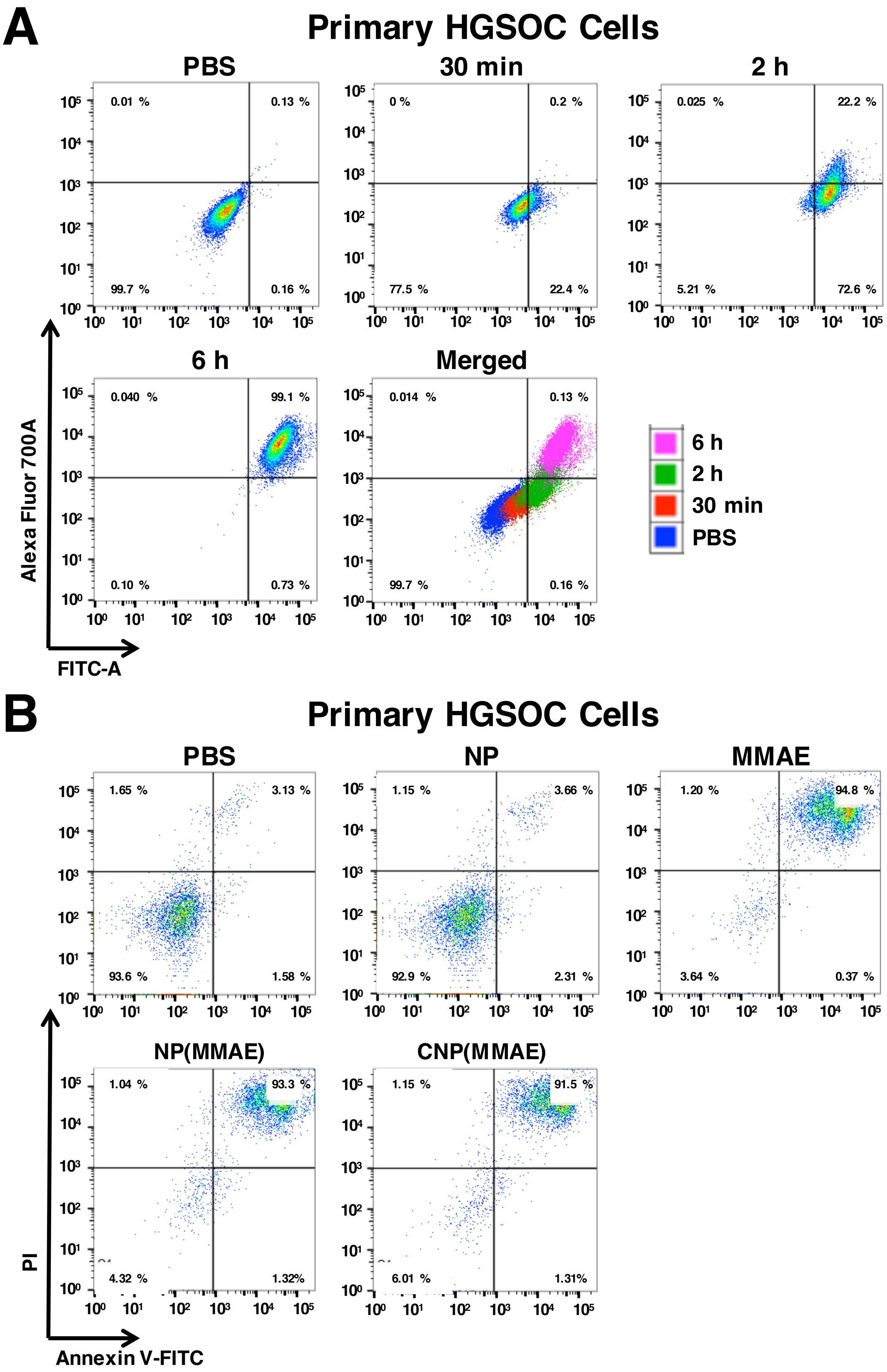
A
Primary HGSOC Cells
PBS
0.01 %
0.13 %
99.7 %
0.16 %
30 min
0 %
0.2 %
77.5 %
22.4 %
2 h
0.025 %
22.2 %
5.21 %
72.6 %
6 h
0.040 %
99.1 %
0.10 %
0.73 %
Merged
0.014 %
0.13 %
99.7 %
0.16 %
6 h
2 h
30 min
PBS
Alexa Fluor 700A
FITC-A
B
Primary HGSOC Cells
PBS
1.65 %
3.13 %
93.6 %
1.58 %
NP
1.15 %
3.66 %
92.9 %
2.31 %
MMAE
1.20 %
94.8 %
3.64 %
0.37 %
NP(MMAE)
1.04 %
93.3 %
4.32 %
1.32%
CNP(MMAE)
1.15 %
91.5 %
6.01 %
1.31%
PI
Annexin V-FITC

Supplementary Figure 35: Intracellular uptake and apoptosis of primary patient-derived, platinum-resistant, and high-grade serous ovarian cancer (HGSOC) cells after *in vitro* treatment with various MMAE formulations. A) Cellular uptake of CNP(MMAE) as assessed by 2D flow cytometry. Primary cells from a patient with platinum-resistant and HGSOC were seeded in six-well plates (5 $\times 10^5$ cells/well) and were subsequently incubated either with PBS (control) or with dual-fluorophore-labeled, coated, and MMAE-containing nanoparticles (5'-FAM-CNP(MMAE/Cy5.5)) for 30 min, 2 h or 6 h prior to flow cytometry measurements. Each component of this coated nanoparticle structure was independently monitored at each time point, gating on the fluorescence of the 5-FAM-conjugated $mPEG_{114}$-b-PGA_{30} polymer (5'-FAM (Coat); FITC-A channel) and on fluorescence of the Cy5.5-lableled NP(MMAE) (NP(MMAE/Cy5.5); AlexaFluor 700A channel). Note that the concentrations of 5'-FAM and Cy5.5 were held constant throughout (both were present at 1 μg/mL). By plotting each time point on the same graph ("merged"), the time dependent increases in the percentages of cells labeled with both 5'-FAM and Cy5.5 (i.e. cells with both AlexaFluor700 and FITC-A high signals) confirmed the co-localization of these two fluorophore populations and supported the stability of the CNP(MMAE) structure during the cellular internalization process. B) Flow cytometry-based apoptosis assay performed on primary HGSOC cells after *in vitro* treatment with various MMAE-containing and control formulations. The primary cells were seeded in 6-well plates (4×10^5 cells/well) and were allowed to equilibrate for 24 h. The cells were then treated with the free drug formulation of MMAE (MMAE), NP(MMAE), or CNP(MMAE) for 48 h and at a fixed MMAE concentration (100 nM). PBS and empty uncoated nanoparticles (NP) served as additional control treatments and were incubated with the cells at equal volumes and/or polymer concentrations. Following the 48 h of incubation with each treatment group, the fractions of apoptotic cells were detected by flow cytometry, using the Annexin V-FITC Apoptosis Detection Kit I.

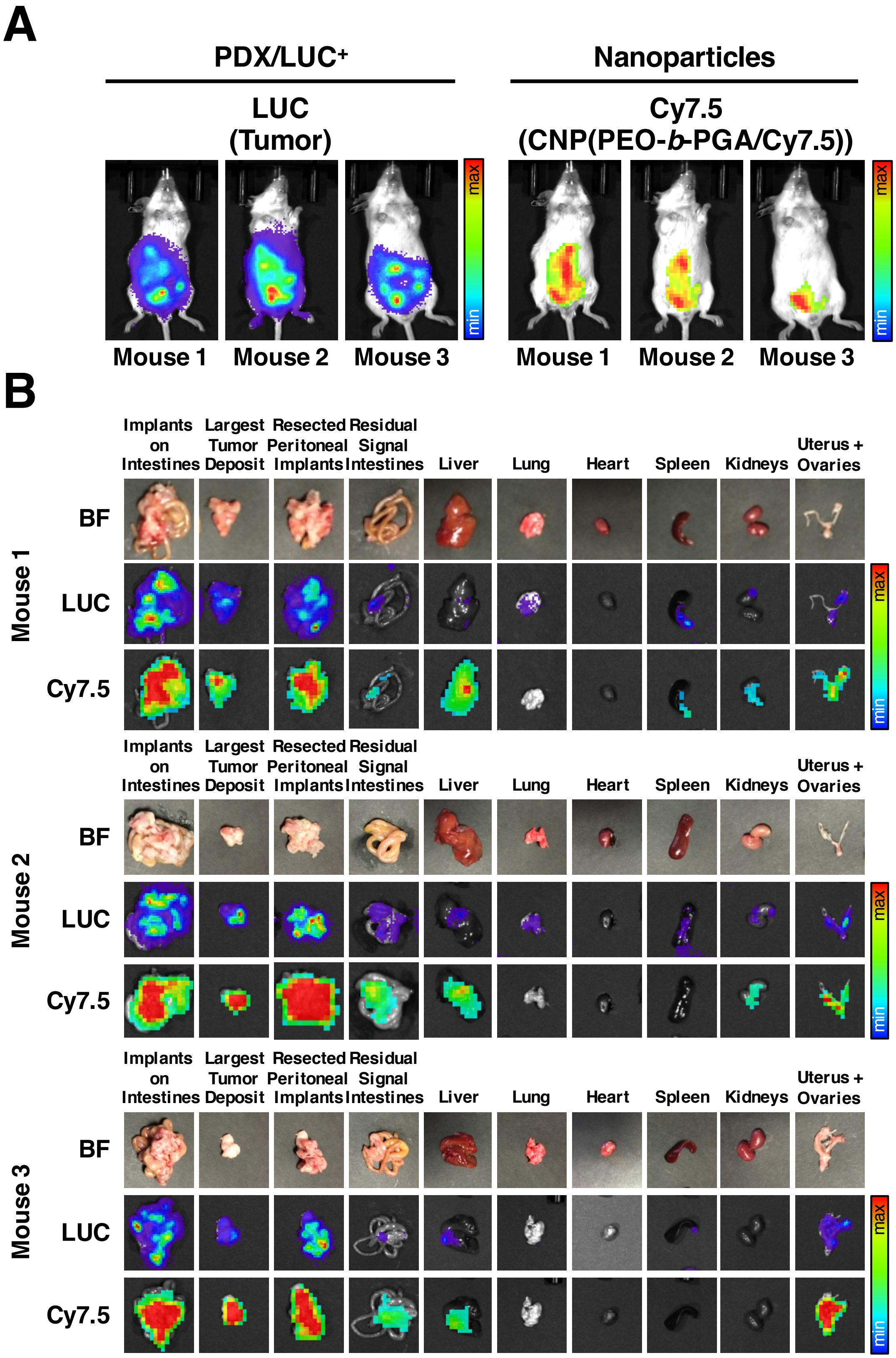
A
PDX/LUC+
LUC
(Tumor)
Nanoparticles
Cy7.5
(CNP(PEO-b-PGA/Cy7.5))
max
min
Mouse 1
Mouse 2
Mouse 3
B
Implants on Intestines
Largest Tumor Deposit
Resected Peritoneal Implants
Residual Signal Intestines
Liver
Lung
Heart
Spleen
Kidneys
Uterus + Ovaries
BF
LUC
Cy7.5

Supplementary Figure 36: Biodistrubution of coated and MMAE-conjugated nanoparticles (CNP(MMAE)) in a PDX model of advanced-stage, platinum-resistant, and high-grade serous ovarian cancer (HGSOC) as assessed by optical imaging. LUC^+ primary HGSOC cells (4 million cells/mouse) were introduced via IP injection into C.B-17/Icr-SCID/Sed mice and were allowed to grow until the LUC signals from their tumors reached $1x10^7$ radians (photons/sec/cm^2/surface area; ~2 weeks). Thereafter, the animals were administered Cy7.5-conjugated CNP(MMAE) (3 mg/kg equivalent dose of MMAE) by IP injection. After 24 h, bioluminescence (LUC) and fluorescence imaging (Cy7.5) commenced, using an IVIS Caliper LS system (auto exposition mode). The *in vivo* biodistribution of CNP(MMAE/Cy7.5) was observed by gating on the Cy7.5 channel (λ_{ex} = 740 nm; λ_{em} = 820nm). The relative location of the tumors was visualized via *in vivo* imaging of their LUC signals upon injection of d-luciferin (50 mg/kg). Upon completion of *in vivo* imaging, the mice were sacrificed and their organs were harvested and imaged *ex vivo* using the same parameters. The average photon flux in radians for the different reporter signals in each excised organ were quantified by gating on regions of interest, using Living Image Software V.4.5.2, for 3 separate mice that were similarly processed. The relative signal distribution intensities from each organ (after normalization to the signal intensities recorded from the intestines, which were the major organs from which peritoneal tumor implants were explanted) are reported in Figure 5C in the main manuscript. Note that even after resection of peritoneal implants from the serosal surfaces of the intestines of each mouse, a residual signal remained that was attributed to the presence of microscopically infiltrating tumor cells.

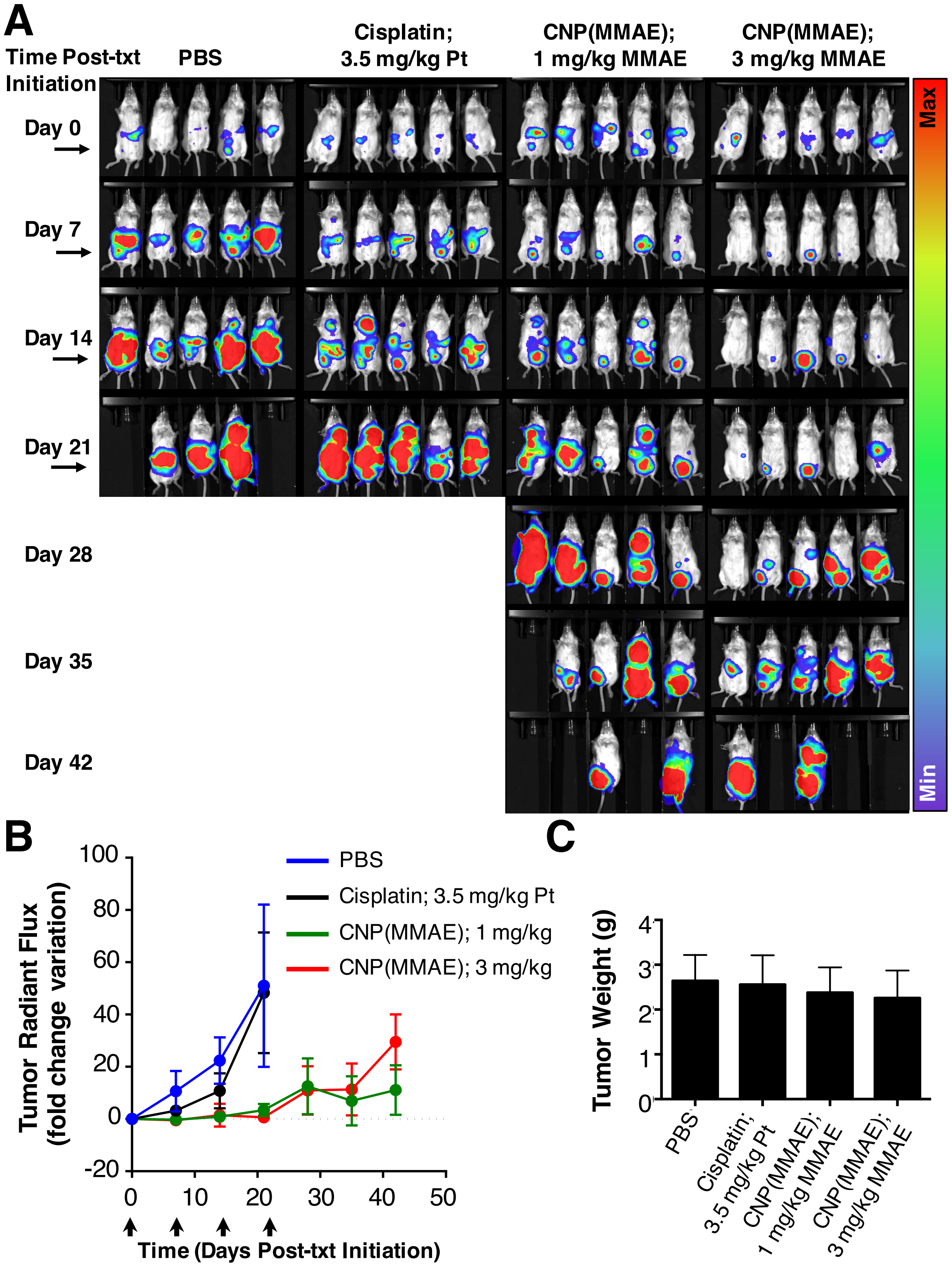
A
Time Post-txt Initiation
PBS
Cisplatin; 3.5 mg/kg Pt
CNP(MMAE); 1 mg/kg MMAE
CNP(MMAE); 3 mg/kg MMAE
Day 0
Day 7
Day 14
Day 21
Day 28
Day 35
Day 42
Max
Min
B
Tumor Radiant Flux (fold change variation)
Time (Days Post-txt Initiation)
PBS
Cisplatin; 3.5 mg/kg Pt
CNP(MMAE); 1 mg/kg
CNP(MMAE); 3 mg/kg
C
Tumor Weight (g)
PBS
Cisplatin; 3.5 mg/kg Pt
CNP(MMAE); 1 mg/kg MMAE
CNP(MMAE); 3 mg/kg MMAE

Supplementary Figure 37: Therapeutic efficacy of coated and MMAE-conjugated nanoparticles (CNP(MMAE)) in a disseminated PDX model of platinum-resistant and high-grade serous ovarian cancer (HGSOC). LUC^+ primary HGSOC cells (4 million cells/mouse) were introduced via IP injection into C.B-17/Icr-SCID/Sed mice and were allowed to grow until the LUC signal from their tumors reached $1x10^7$ radians (i.e. $1x10^7$ photons/sec/cm^2/surface area; ~2 weeks). Thereafter, the animals were administered free MMAE (0.25 mg/kg), CNP(MMAE) (at either 1 or 3 mg/kg dose equivalent of free MMAE), cisplatin at its maximally tolerated dose (3.5 mg/kg equivalent of platinum) or PBS by once weekly IP injection (on days 0, 7, 14, and 21). A) LUC signals emanating from the tumors of the animals were imaged periodically until the animals showed gross signs of toxicity or a loss of 15% in body weight; note that the black arrows indicate the timing of each dose of treatment. B) Changes in the average signal intensities of tumors from mice treated with the various experimental groups. Changes in signal intensities were compared to baseline, were enumerated by gating on the whole peritoneal cavity (i.e. the area of tumor growth), and were determined by measuring the average photon flux in radians, which enabled normalization for differences in imaging areas between mice and in the same mouse over time. C) The average and distribution of the weights of the tumors collected from animals in each treatment group at the time of sacrifice.

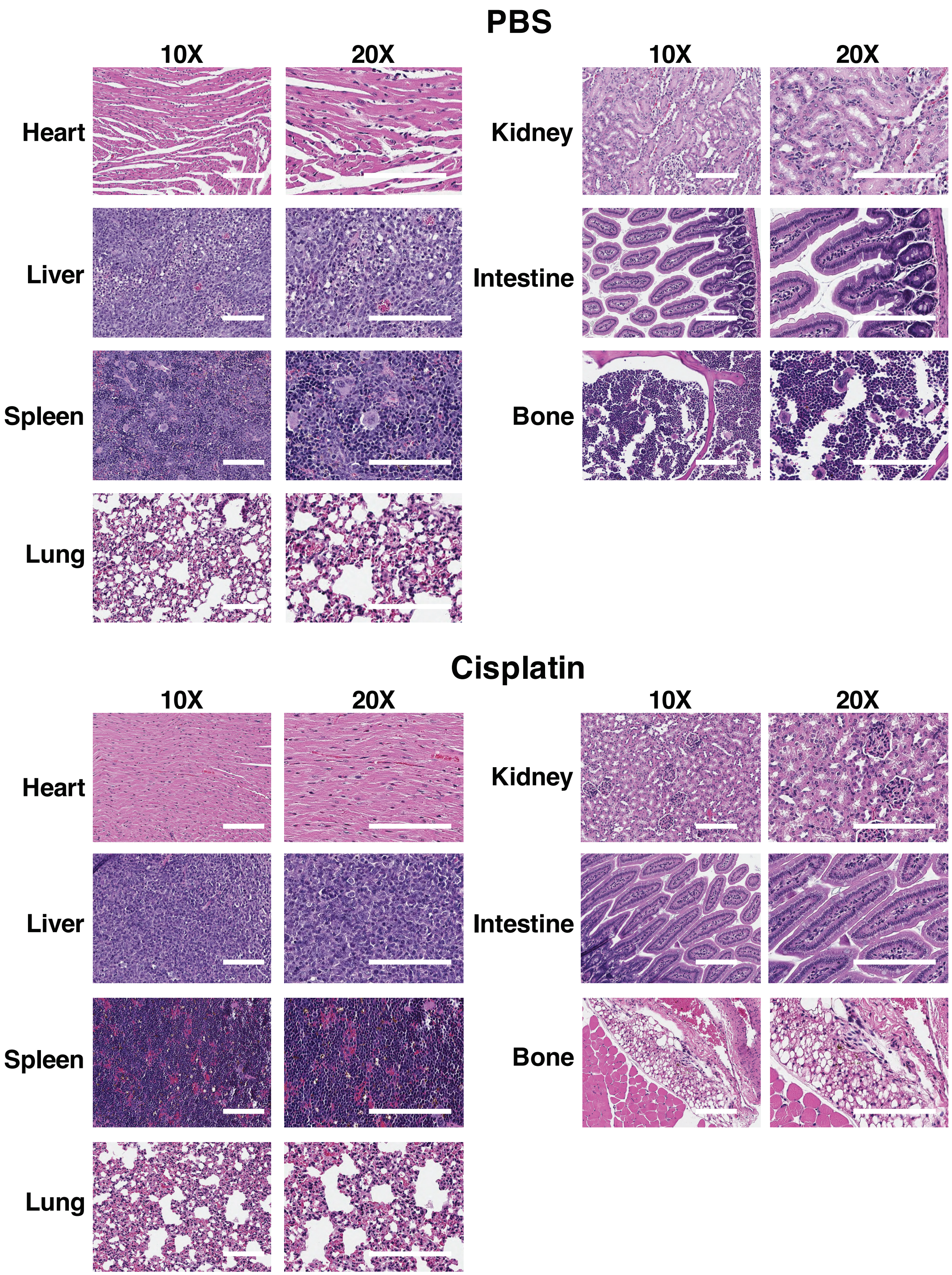
PBS
10X
20X
10X
20X
Heart
Kidney
Liver
Intestine
Spleen
Bone
Lung
Cisplatin
10X
20X
10X
20X
Heart
Kidney
Liver
Intestine
Spleen
Bone
Lung

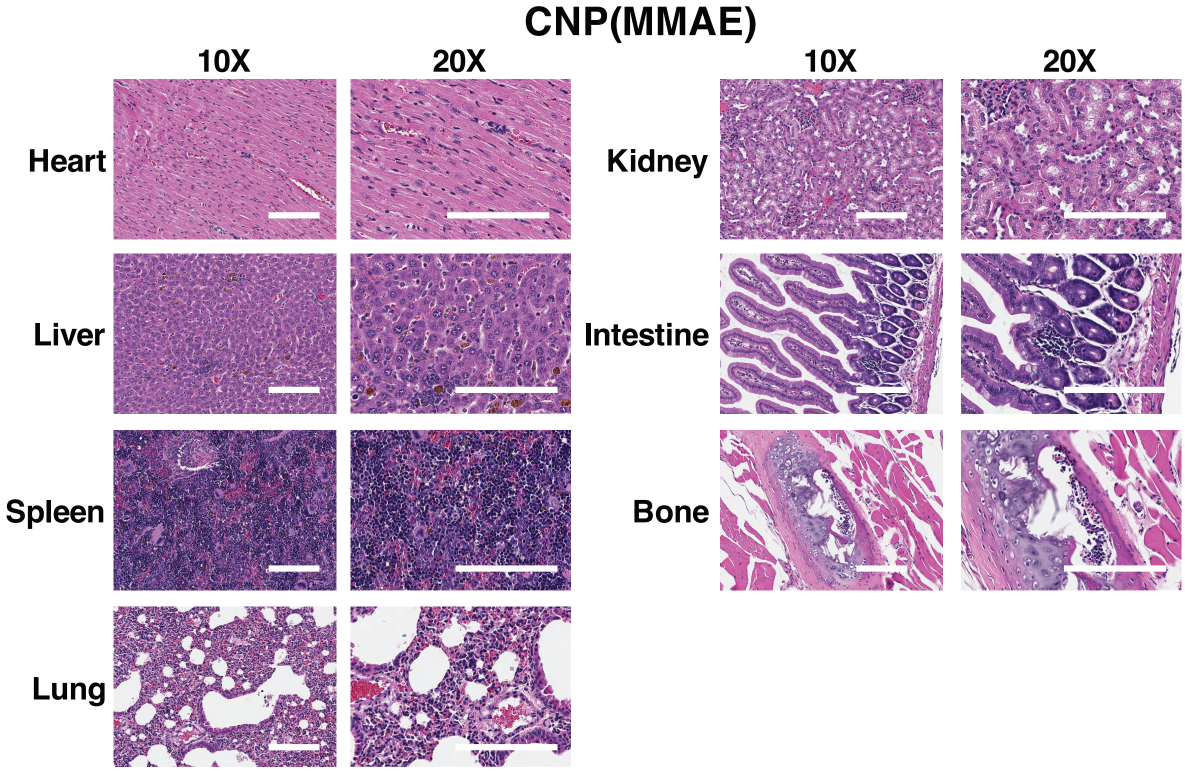

Supplementary Figure 38: Histology of H&E stained sections from major organs of PDX mice in the therapeutic efficacy study and after treatment with PBS, cisplatin or CNP(MMAE). Each mouse was treated with up to 4 weekly doses of each agent. Upon animal sacrifice, which occurred when the mice appeared moribund, major organs were collected for histology analysis after H&E staining. Scale bar = 200 μm.

SUPPLEMENTARY REFERENCES

1. Qi, R. *et al.* Biodegradable copolymers with identical cationic segments and their performance in siRNA delivery. *Journal of controlled release : official journal of the Controlled Release Society* **159**, 251-260, doi:10.1016/j.jconrel.2012.01.015 (2012).
2. Kanayama, N. *et al.* A PEG-based biocompatible block catiomer with high buffering capacity for the construction of polyplex micelles showing efficient gene transfer toward primary cells. *ChemMedChem* **1**, 439-444, doi:10.1002/cmdc.200600008 (2006).
3. Hamblett, K. J. *et al.* Effects of drug loading on the antitumor activity of a monoclonal antibody drug conjugate. *Clin Cancer Res* **10**, 7063-7070, doi:10.1158/1078-0432.CCR-04-0789 (2004).
4. Doronina, S. O. *et al.* Enhanced activity of monomethylauristatin F through monoclonal antibody delivery: effects of linker technology on efficacy and toxicity. *Bioconjug Chem* **17**, 114-124, doi:10.1021/bc0502917 (2006).
5. Francisco, J. A. *et al.* cAC10-vcMMAE, an anti-CD30-monomethyl auristatin E conjugate with potent and selective antitumor activity. *Blood* **102**, 1458-1465, doi:10.1182/blood-2003-01-0039 (2003).
6. Doronina, S. O. *et al.* Development of potent monoclonal antibody auristatin conjugates for cancer therapy. *Nat Biotechnol* **21**, 778-784, doi:10.1038/nbt832 (2003).
